# Ultrahigh-sensitivity adaptive solar-blind organic neuromorphic sensors for robust corona discharge monitoring

**DOI:** 10.1038/s41467-026-77523-y

**Published:** 2026-09-04

**Authors:** Yang Liu, Yulin Sha, Xiaonan Ma, Hongkun Tian, Deyang Ji, Wenping Hu

**Affiliations:** 1 Institute of Molecular Aggregation Science, Tianjin University, Tianjin, China; 2 Institute of Molecular Plus, Tianjin University, Tianjin, China; 3 State Key Laboratory of Polymer Physics and Chemistry, Changchun Institute of Applied Chemistry, Chinese Academy of Sciences, Changchun, China; 4 Key Laboratory of Organic Integrated Circuit, Ministry of Education, Tianjin University, Tianjin, China; 5 State Key Laboratory of Advanced Materials for Intelligent Sensing, Tianjin University, Tianjin, China

**Keywords:** Electronic devices, Electrical and electronic engineering

## Abstract

Low‑cost and high‑efficiency corona-discharge detection is essential for safe high‑voltage transmission operation, yet state‑of‑the‑art solar‑blind ultraviolet (SBUV) photodetectors for corona monitoring suffer from insufficient sensitivity, poor stability and weak anti‑interference capability. Herein, we demonstrate a high-sensitivity adaptive organic neuromorphic SBUV sensor via low‑cost solution‑shearing. The device achieves ultraweak detection down to 60 nW cm^−2^, with key figures‑of‑merit (photosensitivity: 1.1×10^7^, photoresponsivity: 4.6×10^7 ^A W⁻^1^, specific detectivity: 2.9×10^18^ Jones and external quantum efficiency: 2.2×10^8^%), all outperforming state-of-the-art SBUV detectors. The sensor exhibits strong SBUV spectral selectivity, featuring a 25 ms photoresponse and favorable stability after one‑year storage. Enabled by integrated sensing‑storage‑calculation neuromorphic mechanism, it enables reliable scotopic/photopic adaptive detection and rapid patterning within 2 s at 100% analog recognition accuracy. Practical corona‑discharge simulations confirm its high anti‑interference performance, precise classification and reliable early‑warning capability. This work provides a feasible proof‑of‑concept for adaptive organic neuromorphic SBUV sensors toward intelligent corona‑discharge monitoring in smart power systems.

## Abstract (web summary)

Ultraviolet detection is critical for monitoring corona discharges in high-voltage power grids. Addressing this, Liu et al. report a low-cost organic solar-blind ultraviolet sensor that detects ultra-weak signals via adaptive, rapid processing, facilitating anti-interference early warnings for smart power systems.

## Introduction

Solar-blind ultraviolet (SBUV) detection has attracted substantial attention across both military and civilian domains^1^, with its application scenarios encompassing missile warning and tracking, corona discharge monitoring, fire warning and detection, and solar-blind encrypted communication^2–5^. Thereinto, corona discharge, an inevitable electrical phenomenon occurring in high-voltage power equipment, poses a severe threat to the safety and stability of power grids^6^. It not only accelerates the aging and degradation of insulation materials but also triggers potential fire hazards and electromagnetic interference, leading to enormous economic losses and even catastrophic power outages^7–9^. Therefore, the accurate, real-time, and early detection of corona discharge is of paramount importance for safeguarding the reliable operation of power systems and reducing maintenance costs. Traditional corona detection methods relying on electrical parameters, electromagnetic wave radiation and acoustic sensing are susceptible to external disturbances such as environmental noise, which may lead to false alarms and thus fail to achieve efficient, reliable and real-time detection^10,11^. To this end, SBUV detectors have emerged as a preferred approach due to their inherent advantages of anti-interference, high specificity, and non-contact measurement^12–14^, which can directly capture the SBUV signals accompanying corona discharges, efficiently provide accurate early warning information, and thereby underpin the prevention of catastrophic incidents such as equipment damage and electrical fires^4,5,15,16^. To date, solar-blind photodetectors for corona detection are mainly categorized into inorganic and organic-based devices^15,17^. Among these, inorganic detectors represented by wide-bandgap semiconductors (e.g., Ga_2_O_3_^18^, SiC^19^ and AlGaN^20^) exhibit high charge carrier mobility, excellent thermal stability and high response speed^21^. Nevertheless, their inherent drawbacks of high manufacturing costs and sophisticated fabrication processes (e.g., high temperature, high pressure, and ultra-high vacuum) have severely impeded their practical deployment^22–25^. By contrast, organic semiconductors have garnered increasing attention for their distinct advantages, such as low cost, solution processing capability, large-area fabrication potential, and lightweight properties, which provide a foundation for developing high-performance SBUV detectors^26–31^. However, most reported organic devices suffer from low photosensitivity, poor interference resistance and inferior stability. Additionally, their performance metrics (e.g., detectivity, responsivity, and on/off ratio) are yet to meet the requirements for high-precision corona detection, limiting their competitiveness in practical scenarios^32–34^.

To overcome the limitations of organic solar-blind photodetectors and develop high-performance devices for efficient corona detection, we report herein an ultra-high-sensitivity organic-based phototransistor fabricated via a low-cost solution shearing method. This solution-based processing strategy not only retains the cost advantage and scalability of organic materials but also enables precise control over the film morphology and molecular packing, which are crucial for optimizing optoelectronic performance. The as-fabricated organic phototransistor exhibits ultra-high sensitivity towards solar-blind wavelengths, with illumination detection down to 60 nW cm^−2^, while simultaneously ensuring high photosensitivity (*P*) (1.1 × 10^7^), high photoresponsivity (*R*) (4.6 × 10^7^ A W^−1^), high specific detectivity (D^*^) (2.9 × 10^18^ Jones), external quantum efficiency (EQE) of 2.2 × 10^8^% and precise spectral selectivity at 255 nm, surpassing those of previously reported solar-blind photodetectors. This detector exhibits favorable stability and a fast response of 25 ms, while maintaining competitive photoelectric properties even after one year of storage, which strongly supports its application prospects in practical detection. Additionally, the detector can function as a photonic synapse transistor to achieve integrated sensing-storage-computing capabilities. Its dark-adaptation and light-adaptation capabilities effectively broaden the detector’s sensing range under varying illumination conditions, offering avenues for innovative applications in advanced machine vision technology. Leveraging the detector’s efficient information processing capability, we integrated the device into a corona discharge monitoring system for simulation tests to verify its practical application value. The results demonstrate that even under intense background interference, the detector reliably identifies faint corona signals with high signal-to-noise ratio and accuracy. This research provides an efficient, high-performance solution for corona discharge monitoring in high-voltage transmission equipment, significantly advancing the application of organic optoelectronic technology in power equipment safety inspection.

## Results

### Scene construction and material characterization

**Fig. 1:**
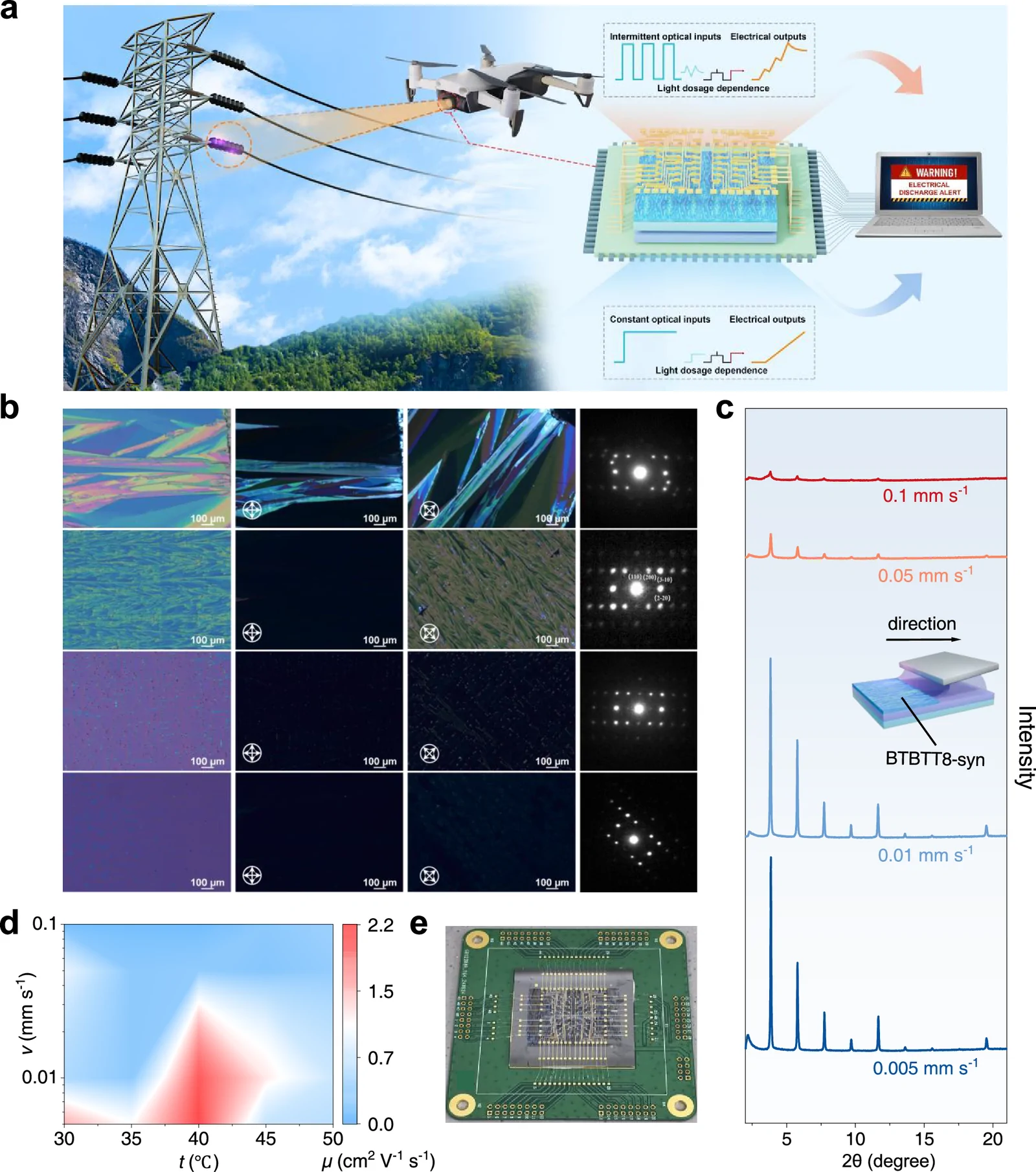
Characterization and electric properties of BTBTT8-syn phototransistor. **a** Schematic diagram of an organic photodetector based on BTBTT8-syn for deep-ultraviolet high-voltage discharge monitoring and early warning. **b** Optical microscopy (OM, left), polarized optical microscopy (POM, center: 0°, right: 45°), and selected area electron diffraction (SAED) patterns of BTBTT8-syn films solution-sheared at 40 °C with shear speeds of 0.005, 0.01, 0.05 and 0.1 mm s^−1^. **c** X-ray diffraction (XRD) characterization of films fabricated at various blade-coating speeds, with a blade-coating schematic. **d** Correlation between device mobility with substrate temperature and coating speed. **e** Physical diagram of BTBTT8-syn phototransistor array. Source data are provided as a Source Data file.

Based on the deep ultraviolet (DUV) emission characteristics of discharges in high-voltage transmission equipment, we have developed a DUV-based phototransistor, which is designed to monitor high-voltage discharges and issue early warnings by converting optical signals into electrical signals (Fig. 1a). As presented in Supplementary Fig. 1, 2-(5-octylthiophen-2-yl) [1] benzothieno[3,2-b][1] benzothiophene (BTBTT8-syn) was selected as the active layer, attributed to its strong absorption in the DUV region (200–280 nm), with a prominent absorption peak at 255 nm. Subsequently, the BTBTT8-syn film was fabricated via the solution-shearing method, and the influence of substrate temperature and blade coating speed on the crystallinity of the semiconductor film was systematically investigated. As shown in Supplementary Fig. 2, films prepared at 30 and 35 °C displayed island-like growth with isolated, inhomogeneous crystallites, abundant grain boundaries and severe pinholes, all of which strongly hindered charge transport. By contrast, elevated temperatures (45 and 50 °C) disturbed controlled crystallization: at 50 °C and low shearing speeds, excessive phase separation and over-crystallization produced disordered domains and higher shearing rates only improved uniformity at the cost of drastically reduced crystallinity, favoring an amorphous structure. Notably, 40 °C afforded thin films with high crystallinity, favorable molecular orientation, and excellent uniformity (Fig. 1b). At a low blade coating speed (0.005 mm s^−1^, top panels in Fig. 1b), the semiconductor crystals aligned and grew along the direction of the blade movement, forming a continuous multilayer polycrystalline film. Polarizing optical microscope (POM) images acquired at 0° and 45° showed no significant differences, confirming the polycrystalline nature of the film. Furthermore, selected area electron diffraction (SAED) patterns provided additional evidence for its polycrystalline structure (the top‑right panel in Fig. 1b and Supplementary Fig. 3a–c). When the blade coating speed was increased to 0.01 mm s^−1^, a uniform, continuous dendritic single-crystal film was formed (upper‑mid‑left panel in Fig. 1b), accompanied by a distinct extinction phenomenon under POM observation. SAED patterns revealed diffraction spots corresponding to the (110), (200), (3–10), and (2–20) lattice planes, further verifying the single-crystal structure (upper‑mid‑right panel Fig. 1b and Supplementary Fig. 3d–f). With a further increase in blade coating speed, the solvent evaporated excessively rapidly, leading to unguided nucleation during film formation. This leads to discontinuous films, cracks and sparse crystalline domains (lower‑mid and bottom panels in Fig. 1b and Supplementary Fig. 3g–l). The crystallinity of the as-prepared films was characterized by X-ray diffraction (XRD) (Fig. 1c) and grazing incidence wide-angle X-ray scattering (GIWAXS) (Supplementary Fig. 4). Consistent with the morphological observations, both characterization results demonstrated that the crystallinity of the films gradually decreases with increasing blade coating speed. Specifically, when the speed was elevated to 0.05 and 0.1 mm s^−1^, the films exhibited weak crystallization signals, with no distinct diffraction peaks detectable.

### Electrical and optical properties of organic film devices

Furthermore, we systematically characterized the charge transport properties of the devices fabricated at different substrate temperatures and blade coating speeds. The transfer curves and output curves of each device are presented in detail in Supplementary Figs. 5 and 6. The influence of blade-coating speed (*v*) and substrate temperature (*t*) on device mobility (*μ*) is shown in Fig. 1d. The mobility increases with rising temperature to a maximum value (*μ* = 2.22 cm^2^ V^−1^ s^−1^) at 40 °C under low coating speed, followed by a decrease upon further temperature elevation. Combined with morphological characterization, excessively high blade-coating speeds suppress the ordered molecular arrangement, while overly high substrate temperatures tend to introduce defects, both of which collectively determine the charge transport properties of the films. Therefore, we focused on the device characteristics at a substrate temperature of 40 °C (Fig. 1e). The results demonstrate that both the charge carrier mobility and the on-off ratio decrease with increasing blade coating speed at a substrate temperature of 40 °C, which could be ascribed to different crystalline domains in the formed films. Subsequently, temperature-dependent measurements (from 300 K to 100 K) were performed on all devices under vacuum conditions to clarify their charge transport mechanisms (Supplementary Fig. 7), and the charge transport of all devices followed a thermally activated mode^35^. The activation energy (*E*_*A*_) was calculated and analyzed according to the formula^36^, which revealed that the device fabricated at a speed of 0.005 mm s^−1^ exhibited the lowest activation energy (22.7 meV). This indicates a lower degree of energetic disorder at the interface, which is favorable for charge carrier transport.

**Fig. 2:**
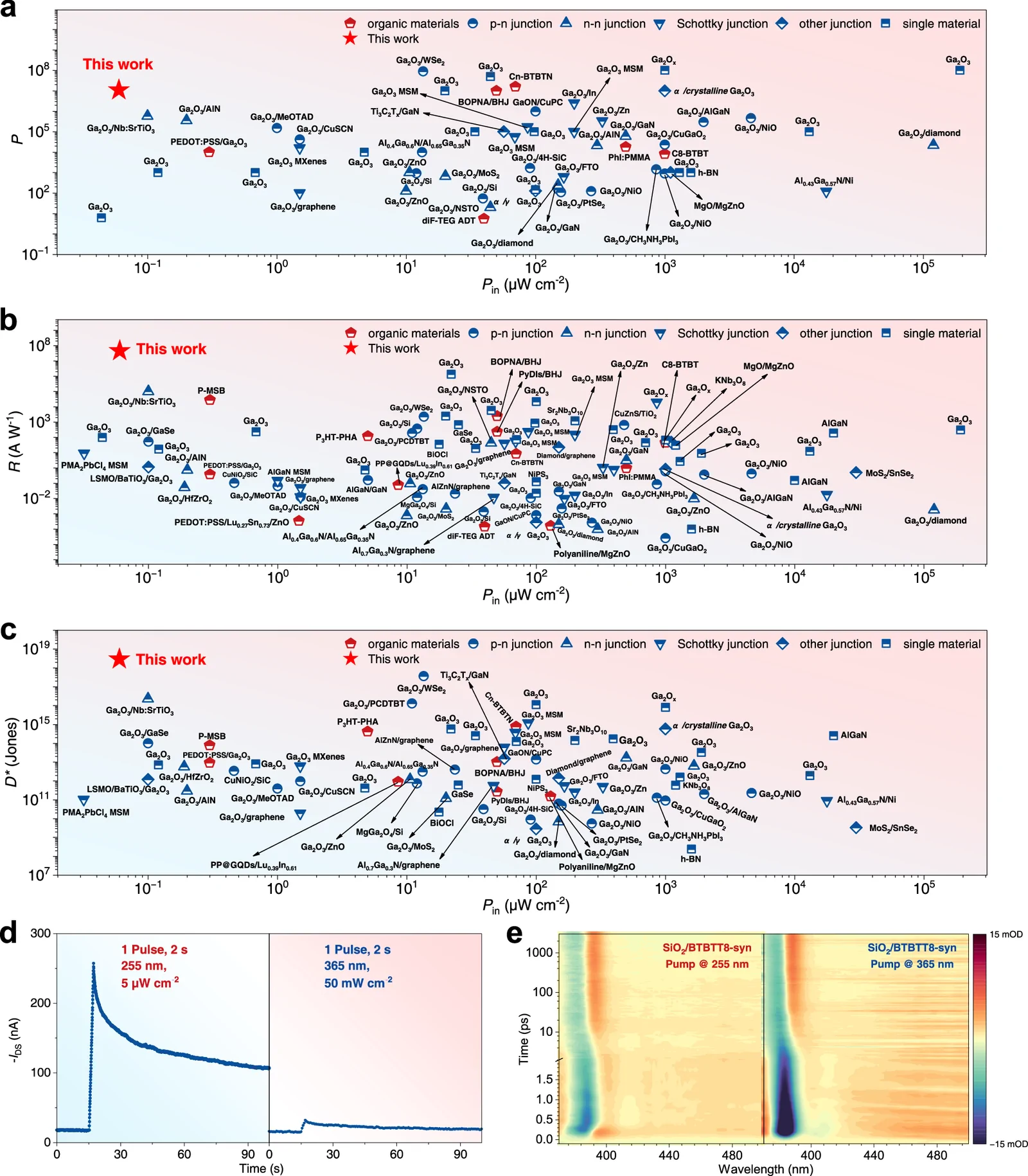
Optical properties of BTBTT8-syn phototransistor. Comparison of the lowest light intensity and **a** photosensitivity (*P*). **b** Photoresponsivity (*R*). **c** Specific detectivity (D^*^) of the reported solar-blind ultraviolet photodetectors in recent years. **d** Comparison of I–t measurement curves at 255 nm and 365 nm. **e** The Femtosecond Transient Absorption Spectroscopy (fs-TAS) pseudocolor plots of SiO_2_/BTBTT8-syn thin films under excitation at pump wavelengths of 255 nm and 365 nm. Source data are provided as a Source Data file.

Based on the superior charge transport properties exhibited by the devices and the characteristic absorption wavelength of BTBTT8-syn semiconductor at 255 nm, we further investigated the optical response of the devices fabricated at different blade coating speeds. Under varying illumination intensities, all four organic phototransistors (OPTs) exhibited responses to low-intensity light, and positive shifts were observed even at an illumination intensity as low as 60 nW cm^−2^ (255 nm) (Supplementary Fig. 8). To obtain more accurate optical parameters, Supplementary Fig. 9 summarizes the noise current curves. Subsequent statistical analysis reveals a negative correlation between blade coating speed and the optical/electrical parameter enhancement (Supplementary Fig. 10). Devices coated at 0.005 mm s^−1^ achieve excellent *P* (1.1 × 10^7^), *R* (4.6 × 10^7^ A W^−1^), D^*^ (2.9 × 10^18^ Jones), and external quantum efficiency (EQE) (2.2 × 10^8^%), along with linear dynamic ranges (LDRs) reaching 138.5 dB. Thus, the fabricated device integrates ultraweak‑light detection capability with favorable optical performance, which ranks among the leading levels of reported SBUV-based detectors (Fig. 2a–c and Supplementary Tables 1–6). Temperature-dependent photoluminescence (PL) spectroscopy was employed to investigate the kinetic characteristics of exciton dissociation at the film interfaces of BTBTT8-syn prepared at different blade coating speeds^37^. The results demonstrate that the film fabricated at the speed of 0.005 mm s^−1^ exhibits a lower exciton binding energy (Eb) of 48.1 meV, which provides strong support for the excellent optical performance of this interface. In contrast, films prepared at higher speeds fail to yield distinct spectral signals due to excessively thin film thickness, which further confirms the differences in the optical performance of films with different interfaces (Supplementary Fig. 11). We next systematically characterize the spectral selectivity of the corresponding photodetector. Although BTBTT8-syn inherently absorbs 365 nm ultraviolet light, the device exhibits nearly negligible photoresponse at this wavelength. A remarkably high-power density threshold is required to generate measurable electrical signals under 365 nm excitation, which yields *P*, *R*, and D^*^ values of 2.1 × 10^5^, 3.2 × 10^3^ A W^−1^ and 4.1 × 10^12^ Jones, respectively (Supplementary Fig. 12). These figures are 2–6 orders of magnitude inferior to the performance metrics obtained under 255 nm solar-blind illumination. Comparative I–t transient measurements (Fig. 2d) further corroborate the device’s favorable wavelength discrimination capability. Weak 255 nm light at only 5 μW cm^−2^ triggers stable, well-resolved photocurrent signals; by contrast, intense 365 nm irradiation up to 50 mW cm^−2^ merely produces faint electrical outputs. To uncover the dynamic carrier origin underlying such drastically divergent photoresponse behaviors, excitation-dependent femtosecond transient absorption spectroscopy (fs-TAS) measurements were performed (Fig. 2e and Supplementary Fig. 13a, b). The ground-state bleaching (GSB) signal at a probe wavelength of 385 nm was extracted to evaluate the lifetime of photogenerated carriers. As shown in Supplementary Fig. 13c, d, time traces of GSB signal can be well-fitted by a sum of three exponential functions, leading to time constants listed in Supplementary Table 7. The extracted fast (τ_1_, τ_2_) and slow (τ_3_) components might associate with the free and trapped carriers, respectively. The GSB signal upon 255 nm excitation exhibits longer carrier lifetimes than corresponding time constants extracted from 365 nm illumination (Supplementary Fig. 13c, d and Supplementary Table 7). This spectroscopic evidence unambiguously verifies the intrinsic preference of BTBTT8-syn for efficient photoresponse under 255 nm solar-blind UV excitation. Taken together, the device achieves precise recognition of 255 nm optical signals with ultrahigh spectral specificity, while effectively suppressing interfering responses originating from 365 nm long-wave ultraviolet light.

Subsequently, we systematically investigated the stability of the OPT devices. The devices retained excellent electrical and optical performance after one year of storage, with slight degradation and overall maintenance of high levels for key parameters (Supplementary Fig. 14). Meanwhile, the device generates an obvious instantaneous surge in photocurrent under irradiation with 25 ms light pulses, with a total recovery time as long as 400 s (Supplementary Fig. 15a), which originates from interfacial trap effect laying a solid physical basis for our subsequent investigation into synaptic mimicking functionalities. To systematically characterize the dynamic photoresponse of the device, periodic I–t measurements were conducted under three different light intensities (Supplementary Fig. 15b–d). In each test, five consecutive 25 ms light pulses were applied with a 20 s dark interval between adjacent pulses, which fully captured the rapid rise of photocurrent upon pulsed illumination as well as the complete slow decay process in the dark.

**Fig. 3:**
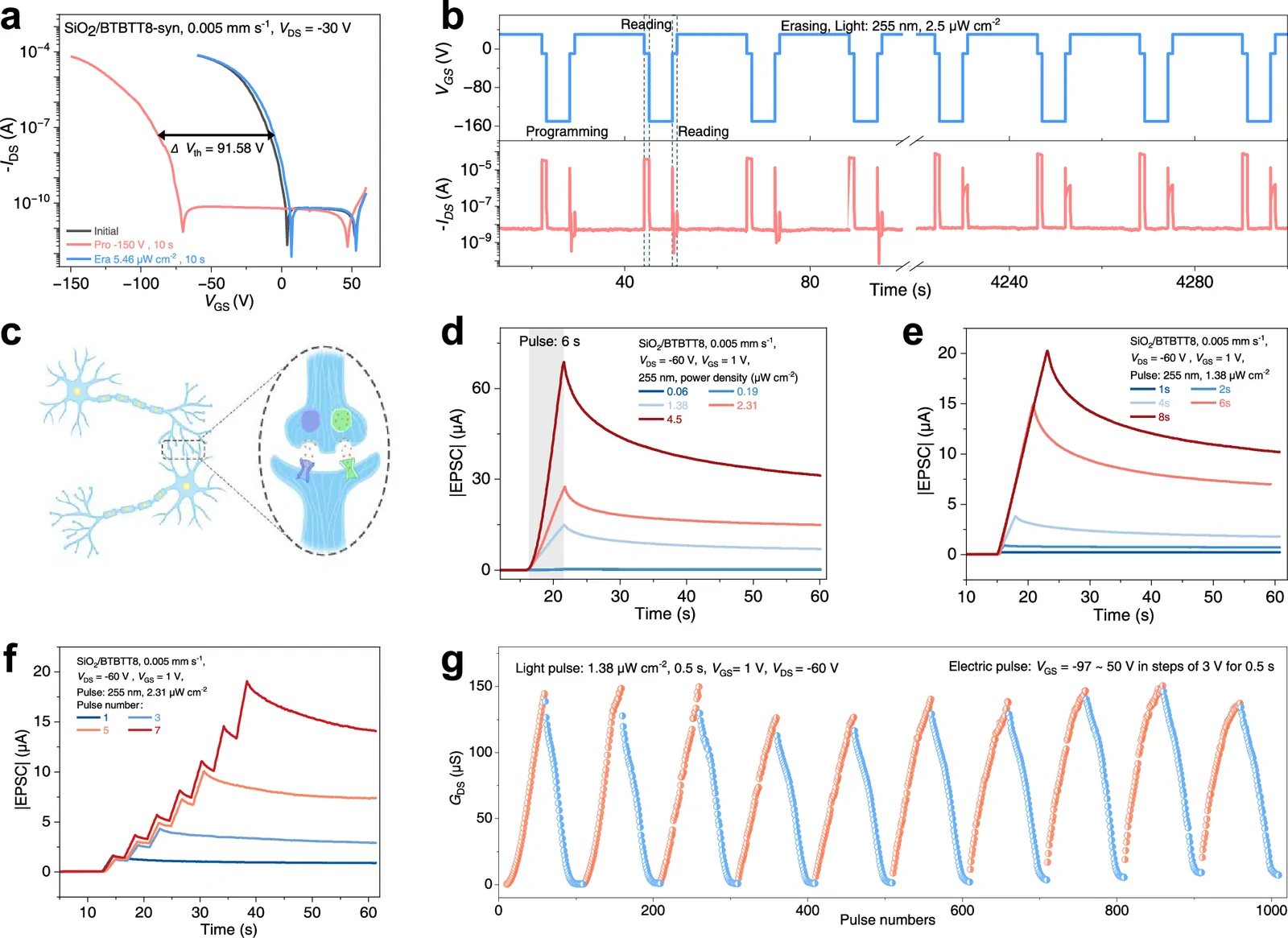
The storage and synaptic characteristics of BTBTT8-syn phototransistor. **a** Electrical programming/optical erasing characteristics of devices based on BTBTT8-syn at a substrate temperature of 40 °C and a blade‑coating speed of 0.005 mm s^−1^. **b** Over 4000 s of Program-Read-Erase-Read cycling of the device. Programming: *V*_DS_ = 0 V, *V*_GS_ = −150 V, 5 s. Reading: *V*_DS_ = −30 V, *V*_GS_ = −10 V, 1 s. Erasing: *V*_DS_ = 0 V, *V*_GS_ = 30 V, 15 s; light: 255 nm, 2.5 μW cm^−2^, 10 s. **c** A schematic diagram of neurotransmitters transmitted through synapses between adjacent neurons. Synaptic performance under different. **d** Optical power densities. **e** Illumination durations. **f** Numbers of light pulses. **g** Cyclic test of 10 consecutive pulse cycles of long-term potentiation/long-term depression (LTP/LTD). The continuous conductance change curve of each LTP/LTD consists of 50 consecutive light pulse potentiation (255 nm, 1.38 μW cm^−2^, 0.5 s duration, 0.5 s interval) and 50 consecutive electrical pulse depression (−97 V to +50 V, step 3 V, 0.5 s duration, 0.5 s interval). Source data are provided as a Source Data file.

Interestingly, the study finds that the device can also achieve integrated sensing, storage, and computation. Specifically, after sensing weak light signals, it enables charge storage through electrical programming and optical erasure, and ultimately realizing neuromorphic computing analogous to biological synapse functions. This provides broader application prospects for machine vision-based SBUV detectors. Figure 3a and Supplementary Fig. 16 illustrate the electrical programming/optical erasure behavior of devices at different blade coating speeds. The results indicate that under identical test conditions, only the device prepared at 0.005 mm s^−1^ exhibits distinct storage capability, with a storage window of up to 91.58 V and an electric charge storage density of 5.73 × 10^12^ cm^−2^ (Fig. 3a). It can maintain a current ratio of 10^4^ even after the retention time exceeding 50,000 s (Supplementary Fig. 17), and still retain a current ratio of 10^2^ following 4,000 s of program-read-erase-read cycling (Fig. 3b and Supplementary Fig. 18), thus demonstrating favorable charge storage capability and cycling stability. The reversible optoelectronic memory performance of the device stems from intrinsic defect-mediated charge trapping within the SiO_2_ dielectric layer. Previous literature has confirmed a variety of native defects in SiO_2_, such as oxygen vacancies and interstitial oxygen atoms^37–42^. In this work, electron paramagnetic resonance (EPR) measurements directly verify the existence of oxygen vacancy defects inside the SiO_2_ film (Supplementary Fig. 19). In addition, quantitative analysis based on the subthreshold swing extracts a high interfacial trap density of 1.2 × 10^13^ cm^−2^ eV^−1^, which solidly confirms abundant charge-trapping sites at the dielectric-semiconductor interface. For our blade-coated heterostructure device consisting of SiO_2_ and BTBTT8-syn, negative gate bias drives holes in the BTBTT8-syn channel to inject into the defect energy levels of SiO_2_ and become permanently confined. The depletion of channel carriers shifts the transfer characteristic curve toward negative voltages, corresponding to the electrical programming process. Upon illumination with 255 nm solar-blind UV light, the BTBTT8-syn active layer absorbs incident photons and generates abundant photocarriers. The resulting photoelectrons are injected into the SiO_2_ dielectric and neutralize the pre-trapped holes, which enables gradual detrapping and release of stored charges. As a result, the transfer curve recovers to its original state, accomplishing optical erasure. Relying on this distinctive reversible optoelectronic memory mechanism, we further investigate the feasibility of the device for biomimetic synaptic emulation toward biological visual information processing. In biological computing systems, synapses serve as the fundamental units responsible for information processing, which realize signal transmission via the release and reception of neurotransmitters^38–43^. Based on this, we conducted a series of synaptic behavior tests on the high-performance 0.005 mm s^−1^ device, aiming to enable machine vision to achieve biological-like visual functions (Fig. 3c). The results show that with the increase in illumination intensity, light pulse duration, and number of light pulses, the peak value of the excitatory postsynaptic current (EPSC) of the device increases gradually (Fig. 3d–f). Subsequently, the device was tested for the learning-forgetting-relearning process from short-term memory (STM) to long-term memory (LTM), and it was found that the device still exhibits excellent relearning capability (Supplementary Fig. 20). Further cyclic tests were performed with 10 consecutive cycles of long-term potentiation/long-term depression (LTP/LTD) pulses, where the continuous conductance change curve of each LTP/LTD cycle consisted of 50 consecutive light pulses for potentiation and 50 consecutive electrical pulses for depression. The results demonstrate that the device exhibits stable potentiation and depression behaviors (Fig. 3g).

### Scotopic and photopic visual adaptation in a detector array

In corona discharge monitoring systems, detectors are required to accurately identify corona signals of varying intensities even under diverse illumination conditions, which imposes higher demands on machine vision systems^44^. In contrast to artificial detectors, the distinct adaptive characteristics of photoreceptors in the human eye enable humans to perceive and recognize various objects under different illumination conditions, achieving dynamic adjustment ranging from dim‑light to bright‑light environments. Rod cells in retinal photoreceptors are responsible for detecting information in low-light conditions, while cone cells can capture visual information under high light intensities^45^. The switching between these two cell types enables the human eye’s scotopic and photopic adaptation. This regulation mechanism can be further explained by Weber’s Law^45,46^, expressed as *ΔL* = *k *× *L*, where *ΔL* denotes the perceived change, *L* represents the reference stimulus, and *k* is a fixed constant. When the background light intensity (*L*) increases (decreases), the visual threshold of the retina (*ΔL*) rises (falls), whereas retinal sensitivity (1/*ΔL*) decreases (increases). In other words, when exposed to a dim environment from a bright one, humans achieve scotopic adaptation as retinal sensitivity gradually increases, transitioning from being almost unable to see anything to perceiving objects clearly. Conversely, photopic adaptation is the reverse process of scotopic adaptation: as ambient light intensity shifts from low to high, retinal sensitivity decreases, allowing humans to gradually see objects in bright environments.

**Fig. 4:**
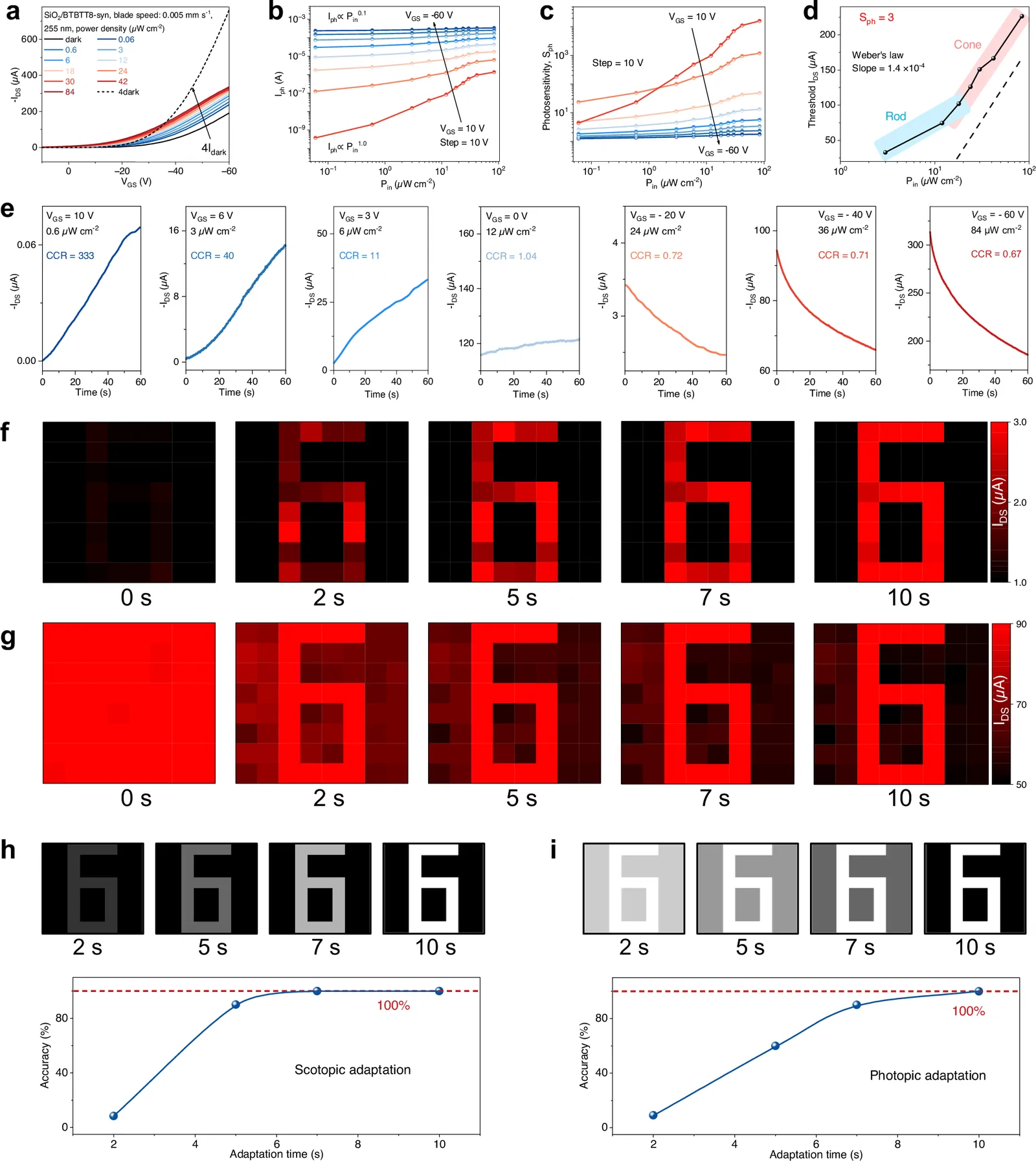
Scotopic and photopic adaptation of phototransistor array. **a** Transfer characteristic curves of the device measured at *V*_DS_ = −60 V under different incident light power densities (*P*_in_) values (255 nm wavelength). **b** Photocurrent (*I*_ph_) versus *P*_in_ for different *V*_GS_ values. **c** Photosensitivity (*S*_ph_) versus *P*_in_ for different *V*_GS_ values. **d** Threshold *I*_DS_ as a function of *P*_in_ at *S*_ph_ = 3 extracted from a). **e** Time-dependent current (*I*_DS_) of the device under different *V*_GS_ values according to different *P*_in_ values. Time courses of **f** scotopic and **g** photopic adaptation for the pattern of 6. **h** Top: the simulation schematic of scotopic adaptation by the OCR image 6. Bottom: recognition rate as a function of time for scotopic adaptation. **i** Top: the simulation schematic of photopic adaptation by the OCR image 6. Bottom: recognition rate as a function of time for photopic adaptation. Source data are provided as a Source Data file.

The application of this visual adaptation function in machine vision will greatly enhance the sensitivity and accuracy of discharge identification, enabling detectors to operate stably under varying ambient light intensities. Given the substantial practical value of this visual adaptation function, we tested the adaptive performance of the detectors under different light intensities. To begin with, a light intensity-dependent characteristic test was conducted on the devices. Figure 4a shows the transfer characteristic curves of the phototransistors under illumination with different incident light power densities (*P*_in_) ranging from 60 nW cm^−2^ to 84 µW cm^−2^ at *V*_DS_ = −60 V. The threshold voltage of the devices exhibits a positive shift with increasing light intensity. Analogous to the visual threshold of the biological retina, we defined *I*_DS_ = 4*I*_dark_ as the threshold for detectable photocurrent, where *I*_dark_ denotes the drain current under dark conditions. The current’s increase with rising *V*_GS_ is consistent with Weber’s Law. We further define the photocurrent (*I*_ph_) as *I*_ph_ = *I*_illumination_ − *I*_dark_, where *I*_illumination_ represents the *I*_DS_ under illuminated conditions. Figure 4b shows a linear positive correlation between *P*_in_ and *I*_ph_ at a positive gate bias (*V*_GS_ = 10 V). A power-law exponent of α = 1.0 indicates that the photoconductive effect dominates in this case, and the number of photogenerated carriers is nearly proportional to the incident photon flux. As the gate bias decreases, the relationship between *P*_in_ and *I*_ph_ gradually becomes sublinear (α = 0.1 at *V*_GS_ = −60 V), suggesting that the dominant mechanism is the photo-gating effect associated with trap states. By modulating the electric field distribution in the semiconductor channel, the gate voltage enables the transition from the photoconductive effect (positive gate voltage) to the photo-gating effect (negative gate voltage)^47,48^. Subsequently, we calculate the photosensitivity (*S*_ph_) using the formula *S*_ph_ = *I*_ph_/ *I*_dark_. Analysis of the functional relationship between *P*_in_ and *S*_ph_ at different gate voltages reveals that the device exhibits high *S*_ph_ characteristics similar to rod cells under positive gate voltages, whereas it shows low *S*_ph_ characteristics analogous to cone cells under negative gate voltages (Fig. 4c). To more intuitively demonstrate the adaptive characteristics of the device, we extract the gate voltage and *P*_in_ values at *S*_ph_ = 3. The semi-logarithmic model’s slope of 10 confirms the high regulation sensitivity of *P*_in_ to gate voltage, enabling rapid modulation of weak light signals (Supplementary Fig. 21). Furthermore, we extract the threshold *I*_DS_ at different *P*_in_ values when *S*_ph_ = 3, and find that its variation trend is still consistent with Weber’s Law with a high slope of 1.4 × 10^−4^ (Fig. 4d). Thus, the device can achieve a transition analogous to that between rod cells and cone cells in the biological retina by tuning *P*_in_ and gate voltage. To more comprehensively characterize the adaptive capability of the detector, we further conducted a time-dependent characteristic test on the device. Different gate voltages and *P*_in_ correspond to distinct excitation or suppression characteristic curves. To quantitatively compare the extent of current excitation or suppression effects, we define the current change ratio (CCR) of the device under different gate voltages as follows:

1 $${{\rm{CCR}}}=\frac{{I}_{{{\rm{DS}}}\mbox{-}60{{\rm{s}}}}}{{I}_{{{\rm{DS}}}\mbox{-}0{{\rm{s}}}}},$$

where *I*_DS-0s_ denotes the initial drain current value, and *I*_DS-60s_ represents the drain current value at 60 s. Under weak light illumination, the application of positive gate voltage induces excitatory characteristics, where *I*_DS_ increases with time. The CCR values are 333, 40 and 11 under low light intensities of 0.6 µW cm^−2^ (*V*_GS_ = 10 V), 3 µW cm^−2^ (*V*_GS_ = 6 V) and 6 µW cm^−2^ (*V*_GS_ = 3 V), respectively. In contrast, under strong light illumination, the application of negative gate voltage results in inhibitory characteristics, where *I*_DS_ decreases with time. The CCR values are 0.72, 0.71 and 0.67 under high light intensities of 24 µW cm^−2^ (*V*_GS_ = −20 V), 42 µW cm^−2^ (*V*_GS_ = −40 V), and 84 µW cm^−2^ (*V*_GS_ = −60 V), respectively (Fig. 4e).

Based on the light-intensity-dependent and time-dependent characteristics of the phototransistors, we simulate the scotopic and photopic adaptation processes of the human retina by utilizing an 8 × 8 transistor array to sense the pattern 6. For the array’s scotopic adaptation test, we apply a gate voltage of 6 V to all devices. Among them, the 20 devices corresponding to the pattern 6 are exposed to weak light at 6 µW cm^−2^, while the remaining 44 devices are subjected to dim background illumination at 3 µW cm^−2^. According to the *I*_DS_ values of different devices (Supplementary Figs. 22 and 23 show the detailed test methods and results), the digit 6 perceived at different time points under dim light conditions is visualized (Fig. 4f). To quantitatively evaluate the image quality, we calculate the image contrast (*C*) based on the gray level (*G*) difference between the two kinds of pixels inside and outside 6 according to the following equation:

2 $$C={{G}^{*}}_{{\rm{illumination}}}-{{G}^{*}}_{{\rm{dark}}},$$

where ${G\,}_{{\rm{illumination}}}^{*}$ denotes the average *G* value of the 20 devices under light illumination, and ${G\,}_{{\rm{dark}}}^{*}$ represents the average *G* value of the 44 background devices under dim conditions. To maintain neural stability within the biological visual system, the output of ganglion cells actually remains within a fixed range even when light intensity varies over a broad spectrum. Therefore, to ensure consistent visual perception across the field of view, we extract the dynamic changes in each pixel over a 10-s period and define the gray level of 0 corresponding to *I*_DS_ = 1 μA and the gray level of 255 corresponding to *I*_DS_ = 3 μA. The pattern of 6 cannot be recognized at the beginning with zero contrast because of the low photocurrent under a relatively low light illumination. With the visual adaptation effect, the image contrast gradually increases to 136 (2 s), 227 (7 s) and 249 (10 s), respectively. This enhancement is analogous to the scotopic adaptation of the retina. Similarly, in the photopic adaptation test, a gate voltage of −40 V is applied to all devices: the 20 devices corresponding to the pattern 6 are exposed to strong light at 84 μW cm^−2^, while the other 44 devices are subjected to bright background illumination at 36 μW cm^−2^. Figure 4g shows the pattern 6 perceived at different time points under strong light, which is extracted from the *I*_DS_ data (Supplementary Fig. 24). Based on this test, we define the gray level of 0 corresponding to *I*_DS_ = 50 μA and the gray level of 255 corresponding to *I*_DS_ = 90 μA. The 20 devices under strong light illumination exhibit a constant *G* value of 255 due to saturation, and the pattern contrast increases sequentially to 129 (2 s), 199 (7 s), and 221 (10 s). The digit 6 gradually transforms from an initially dazzling and indistinguishable pattern into a visually comfortable image. This photopic adaptation phenomenon, analogous to that of the retina, further confirms that the device maintains the ability to regulate image brightness even under strong light conditions. To quantitatively evaluate the image recognition capability of visual adaptation under different conditions, we employ an Optical Character Recognition (OCR) model to identify digital images under dim and bright backgrounds, respectively. The top images in Fig. 4h, i show the schematics of images processed by normalization (grayscaling, binarization, denoising, etc.) during scotopic and photopic adaptation, respectively, where the image contrast increases with time. The recognition rate under scotopic adaptation increases from 8.3% at 2 s to 100% at 10 s (Fig. 4h), while that under photopic adaptation rises from 9.1% at 2 s to 100% at 10 s (Fig. 4i). Benefiting from the excellent visual adaptive capability of the device, the images achieve high recognition accuracy, which lays a solid foundation for machine vision to realize stable and accurate target recognition in complex practical scenarios. When applied to high-voltage discharge detection, the device enables precise deep-ultraviolet detection of discharges both in dim nights and bright days. It exhibits favorable environmental adaptability and anti-interference capability for detection scenarios under varying background light conditions, thereby providing reliable technical support for the real-time monitoring of discharge faults in high-voltage equipment under complex operating conditions.

### Classification and warning for high voltage discharge

**Fig. 5:**
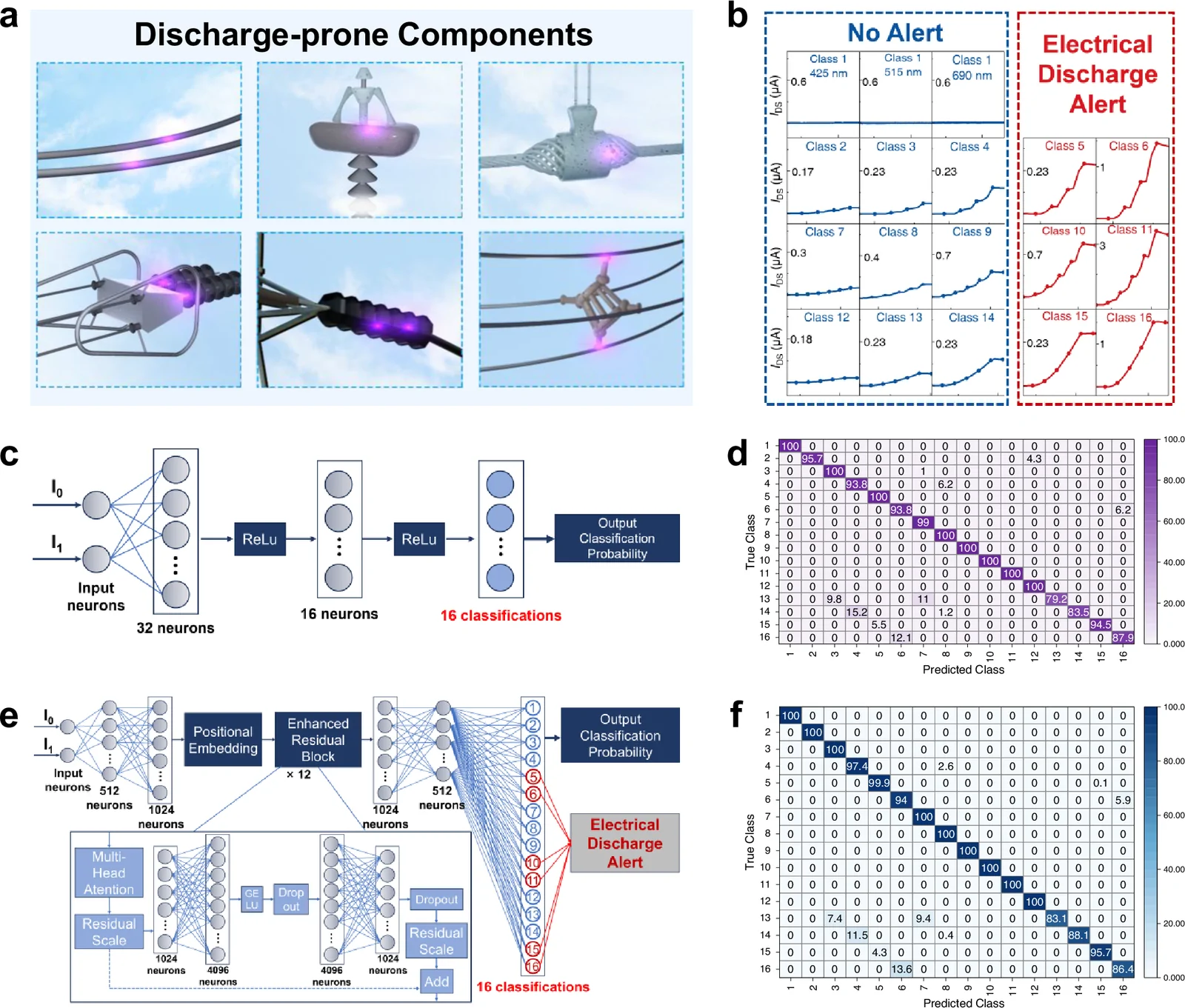
High-voltage discharge detection system for corona discharges early warning. **a** Schematic illustration of discharge detection and main discharge components. **b** Actual current signals corresponding to classification 16. **c** Schematic illustration of the simple architecture network model. **d** The confusion matrix comparing the predicted classification and the actual classification after the simulation calculation of the simple model. **e** A schematic illustration of the Attention-Augmented MLP model system architecture. **f** The confusion matrix of the predicted classification and the actual classification obtained through simulation and calculation by the Attention-Augmented MLP model. Source data are provided as a Source Data file.

To verify the practical application feasibility of organic SBUV detectors in high-voltage discharge monitoring scenarios, we constructed a corresponding theoretical analysis model and, based on this model, achieved accurate classification and early warning of discharge detection signals. There are six main types of discharge-prone components in high-voltage transmission equipment: conductors, grading rings, suspension clamps, shielding rings, insulators, and spacer rods (Fig. 5a)^49^. In addition, based on the time-domain characteristics of discharge behaviors, the typical forms of corona discharge can be classified into two types: intermittent discharge and continuous discharge^50^. Through the photoelectric conversion effect, the organic SBUV detector can convert the corona discharge radiation signals in the deep-ultraviolet band into collectible current signals. Therefore, we classified the detection signals into 16 categories by integrating multiple influencing factors, including discharge forms, corona deep-ultraviolet light intensity, and background light interference (Supplementary Fig. 25). We established a complete experimental dataset based on real-time electrical signals collected from the device, covering sixteen distinct types of characteristic corona discharge waveforms. The sensor generates nearly negligible photocurrent under visible illumination at 425 nm, 515 nm and 690 nm, which corroborates its favorable anti-interference capability and precise 255 nm selective detection performance. The collected discharge signals fall into three representative categories: short-interval pulse discharges containing three consecutive pulses (Categories 2–6), long-interval pulse discharges with five sequential pulses (Categories 7–11), and continuous 500 ms steady discharges (Categories 12–16). Corresponding excitation light intensities for these discharge modes are 0.06, 0.19, 1.4, 2.3 and 4.5 µW cm^−2^, respectively. The device output photocurrent increases monotonically with rising incident irradiance (Fig. 5b), providing distinguishable feature information for subsequent classification tasks.

To verify the inherent signal preprocessing superiority of the neuromorphic sensor, we developed a compact three-layer fully connected perceptron as the core lightweight classification framework, whose architecture is displayed in Fig. 5c. This model adopts a 2-neuron input layer to receive two-dimensional signal features captured directly by the sensor. Free of self-attention and residual structures, it realizes efficient feature transformation via two activated fully connected layers and completes the classification of sixteen discharge signal patterns. We partitioned the dataset by allocating the initial 6000 samples of each category for model training and the rest for testing. After sufficient iterative optimization, the single-category classification accuracy stabilizes above 80% (Fig. 5d and Supplementary Fig. 26a), and the overall average classification accuracy reaches 95.5% (Supplementary Fig. 26b). These results fully demonstrate that the proposed sensor can independently extract faint corona discharge signatures and suppress environmental noise, thereby exhibiting prominent native signal preprocessing efficiency for discharge monitoring. On this basis, we further constructed an attention-augmented multi-layer perceptron (Attention-Augmented MLP) integrated with self-attention and residual modules for practical high-voltage discharge early-warning applications; its detailed network configuration is illustrated in Fig. 5e. This complex network only serves as an engineering auxiliary scheme to improve system stability, while the high classification accuracy of the lightweight perceptron confirms that reliable discharge identification fundamentally relies on the superior signal output quality of our neuromorphic sensor itself. This model achieves high-dimensional mapping of two-dimensional coordinate features and enhancement of spatial discriminability via double-layer linear transformation and learnable positional encoding. Then, it stacks 12 enhanced residual blocks containing multi-head self-attention and expansion-compression feed-forward networks to extract deep features. Finally, it outputs the classification probabilities of 16 categories through normalization, dimensionality reduction, and linear layers. This model uses the same dataset as the simple model mentioned above. During the training process, the classification accuracy reaches a relatively high level of over 80% after 91 epochs (Fig. 5f and Supplementary Fig. 27a), with an average classification accuracy of 96.5% (Supplementary Fig. 27b), fully verifying the excellent deep feature learning ability of the network. On the basis of accurate classification, light intensities of 2.3 µW cm^−2^ and 4.5 µW cm^−2^ are set as early warning signals. Thus, the system automatically outputs alarm signals when the data are classified into categories 5, 6, 10, 11, 15, and 16 (Supplementary Fig. 28), indicating that the corona discharge is relatively severe and maintenance is required.

## Discussion

We report an ultra-high-sensitivity organic solar-blind ultraviolet (SBUV) photodetector that enables reliable weak-light detection at an ultra-low illumination intensity of 60 nW cm^−2^, significantly outperforming many conventional SBUV devices in low-light sensing performance with superior photosensitivity, photoresponsivity and specific detectivity. Unlike traditional counterparts that merely achieve simple signal transduction without data processing capacity, this device innovatively integrates excellent optical properties with on-chip information storage and in-situ processing capabilities. Moreover, its intrinsic scotopic-photopic adaptation ensures stable dynamic responses under complex, fluctuating illumination conditions commonly encountered in practical industrial applications. Systematic simulations mimicking actual high-voltage corona discharge scenarios further confirm its robust anti-interference ability against ambient background noise and precise signal classification for early warning of incipient faults. This work establishes solid experimental and theoretical foundations for the practical deployment of organic SBUV detectors in corona discharge monitoring, with significant implications for advancing low-cost, flexible, and intelligent monitoring technologies in power systems and smart grids.

## Methods

### Materials

2-(5-octylthiophen-2-yl) [1] benzothieno[3,2-b][1] benzothiophene (BTBTT8-syn) was synthesized in the laboratory of the Changchun Institute of Applied Chemistry. Chlorobenzene (99.5%) was purchased from Meryer.

### Substrate preparation

One-sided polished P-type silicon substrates were ultrasonically cleaned in deionized water, acetone and isopropanol, respectively, and then dried with nitrogen, and then treated with 50 W O_2_ plasma for 5 min (plasma treatment was carried out using NAEN TECH).

### Solution shearing process

BTBTT8-syn was dissolved in chlorobenzene, with the mixed solution concentration being 2 mg mL^−1^. The blade was fixed by an air pump, and the substrate was adsorbed on the substrate at a temperature of 40 °C. The angle between the blade and the substrate was 15°, and the width of the slit between the blade and the base was 100 µm. 2.5 µL of the solution was injected and coated at constant shear rates of 0.005, 0.01, 0.05 and 0.1 mm s^−1^. The coating equipment was prepared by Peking University Yangtze Delta Institute of Optoelectronics.

### Device fabrication and characterization

A metal mask was used to deposit 20 nm Au as the source/drain on the surface of BTBTT8-syn. The electrical properties of the devices were tested by a Keithley 2636B System in an air environment, and the mobility was calculated by using the saturation zone equation [*I*_DS_ = *µ C*_i_ (*W*/2 *L*) (*V*_GS_–*V*_th_)^2^]. The channel *W* of our device is 285 µm, and *L* is 25 µm. The phototransistors and the photonic synapses were characterized in the air by using a Keithley 2636B semiconductor parameter analyzer and were illuminated with the assistance of a deep ultraviolet lamp with a wavelength of 255 nm. UV-vis-NIR absorption spectra for BTBTT8-syn thin films on quartz substrates were collected using a SHIMADZU UV-3600 Plus spectrometer. POM and OM images were taken under a Nikon Eclipse Ci-POL polarized microscope. Use Thermo Scientific Talos F200X to take TEM images. The semiconductor crystal structure was analyzed by one-dimensional (1D)-XRD (MiniFlex600) with 2°–30°scanning and 2D-GIWAXS from the University of Chinese Academy of Sciences. Steady-state PL was measured by the FLS1000 spectrophotometer. The PL spectra of BTBTT8-syn films deposited on different interfaces at room temperature and variable temperatures were in the range of 370–700 nm. The low-temperature test used a Lakeshore low-temperature probe station equipped with a Keithley 2636 meter, and we performed a variable temperature test in a vacuum of 3 × 10^−4^ Pa.

## Supplementary information

Supplementary Information

Transparent Peer Review file

## Source data

Source Data

## Data Availability

All source data generated in this study and underlying the figures/charts are provided in the Source Data files. The raw data for the main figures are available in Figshare (DOI: 10.6084/m9.figshare.33228459) under CC BY 4.0. Source data are provided with this paper.

## Code availability

The code that supports the findings of this study has been deposited in Figshare under the DOI: 10.6084/m9.figshare.33228459 (https://doi.org/10.6084/m9.figshare.33228459) and is freely accessible under the Creative Commons Attribution 4.0 International (CC BY 4.0) license. The ARTICLE IN PRESS repository contains all raw data underlying the main figures and the code used for neural network simulations.
