# Supplementary material for: Ultrahigh-sensitivity adaptive solar-blind organic neuromorphic sensors for robust corona discharge monitoring
Source: Nat Commun. 2026 Sep 4;17:10510. doi: 10.1038/s41467-026-77523-y (PMC13639085; doi:10.1038/s41467-026-77523-y)
Supplement: Supplementary file 1 [file 41467_2026_77523_MOESM1_ESM.pdf]

Supporting Information

Ultrahigh-Sensitivity Adaptive Solar-Blind Organic Neuromorphic Sensors for Robust Corona Discharge Monitoring

Yang Liu[1], Yulin Sha[2], Xiaonan Ma[2], Hongkun Tian[3], Deyang Ji[1,4*], Wenping Hu[5]

[1]Institute of Molecular Aggregation Science, Tianjin University, Tianjin 300072, China.

[2]Institute of Molecular Plus, Tianjin University, Tianjin 300072, China.

[3]State Key Laboratory of Polymer Physics and Chemistry, Changchun Institute of Applied Chemistry, Chinese Academy of Sciences, Changchun 130022, China

[4]Key Laboratory of Organic Integrated Circuit, Ministry of Education, Tianjin University, Tianjin 300072, China.

[5]State Key Laboratory of Advanced Materials for Intelligent Sensing, Tianjin University, Tianjin 300072, China.

Corresponding author. E-mail address: jideyang@tju.edu.cn (D.J.)

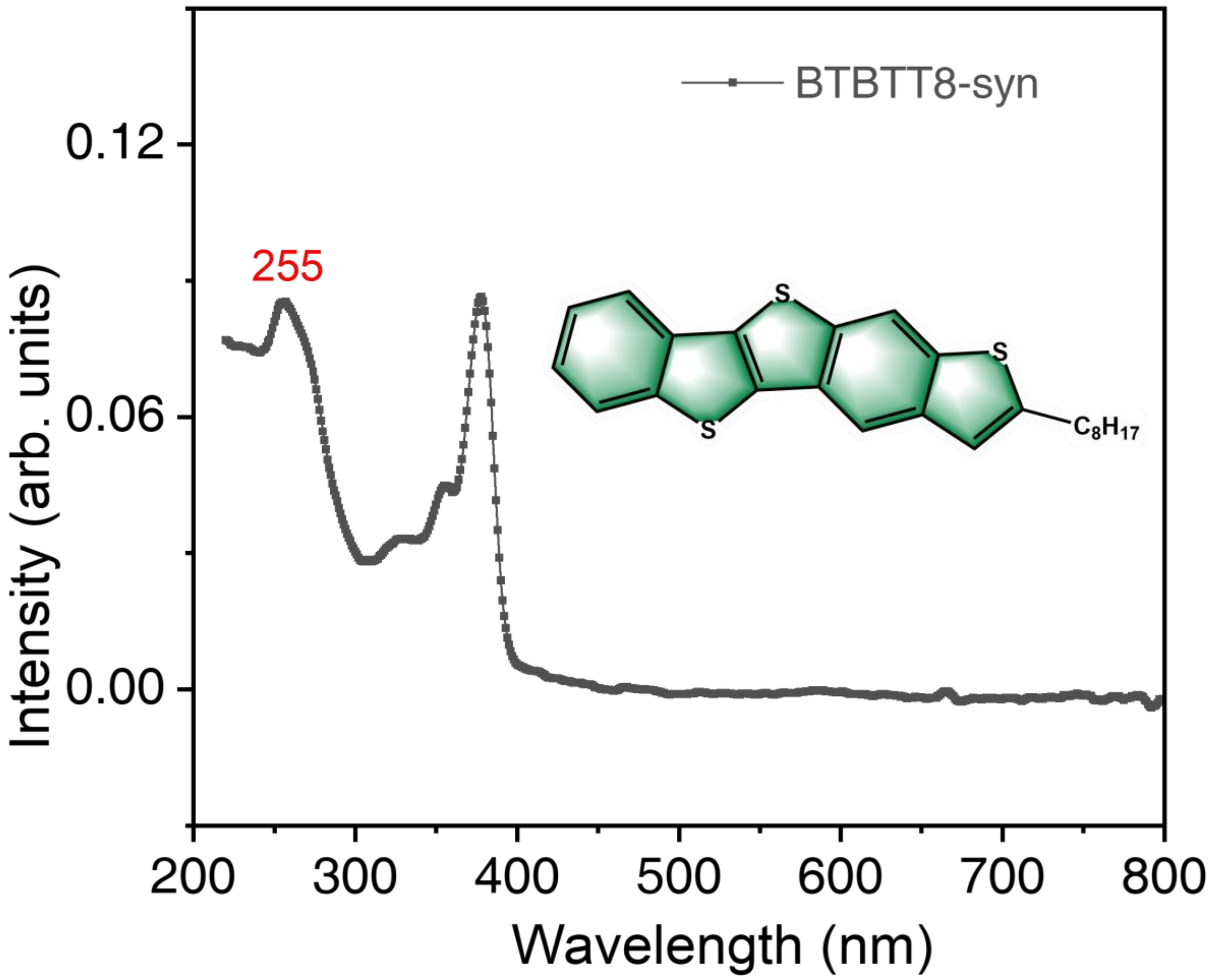

Supplementary Fig. 1. The structural formula and ultraviolet-visible (UV-Vis) absorption spectrum of BTBTT8-syn. The test wavelength range of the UV-Vis absorption spectrum is 220–800 nm, with absorption signals detected in the wavelength region below 400 nm. When targeting the solar-blind region (200–280 nm), the BTBTT8-syn film exhibits its strongest absorption peak at 255 nm. Source data are provided as a Source Data file.

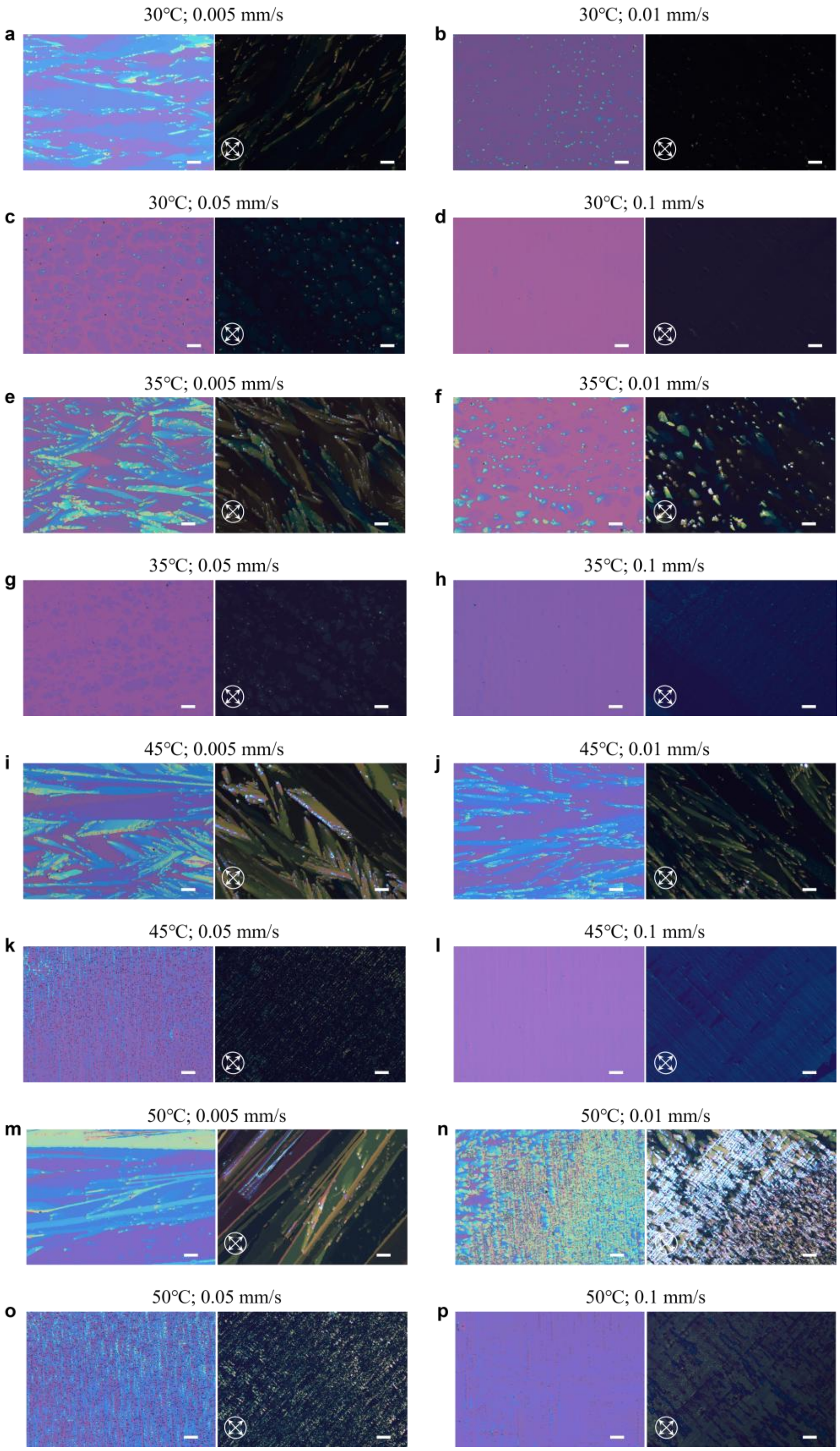

Supplementary Fig. 2. Optical microscopy (OM, left) and polarized optical microscopy (POM, right: 45°) images of the BTBTT8-syn film prepared by the solution shearing technique. The substrate temperatures are (**a-d**) 30 °C, (**e-h**) 35 °C, (**i-l**) 45 °C and (**m-p**) 50 °C. All scale bars are 100 μm.

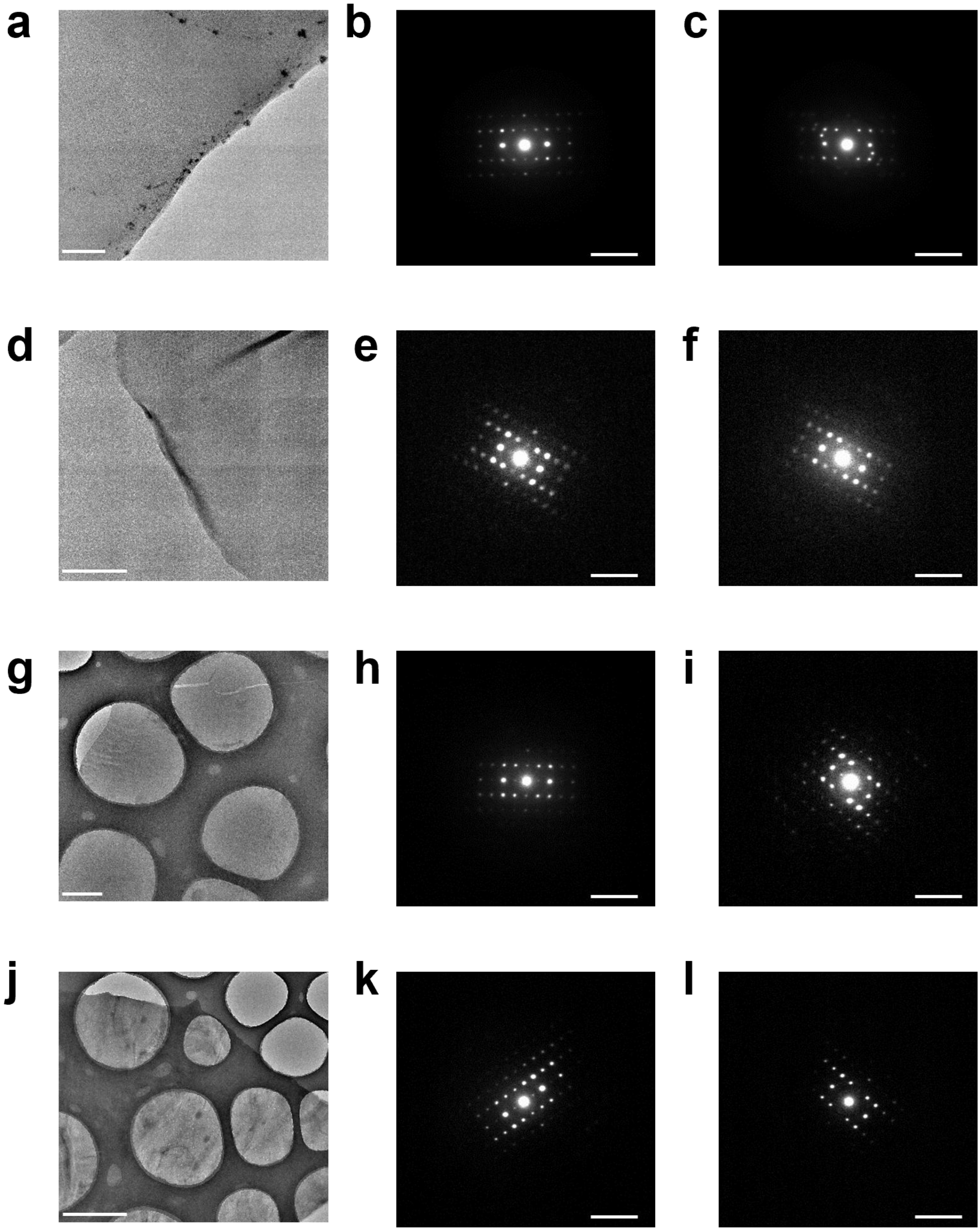

Supplementary Fig. 3. Transmission electron microscopy (TEM) and selected area electron diffraction (SAED) images of thin films prepared at different blade-coating speeds. The blade-coating speeds are (**a-c**) 0.005 mm s^{-1}, (**d-f**) 0.01 mm s^{-1}, (**g-i**) 0.05 mm s^{-1} and (**j-l**) 0.1 mm s^{-1}, respectively. Among these, a, d, g and j correspond to TEM images, while the rest are SAED images. Scale bars: a, 100 nm; d, 500 nm; g, 1 µm; j, 2 µm. Scale bars for all SAED patterns, 5.00 nm^{-1}.

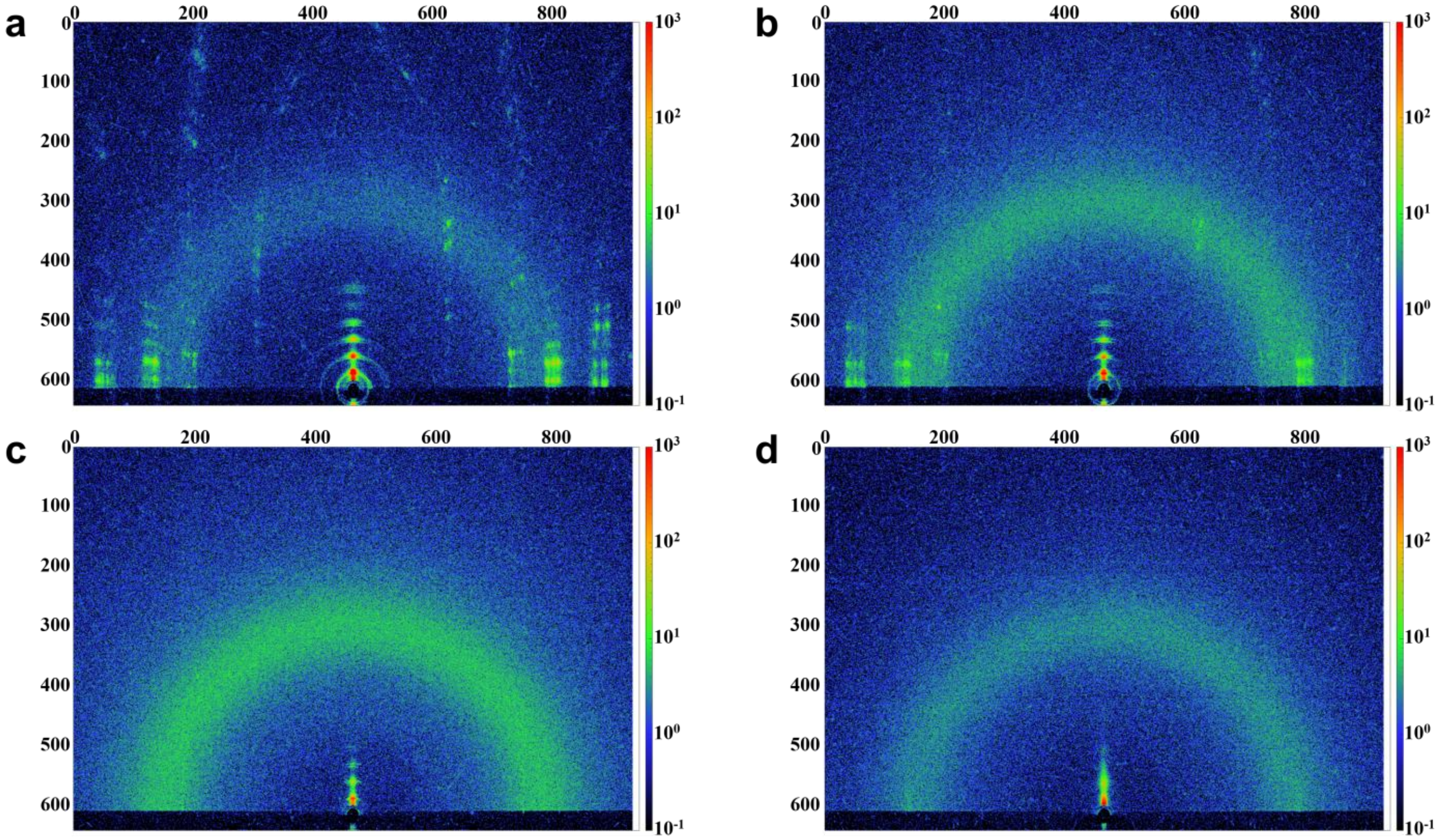

Supplementary Fig. 4. Grazing incidence wide-angle X-ray scattering (GIWAXS) characterization images of the thin films. The blade-coating speeds are **a,** 0.005 mm s^{-1}, **b,** 0.01 mm s^{-1}, **c,** 0.05 mm s^{-1} and **d,** 0.1 mm s^{-1}.

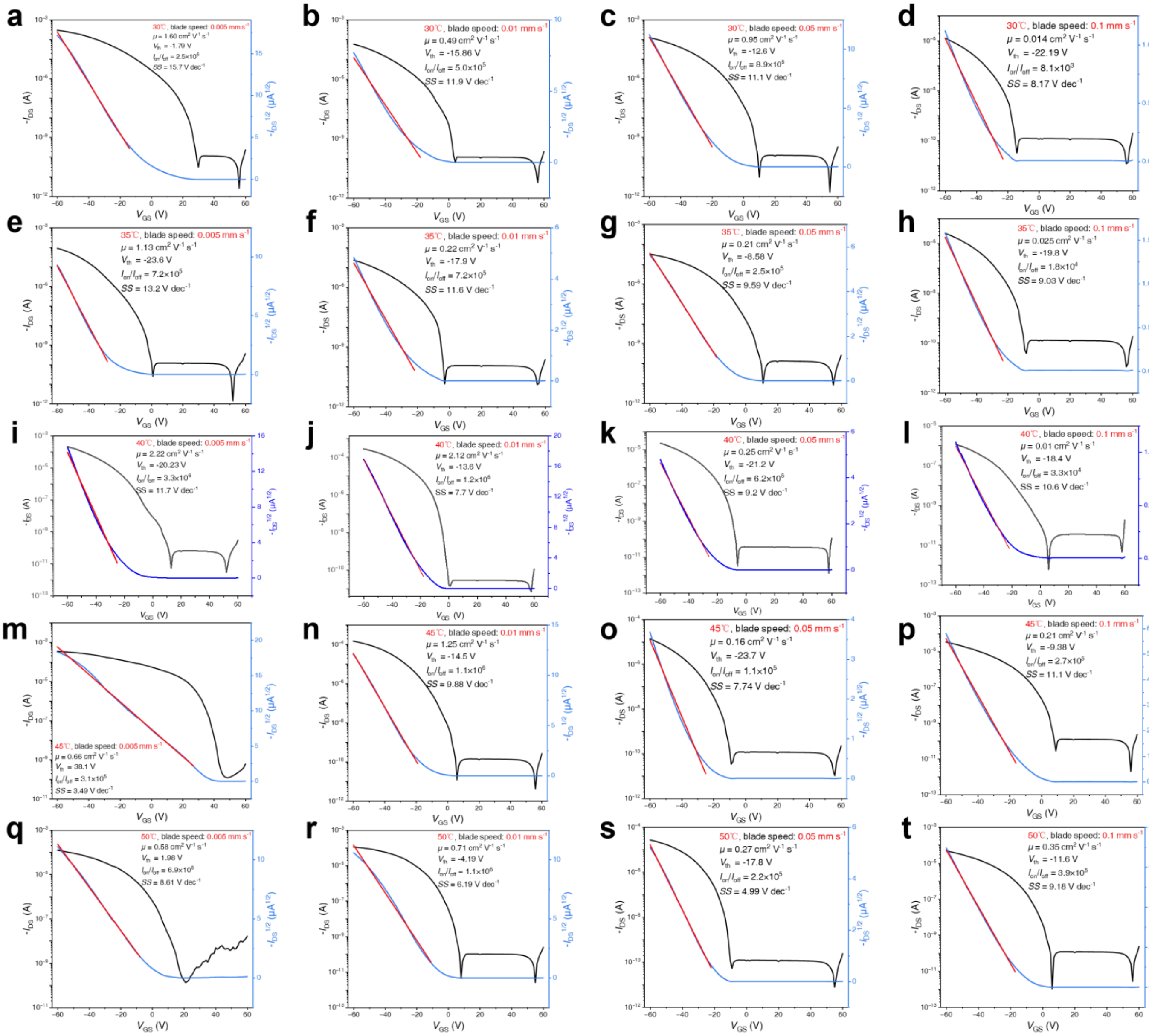

Supplementary Fig. 5. Transfer curves of devices fabricated at different substrate temperatures and blade-coating speeds for semiconductors. Transfer curves of devices fabricated at substrate temperatures of (**a-d**) 30 °C, (**e-h**) 35 °C, (**i-l**) 40 °C, (**m-p**) 45 °C and (**q-t**) 50 °C. μ denotes the mobility, V_{th} denotes the threshold voltage, I_{on}/I_{off} denotes the current on/off ratio, and SS denotes the subthreshold swing. Source data are provided as a Source Data file.

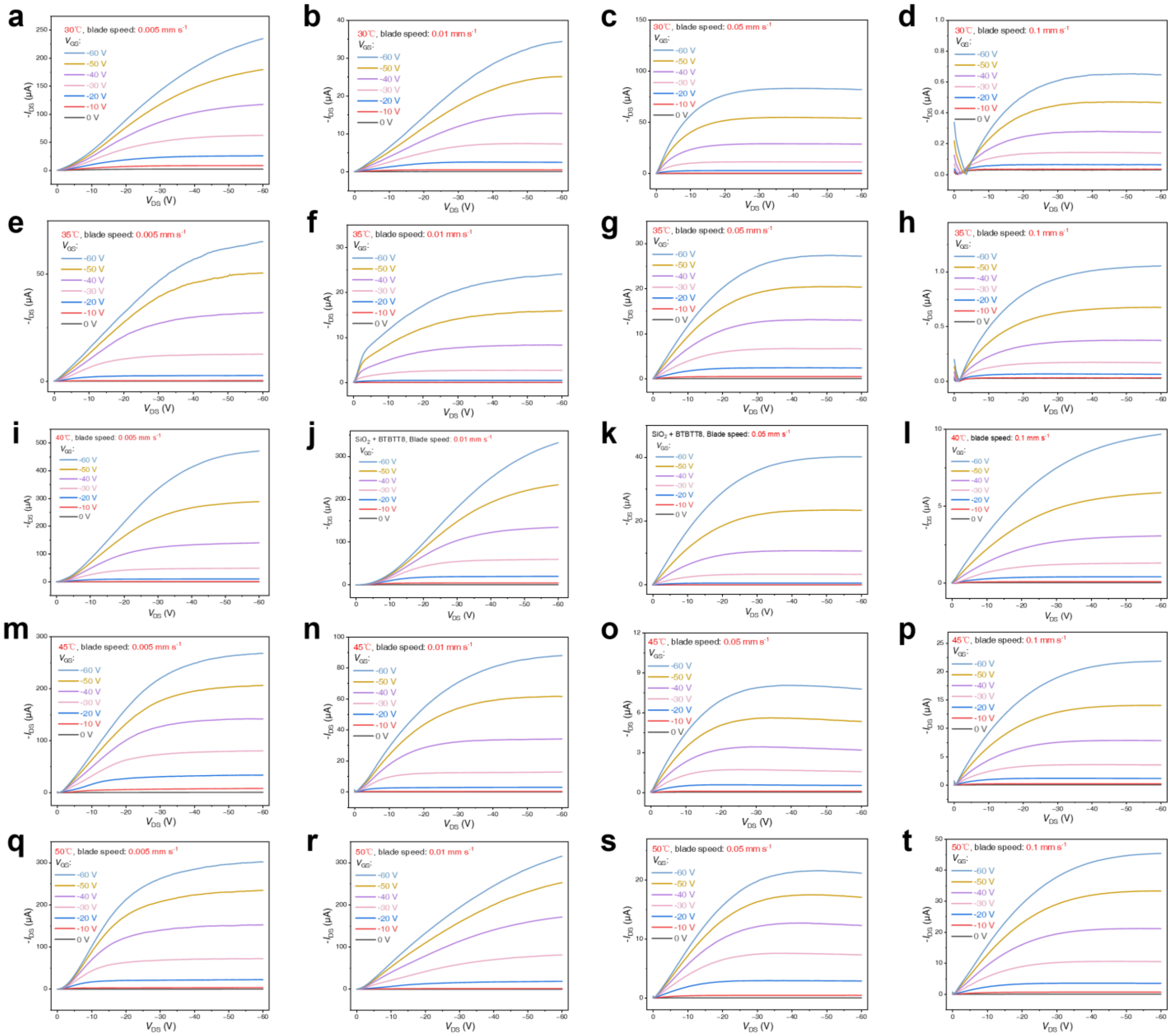

Supplementary Fig. 6. Output curves of devices fabricated at different substrate temperatures and blade-coating speeds for semiconductors. Output curves of devices fabricated at substrate temperatures of (**a-d**) 30 °C, (**e-h**) 35 °C, (**i-l**) 40 °C, (**m-p**) 45 °C and (**q-t**) 50 °C. Source data are provided as a Source Data file.

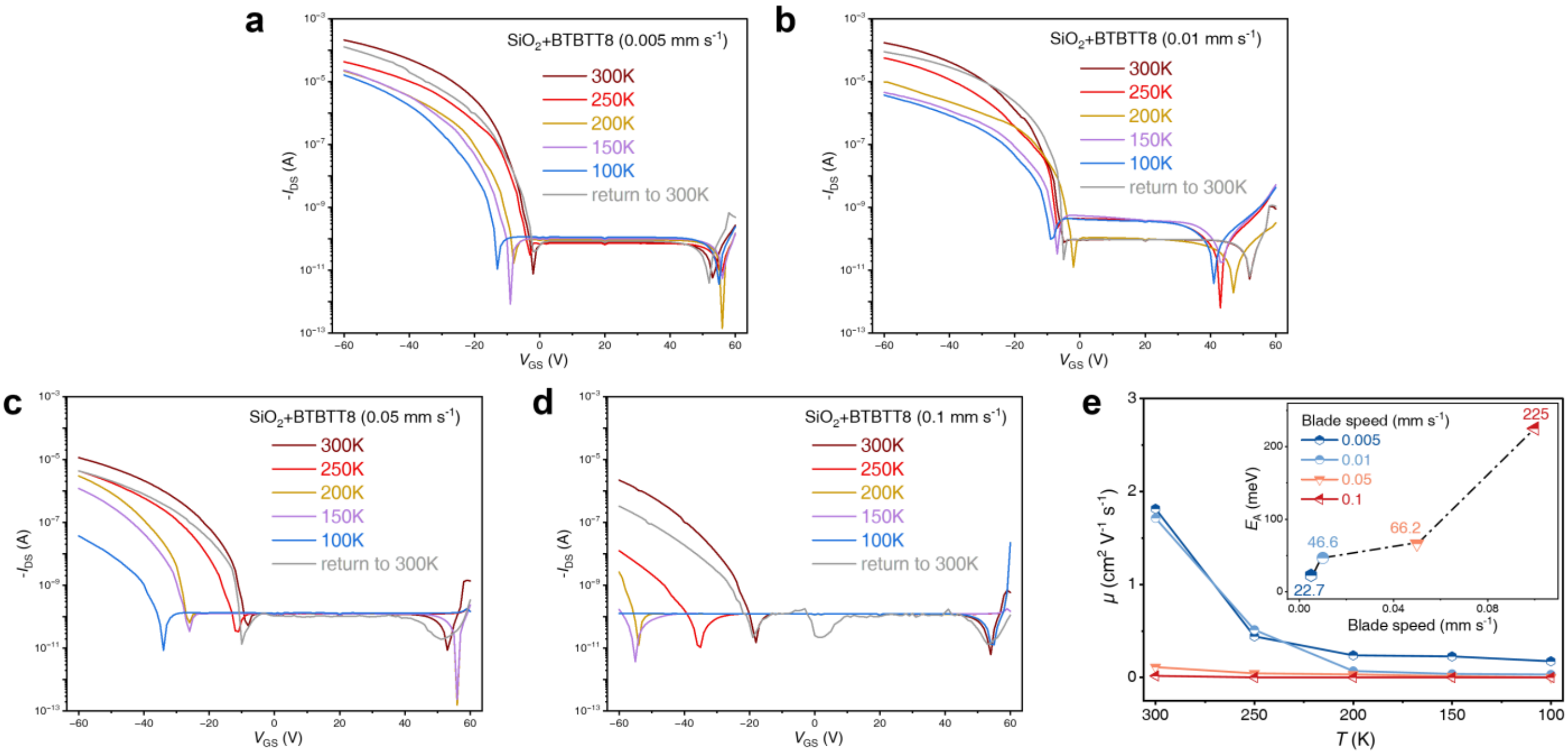

Supplementary Fig. 7. The transfer curves of different devices with temperature changes. The blade-coating speeds of the device's semiconductor layer are as follows: **a,** 0.005 mm s^{-1}, **b,** 0.01 mm s^{-1}, **c,** 0.05 mm s^{-1} and **d,** 0.1 mm s^{-1}. **e,** Statistics of temperature-dependent mobility and activation energy (E_A) for devices prepared at different blade-coating speeds. Source data are provided as a Source Data file.

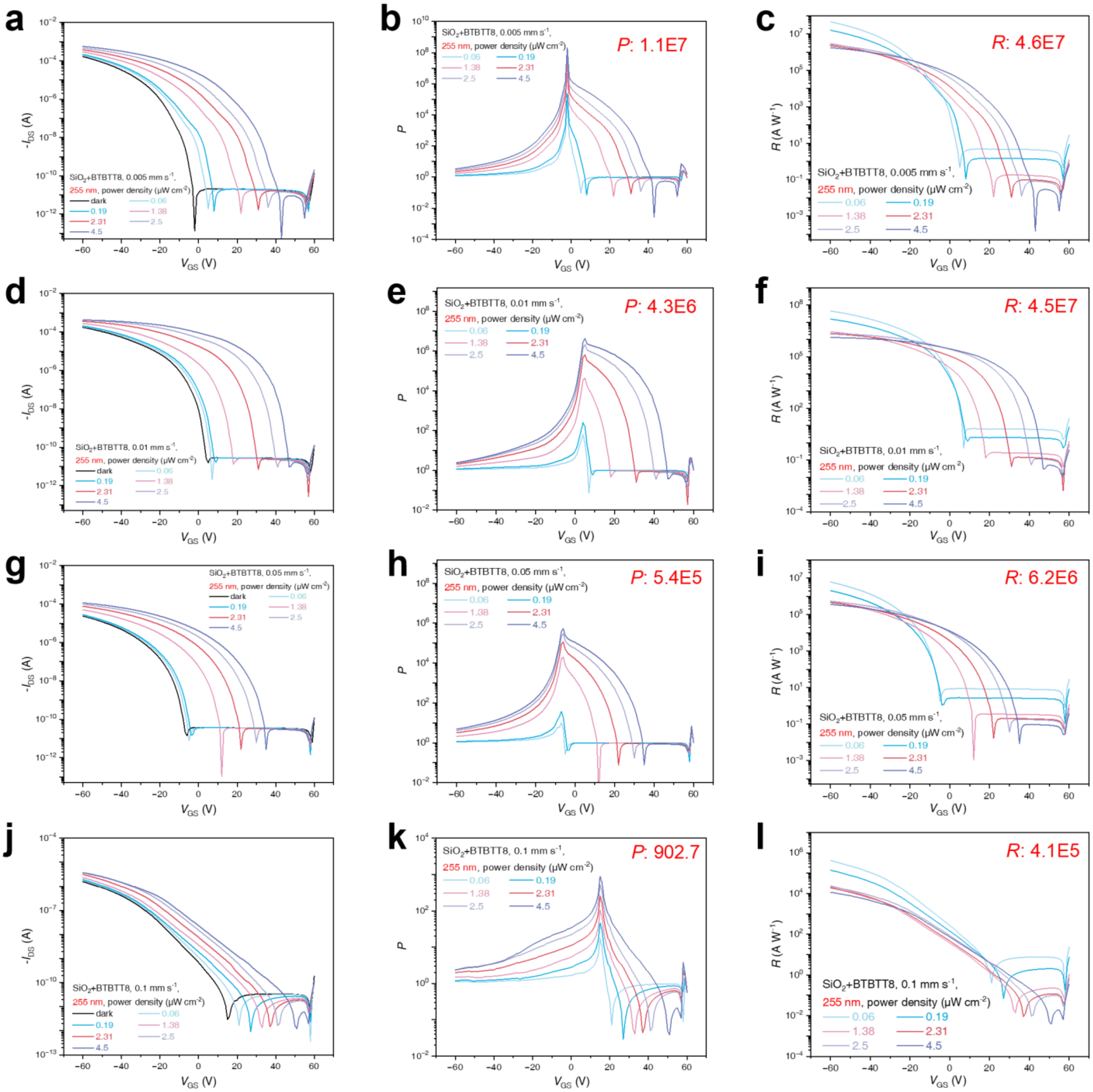

Supplementary Fig. 8. Light response curves and photosensitivity (*P*), photoresponsivity (*R*) curves of each device. The blade-coating speeds of the device's semiconductor layer are as follows: (**a-c**) 0.005 mm s^{-1}, (**d-f**) 0.01 mm s^{-1}, (**g-i**) 0.05 mm s^{-1} and (**j-l**) 0.1 mm s^{-1}. Source data are provided as a Source Data file.

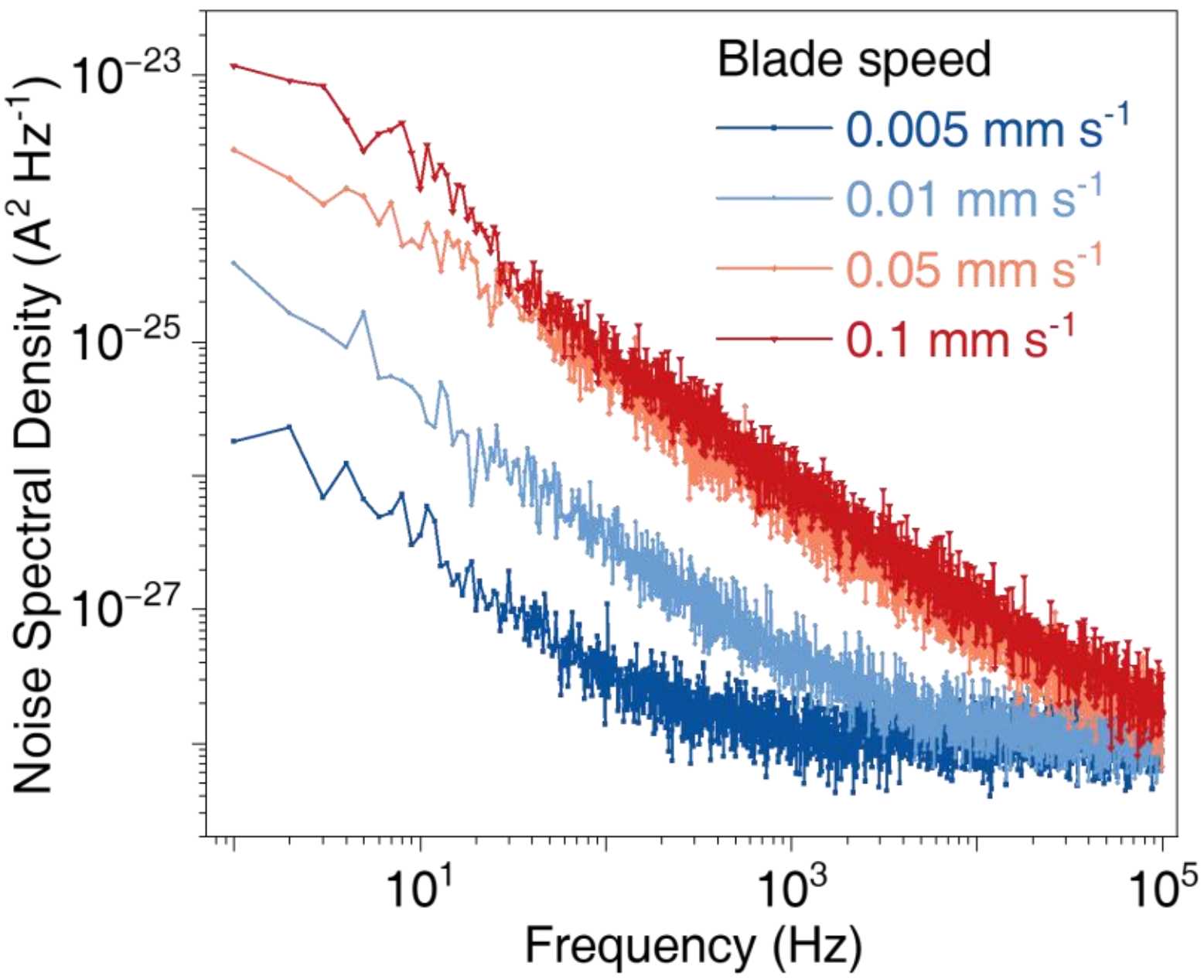

Supplementary Fig. 9. Noise current curves at different blade-coating speeds. Source data are provided as a Source Data file.

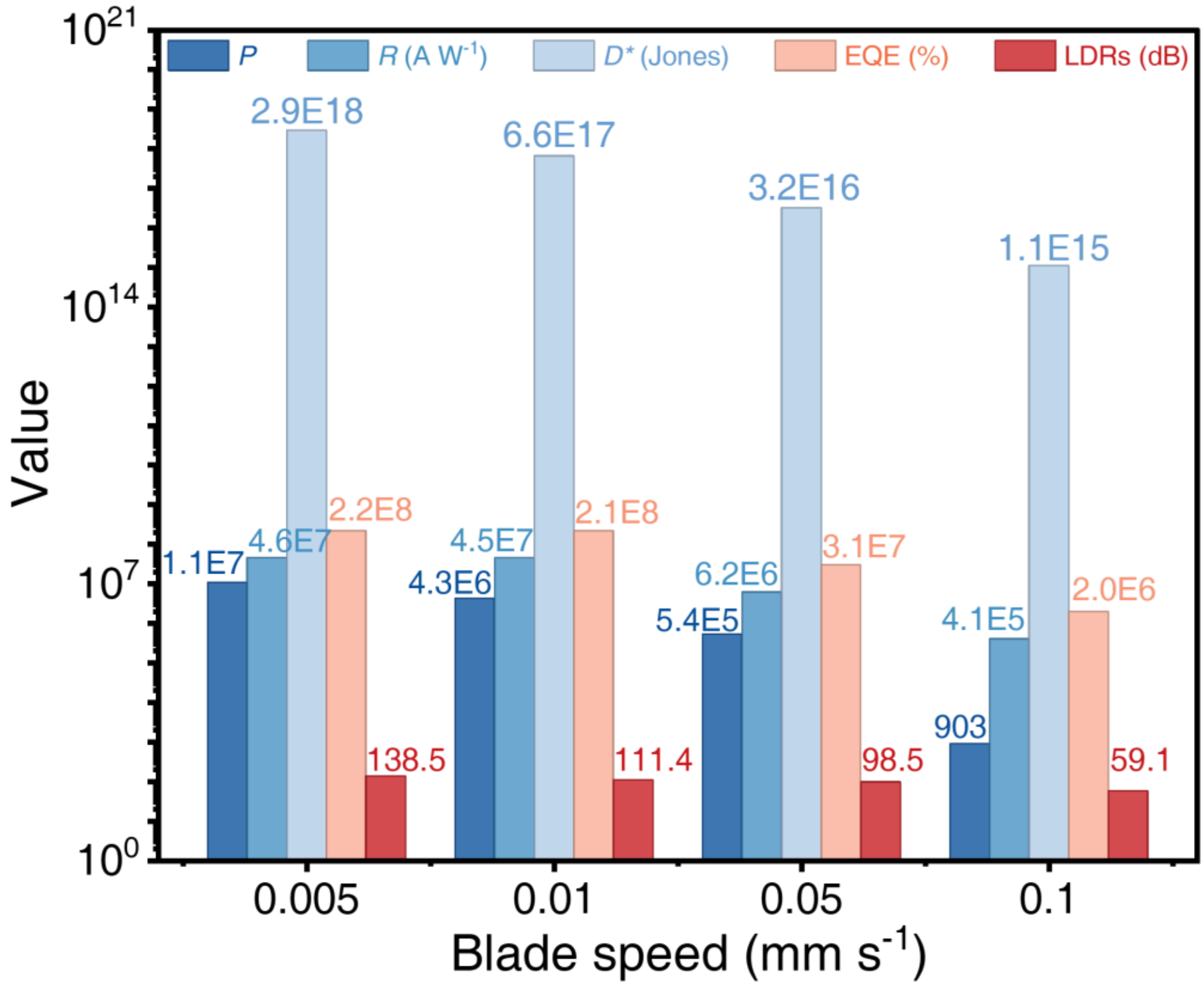

Supplementary Fig. 10. Statistical comparison of optical parameters of devices with different blade-coating speeds. Photosensitivity (*P*), photoresponsivity (*R*), specific detectivity (D^*), external quantum efficiency (EQE) and linear dynamic ranges (LDRs) statistics of devices prepared at different blade-coating speeds. Source data are provided as a Source Data file.

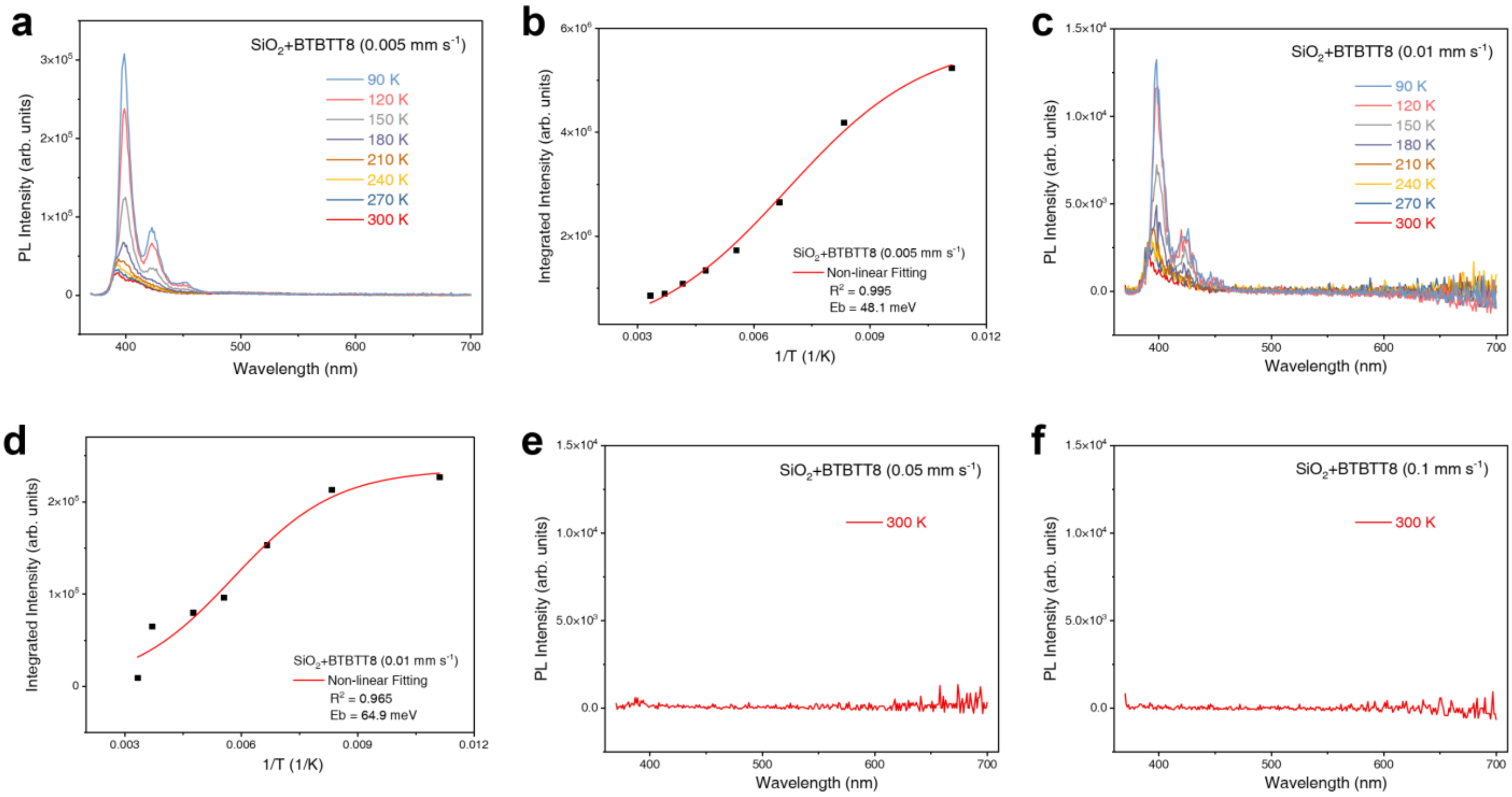

Supplementary Fig. 11. The temperature-dependent photoluminescence (PL) spectrograms and the calculated exciton binding energy (Eb) values of semiconductor layers. The blade-coating speeds are as follows: (**a** and **b**) 0.005 mm s^{-1}, (**c** and **d**) 0.01 mm s^{-1}, **e,** 0.05 mm s^{-1} and **f,** 0.1 mm s^{-1}. Source data are provided as a Source Data file.

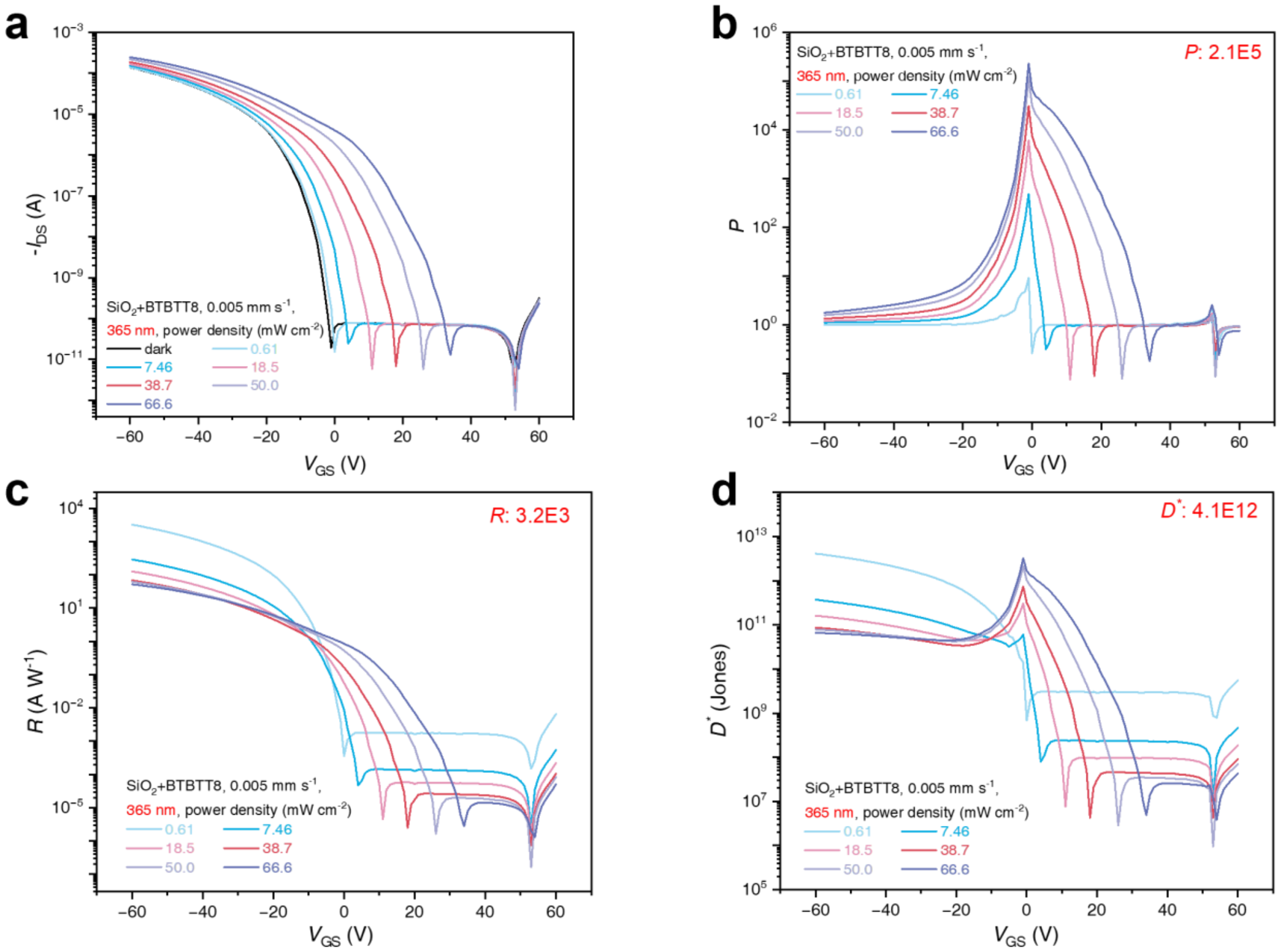

Supplementary Fig. 12. Spectral selectivity of devices. a, The light response curve and corresponding **b,** *P*, **c,** *R*, **d,** D^* curves of the device at 365nm. Source data are provided as a Source Data file.

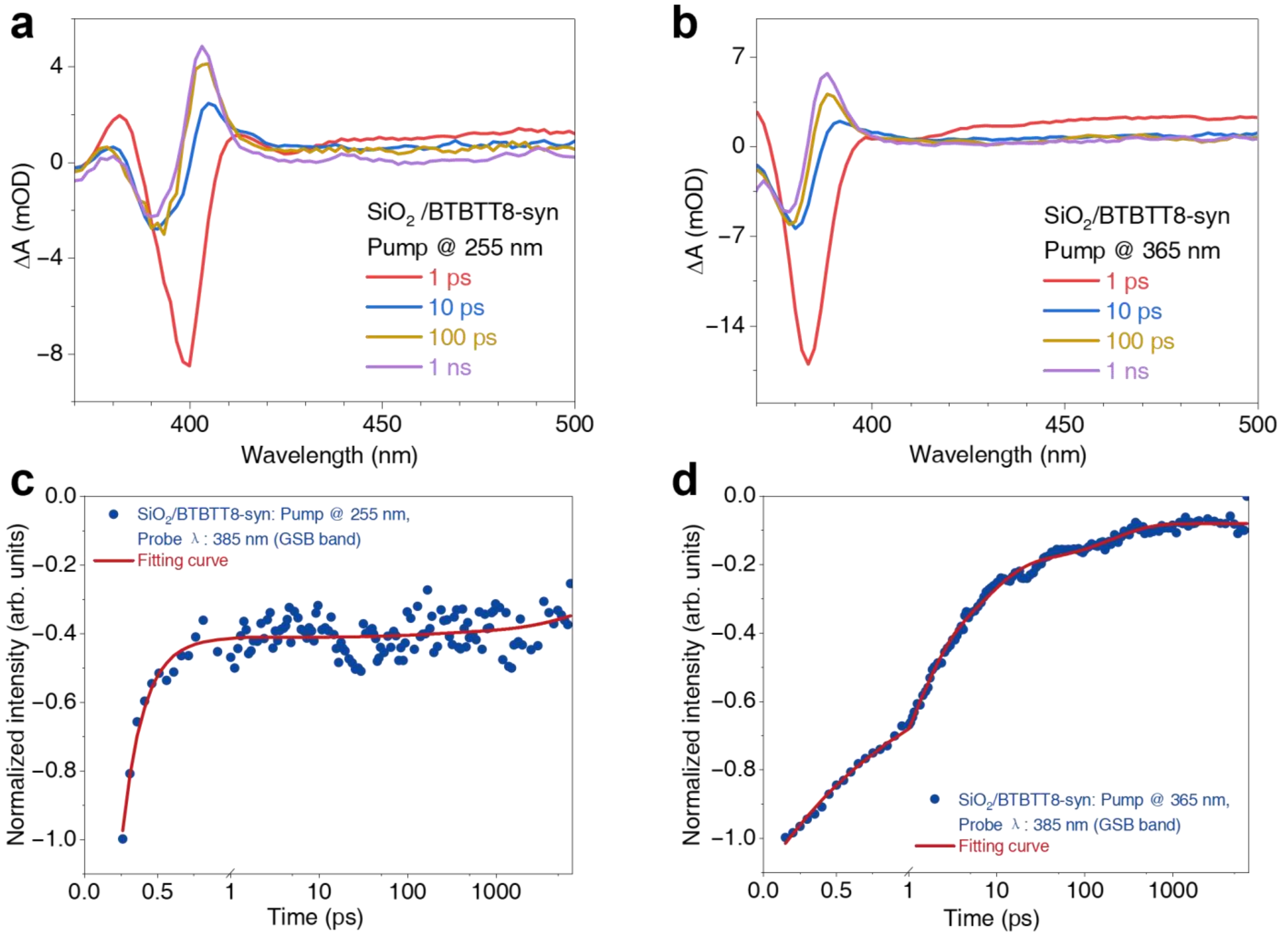

Supplementary Fig. 13. Femtosecond Transient Absorption Spectroscopy (fs-TAS) measurement. fs-TAS spectra at **a**, Pump @ 255nm and **b**, Pump @ 365nm. Normalized GSB curves extracted with a 385 nm probe, with panel **c**, excited at 255 nm and panel **d**, excited at 365 nm. Source data are provided as a Source Data file.

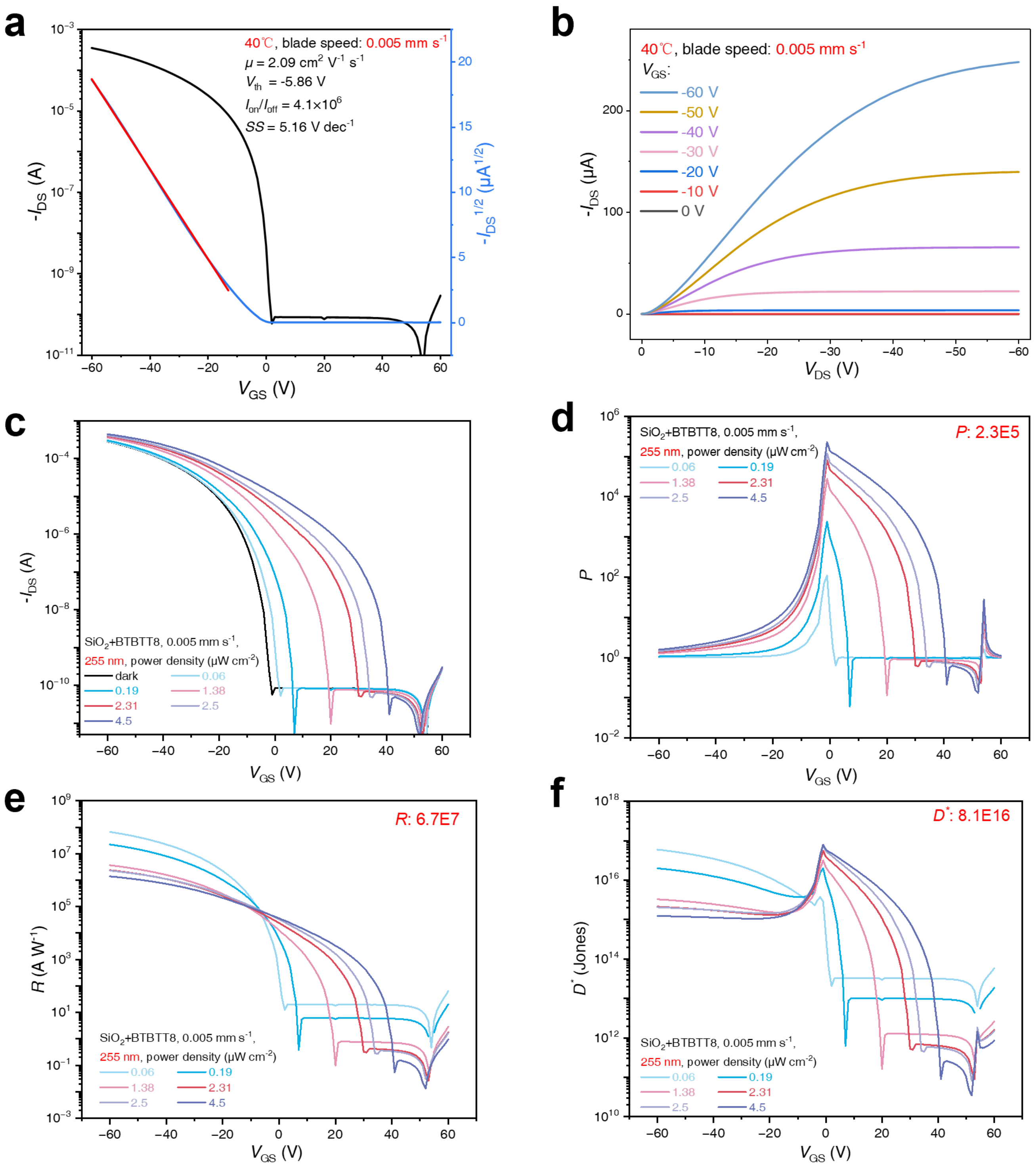

Supplementary Fig. 14. Stability test of the device after one year of storage. a, The transfer curve and **b,** output curve of the device. **c,** Light response curves and **d,** P, **e,** R and **f,** D^* curves of the device. Source data are provided as a Source Data file.

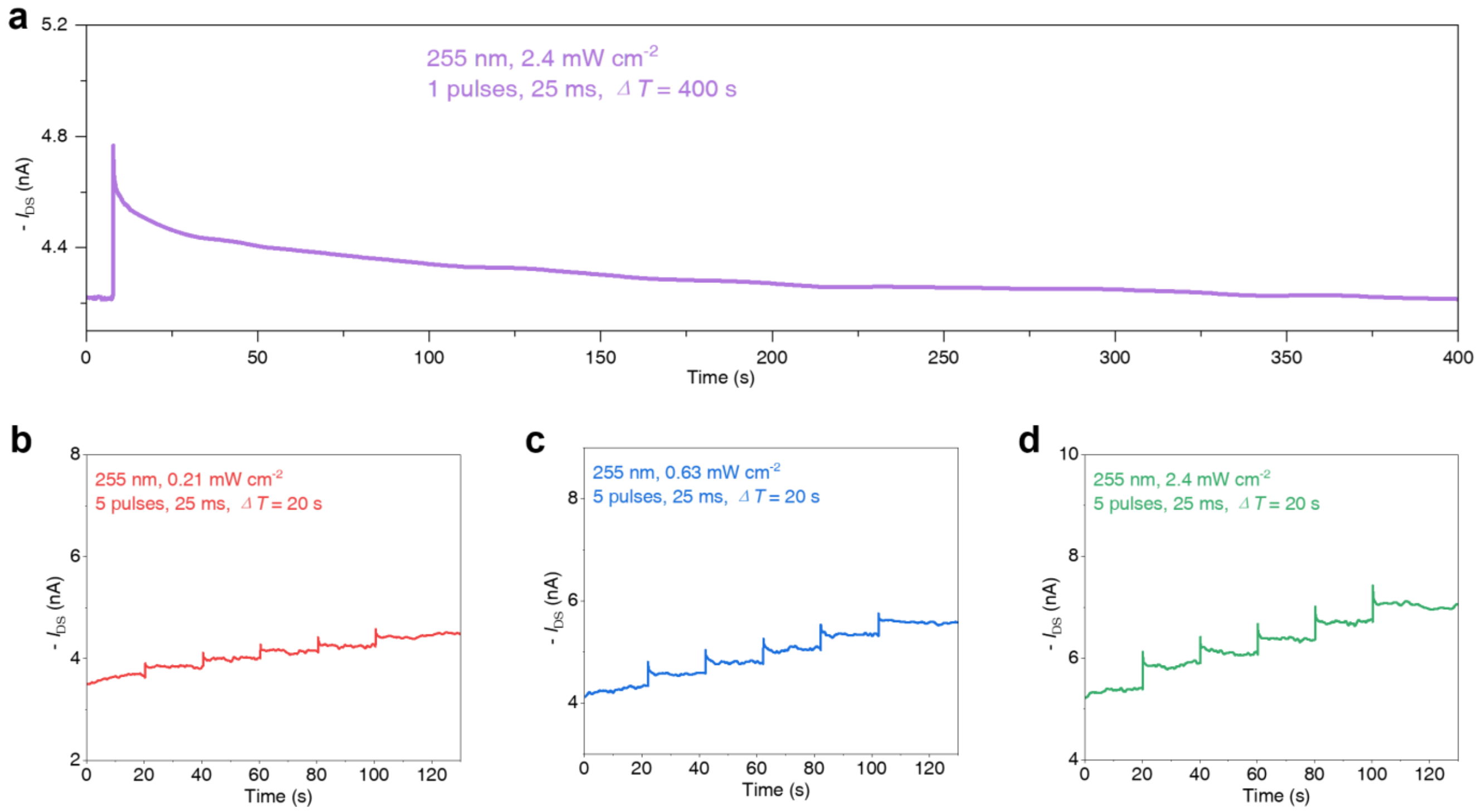

Supplementary Fig. 15. Recovery time and five periodic pulse tests for rapid response under different light intensities. **a**, Single-pulse recovery measurement (2.4 mW cm^{-2}, 25 ms pulse). **b-d**, Five-cycle periodic pulse tests at varied light intensities: **b**, 0.21 mW cm^{-2}; **c**, 0.63 mW cm^{-2}; **d**, 2.4 mW cm^{-2}. All pulses are 25 ms in width with 20 s interval. Source data are provided as a Source Data file.

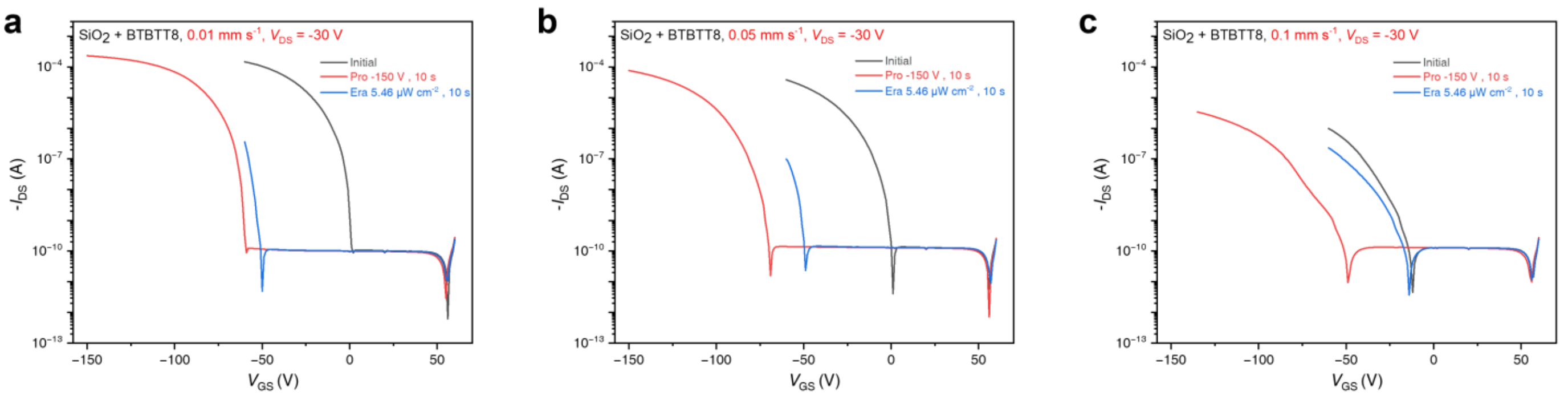

Supplementary Fig. 16. Electrical programming/optical erasure behavior of BTBTT8-syn-based devices. The blade-coating speeds of the device's semiconductor layer are as follows: **a,** 0.01 mm s^{-1}, **b,** 0.05 mm s^{-1} and **c,** 0.1 mm s^{-1}. Source data are provided as a Source Data file.

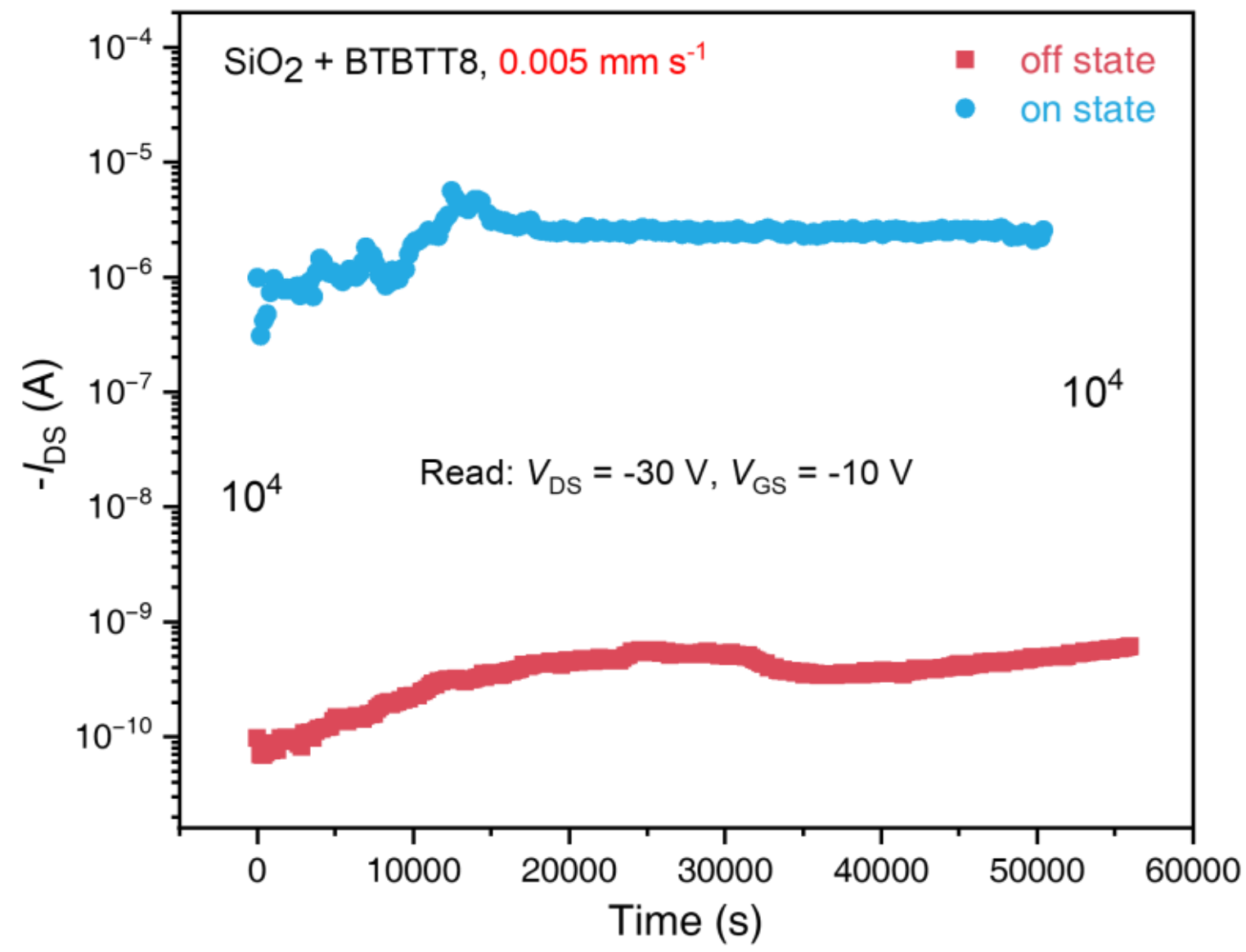

Supplementary Fig. 17. Retention time of over 50,000 seconds for electrical programming/optical erasure. Source data are provided as a Source Data file.

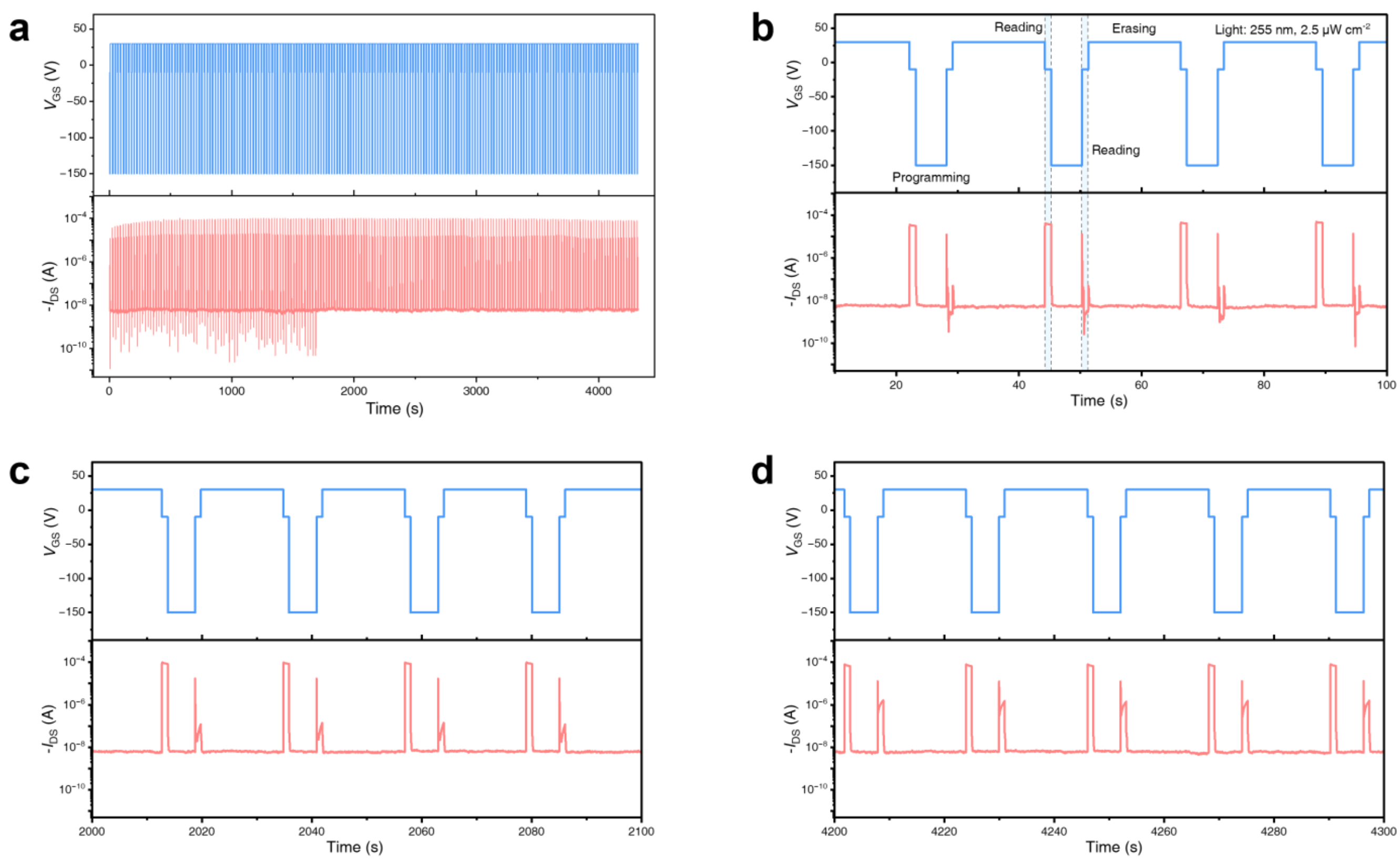

Supplementary Fig. 18. Over 4000 s of Program-Read-Erase-Read cycling of the device. Programming: V_{DS} = 0 V, V_{GS} = -150 V, 5 s. Reading: V_{DS} = -30 V, V_{GS} = -10 V, 1 s. Erasing: V_{DS} = 0 V, V_{GS} = 30 V, 15 s; light: 255 nm, 2.5 μW cm^{-2}, 10 s. **a**, Complete curve of Program-Read-Erase-Read cycle exceeding 4000 s. **b-d**, Magnified views of selected time windows from the full cycling curve: **b**, 10-100 s; **c**, 2000-2100 s; **d**, 4200-4300 s. Source data are provided as a Source Data file.

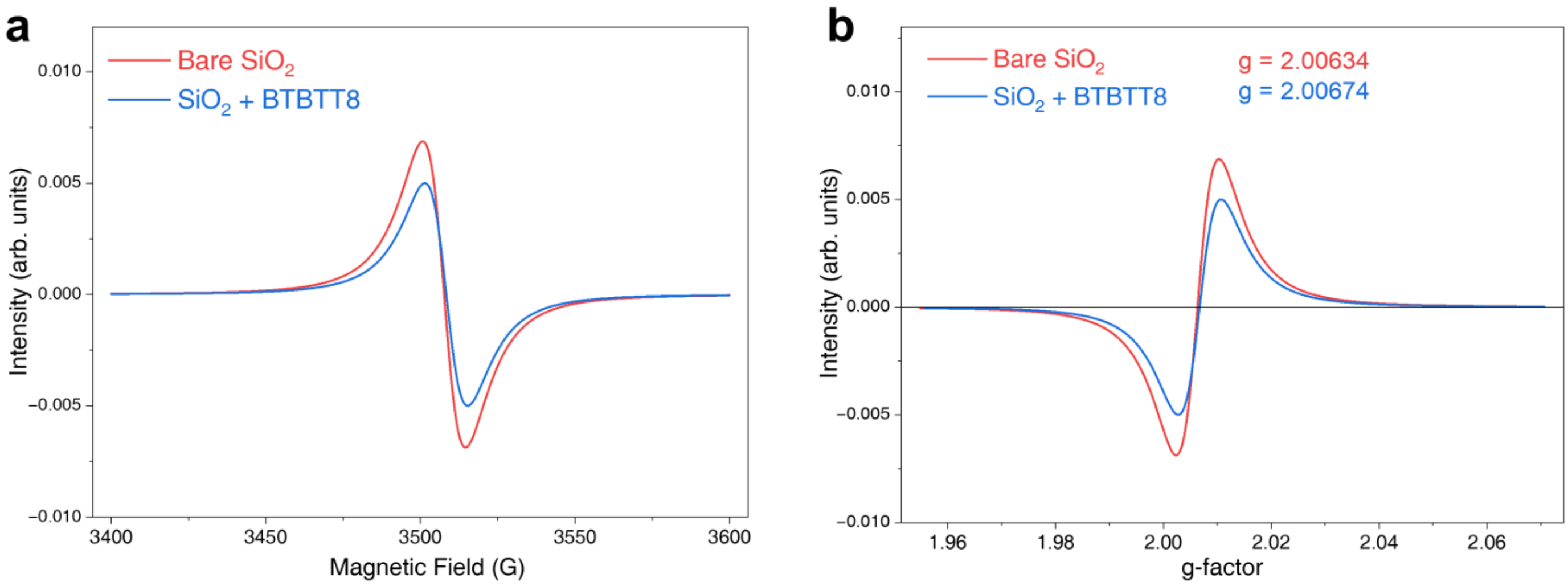

Supplementary Fig. 19. Electron paramagnetic resonance (EPR) oxygen vacancy testing and g-value at different interfaces. Source data are provided as a Source Data file.

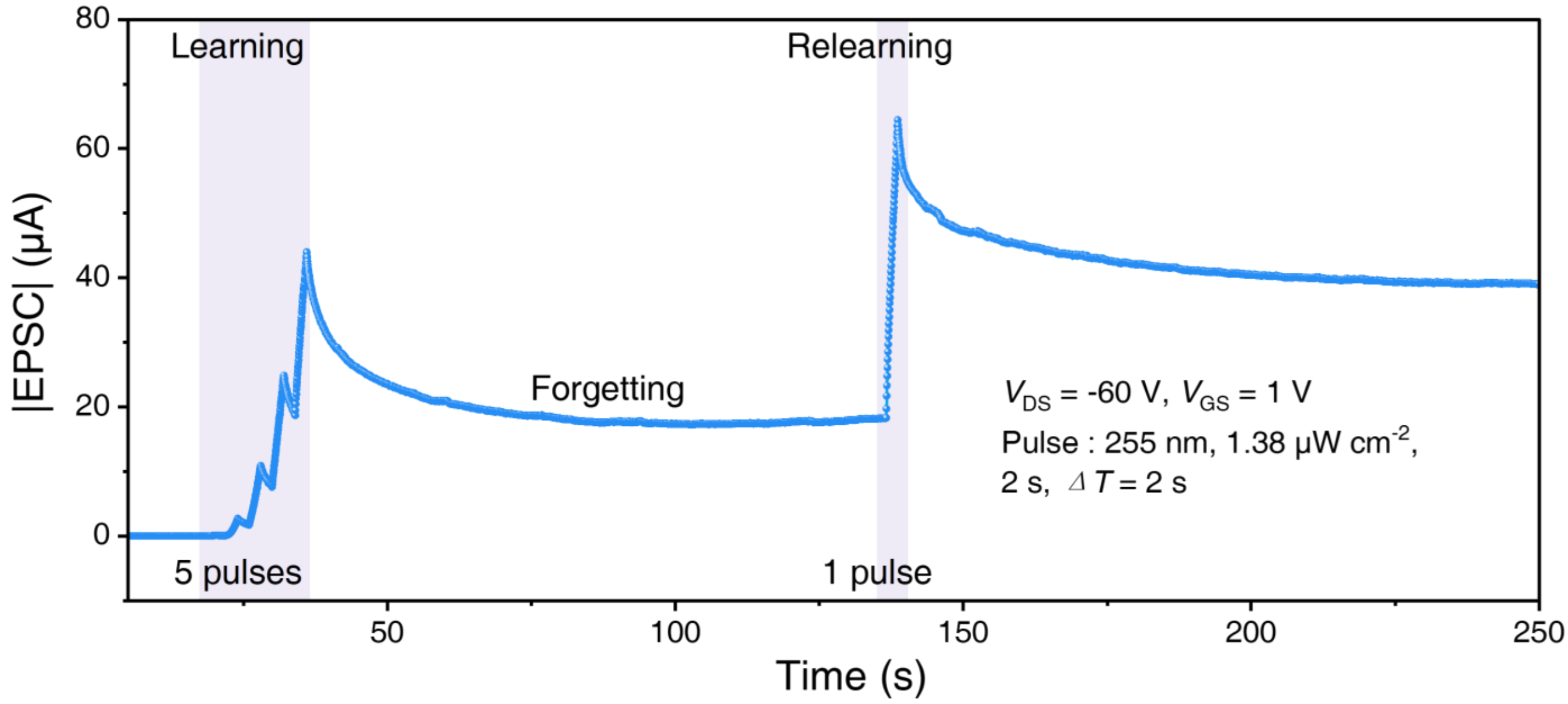

Supplementary Fig. 20. Long-term memory of learning-forgetting-relearning in the device. Source data are provided as a Source Data file.

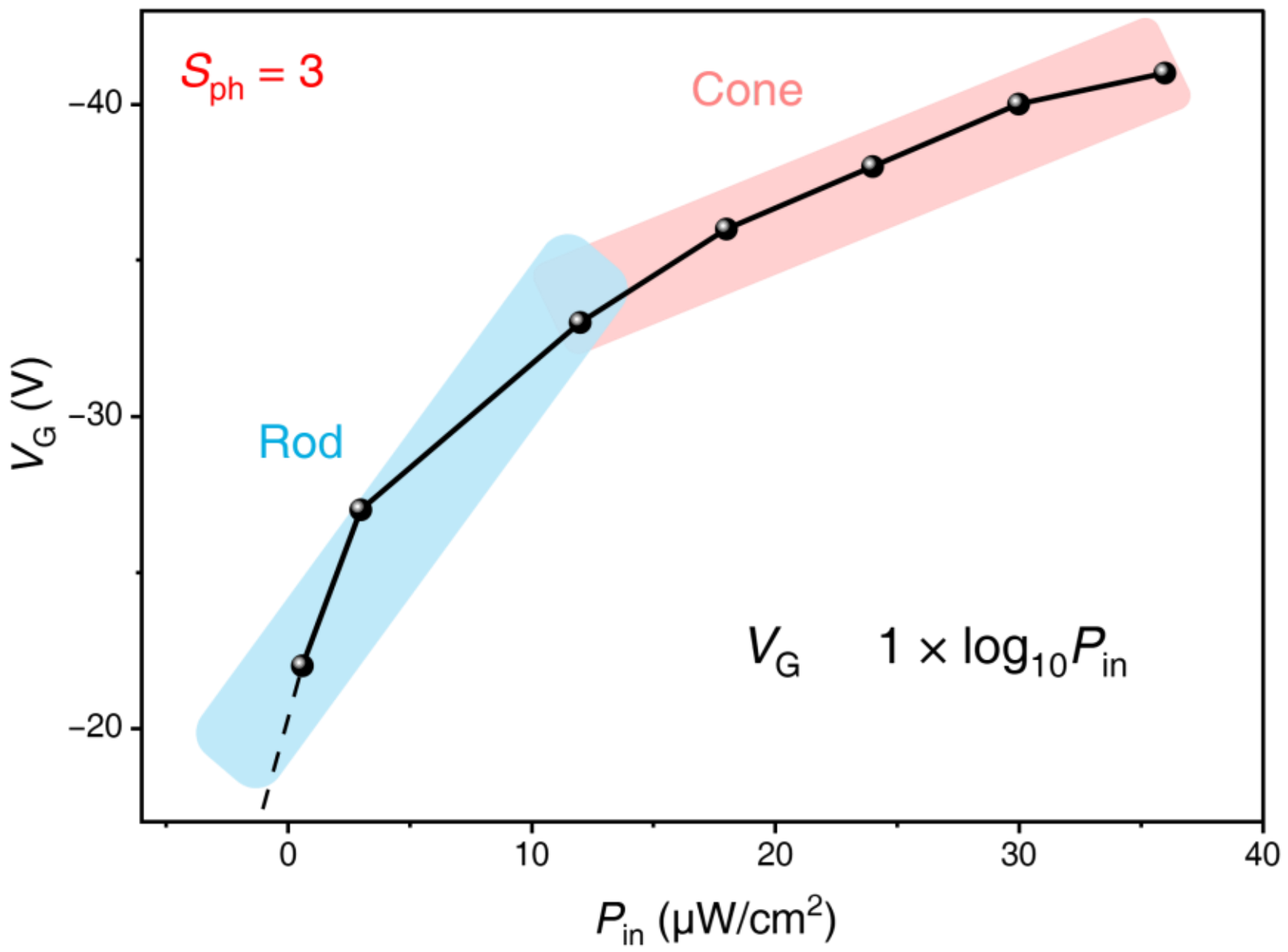

Supplementary Fig. 21. Relationship between V_{GS} and P_{in} at $S_{ph} = 3$ extracted from figure 3a. Source data are provided as a Source Data file.

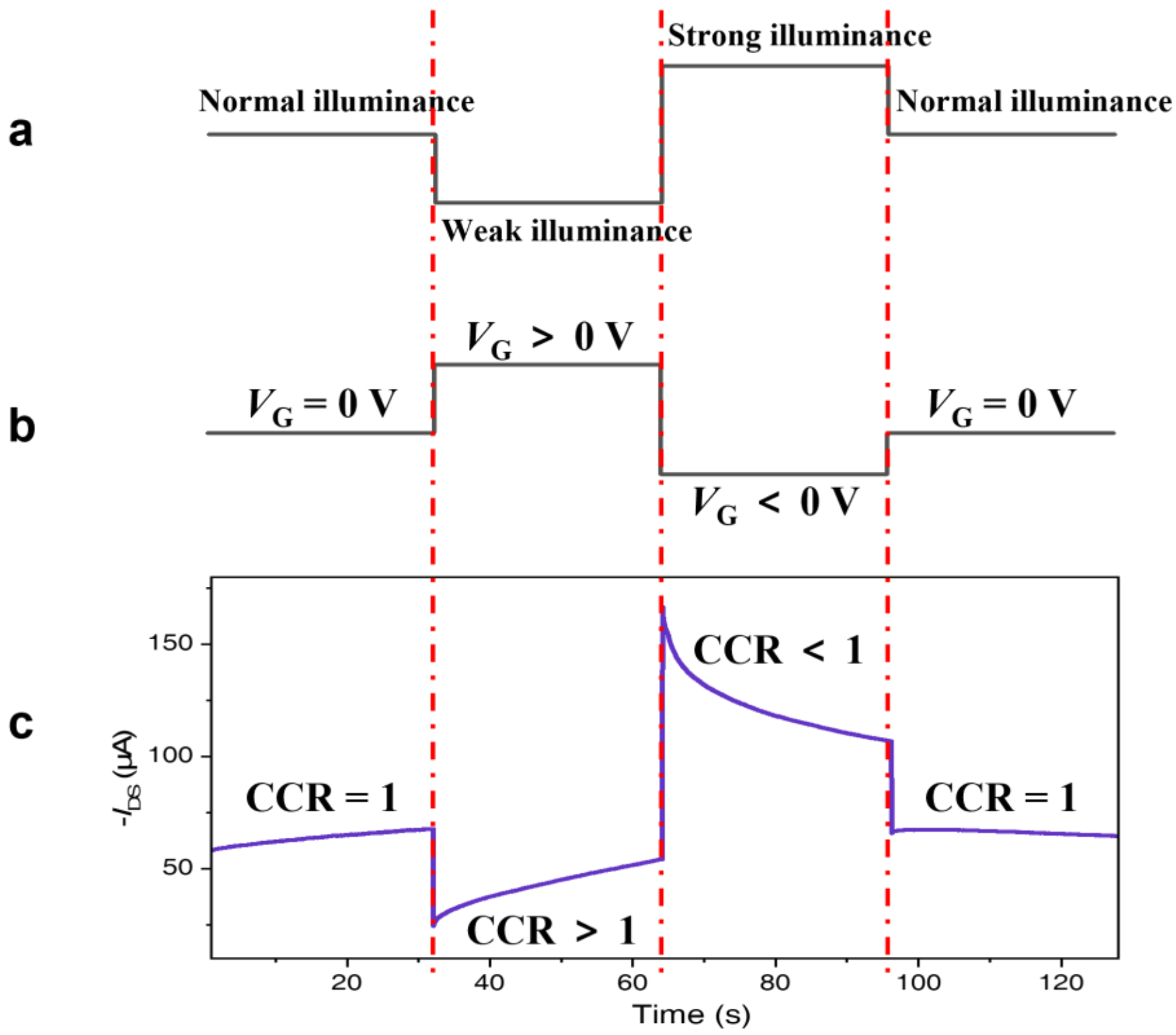

Supplementary Fig. 22. The schematic of measuring the bioinspired vision sensors for visual adaptation process according to the illuminance condition. a, The background illuminance condition. **b,** The applied V_{GS} to vision sensor array. **c,** Time-dependent current (I_{DS}) of the device over time and its current change ratio (CCR). Source data are provided as a Source Data file.

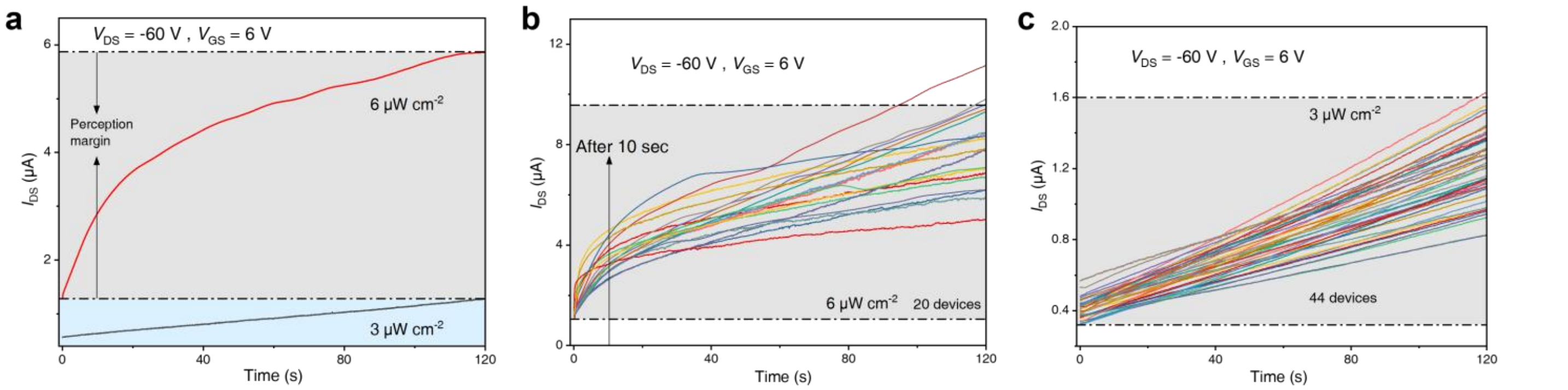

Supplementary Fig. 23. Scotopic adaptation of the phototransistor array. a, The time-dependent characteristics of phototransistor under P_{in} of 6 μW cm^{-2} and 3 μW cm^{-2} at V_{GS} = + 6 V. **b,** I_{DS} in 20 devices (bright pixels) under P_{in} of 6 μW cm^{-2} over time at V_{GS} = + 6 V. **c,** I_{DS} in 44 devices (dark pixels) under P_{in} of 3 μW cm^{-2} over time at V_{GS} = + 6 V. Source data are provided as a Source Data file.

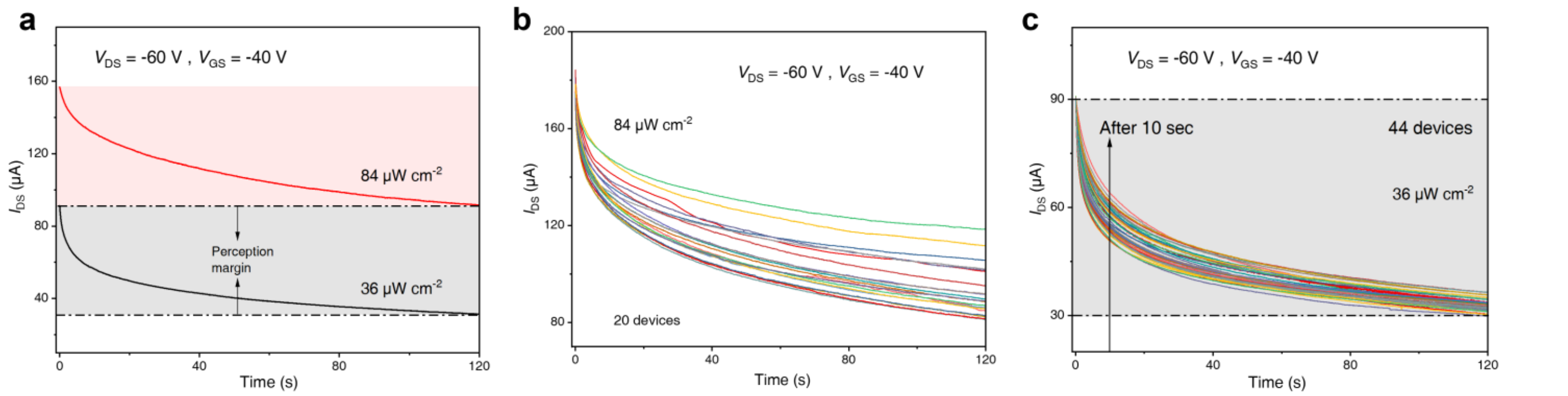

Supplementary Fig. 24. Photopic adaptation of the phototransistor array. a, The time-dependent characteristics of phototransistor under P_{in} of 84 μW cm^{-2} and 36 μW cm^{-2} at V_{GS} = - 40 V. **b,** I_{DS} in 20 devices (bright pixels) under P_{in} of 84 μW cm^{-2} over time at V_{GS} = - 40 V. **c,** I_{DS} in 44 devices (dark pixels) under P_{in} of 36 μW cm^{-2} over time at V_{GS} = - 40 V. Source data are provided as a Source Data file.

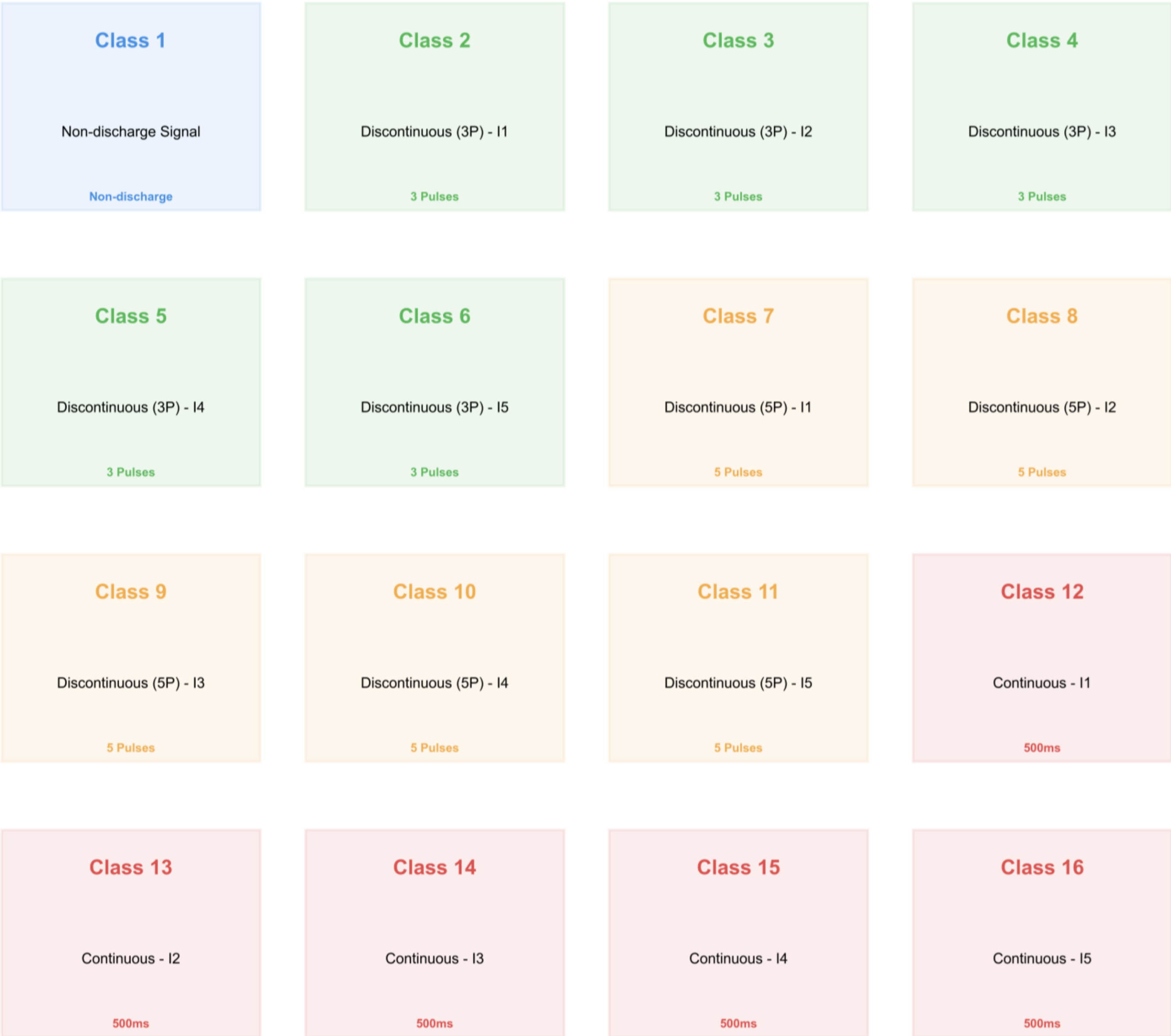

Supplementary Fig. 25. Mapping between classification labels and discharge signal types. I1: 0.06 µW cm^{-2}; I2: 0.19 µW cm^{-2}; I3: 1.4 µW cm^{-2}; I4: 2.3 µW cm^{-2}; I5: 4.5 µW cm^{-2}.

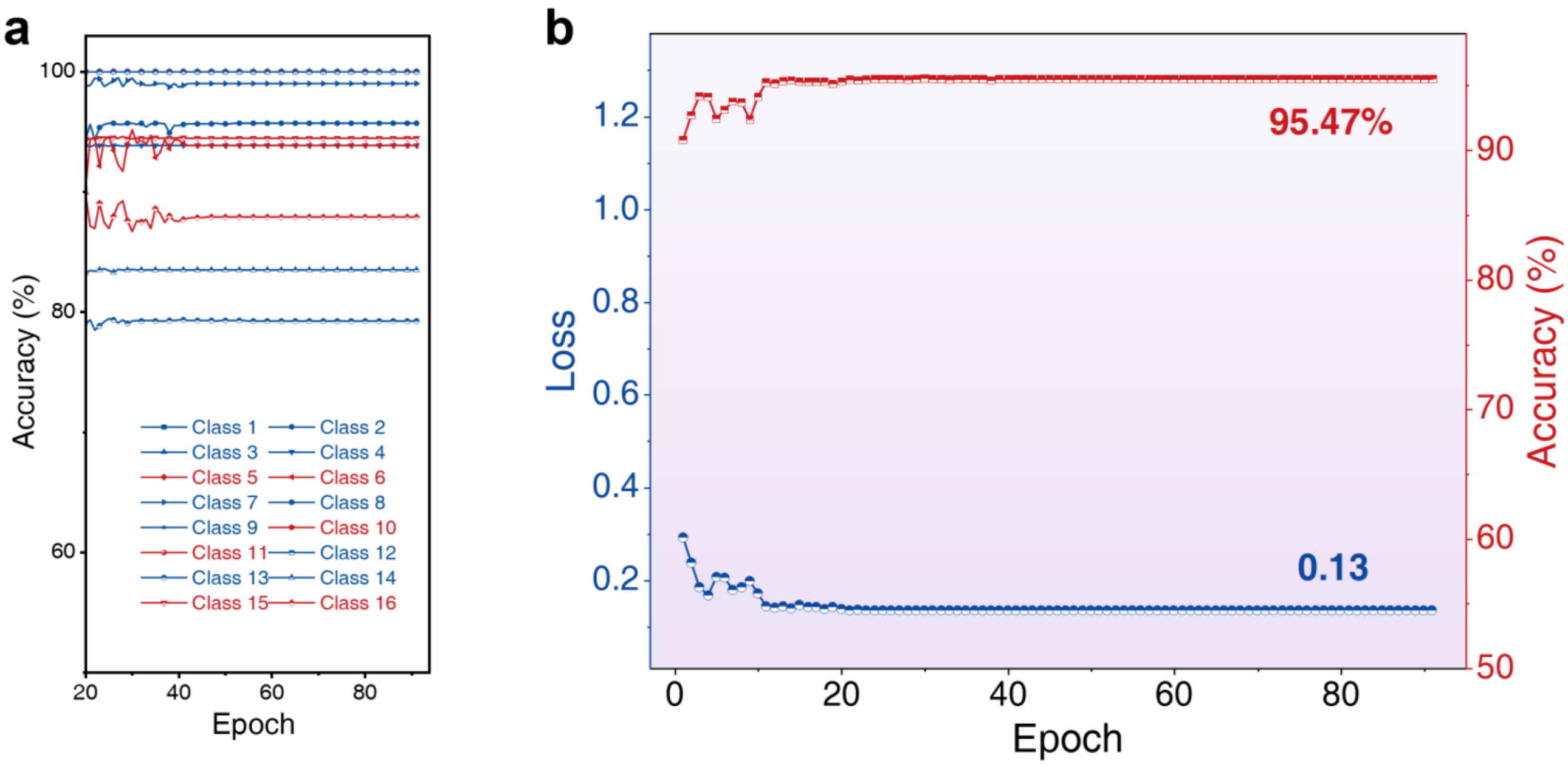

Supplementary Fig. 26. 16-Class classification accuracy and average classification accuracy of the simplified model. a, Classification accuracy of 16 classes after 91 epochs. **b,** Average classification accuracy and loss of the 16 classes after 91 epochs. Source data are provided as a Source Data file.

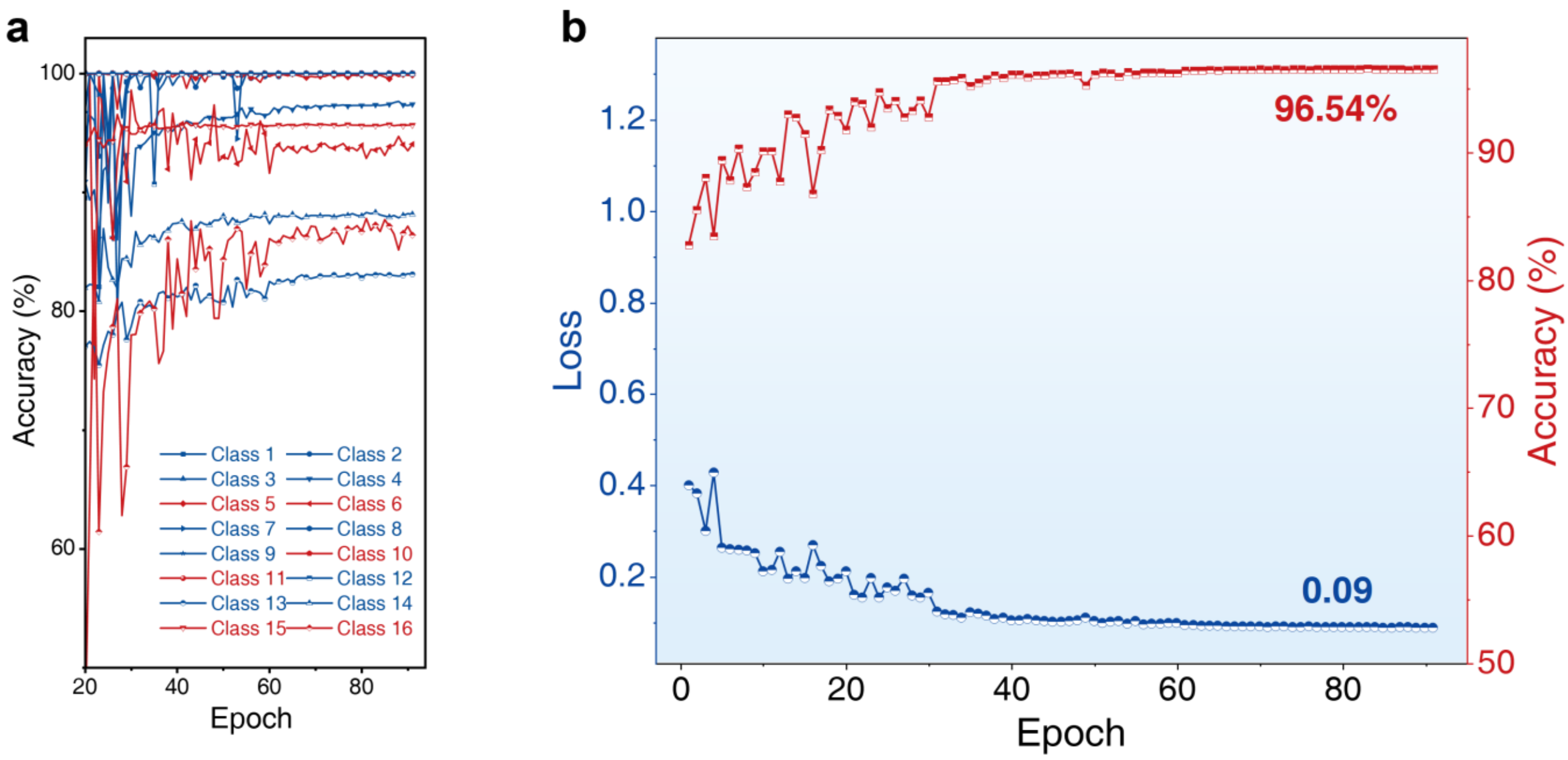

Supplementary Fig. 27. 16-Class classification accuracy and average classification accuracy of the Attention-Augmented MLP model. a, Classification accuracy of 16 classes after 91 epochs. **b,** Average classification accuracy and loss of the 16 classes after 91 epochs. Source data are provided as a Source Data file.

x	y	Signal Type (Status)
2.170e-09	2.675e-07	Discontinuous Discharge (3 Pulses) - I4 (Alert)
2.235e-09	2.618e-07	Discontinuous Discharge (3 Pulses) - I4 (Alert)
1.907e-09	2.658e-07	Discontinuous Discharge (3 Pulses) - I4 (Alert)
2.310e-09	2.653e-07	Discontinuous Discharge (3 Pulses) - I4 (Alert)
1.907e-09	2.623e-07	Discontinuous Discharge (3 Pulses) - I4 (Alert)
1.907e-09	2.673e-07	Discontinuous Discharge (3 Pulses) - I4 (Alert)
1.907e-09	2.677e-07	Discontinuous Discharge (3 Pulses) - I4 (Alert)
1.907e-09	2.701e-07	Discontinuous Discharge (3 Pulses) - I4 (Alert)
2.055e-09	2.618e-07	Discontinuous Discharge (3 Pulses) - I4 (Alert)
2.384e-09	2.700e-07	Discontinuous Discharge (3 Pulses) - I4 (Alert)
2.288e-09	1.387e-06	Discontinuous Discharge (3 Pulses) - I5 (Alert)
2.503e-09	1.391e-06	Discontinuous Discharge (3 Pulses) - I5 (Alert)
1.907e-09	1.391e-06	Discontinuous Discharge (3 Pulses) - I5 (Alert)
2.175e-09	1.403e-06	Discontinuous Discharge (3 Pulses) - I5 (Alert)
2.306e-09	1.392e-06	Discontinuous Discharge (3 Pulses) - I5 (Alert)
2.503e-09	1.390e-06	Discontinuous Discharge (3 Pulses) - I5 (Alert)
2.182e-09	1.396e-06	Discontinuous Discharge (3 Pulses) - I5 (Alert)
2.118e-09	1.390e-06	Discontinuous Discharge (3 Pulses) - I5 (Alert)
1.907e-09	1.391e-06	Discontinuous Discharge (3 Pulses) - I5 (Alert)
1.907e-09	1.399e-06	Discontinuous Discharge (3 Pulses) - I5 (Alert)
1.947e-09	7.684e-07	Discontinuous Discharge (5 Pulses) - I4 (Alert)
2.742e-09	7.992e-07	Discontinuous Discharge (5 Pulses) - I4 (Alert)
2.742e-09	7.709e-07	Discontinuous Discharge (5 Pulses) - I4 (Alert)
1.550e-09	7.992e-07	Discontinuous Discharge (5 Pulses) - I4 (Alert)
1.921e-09	7.688e-07	Discontinuous Discharge (5 Pulses) - I4 (Alert)
1.550e-09	7.992e-07	Discontinuous Discharge (5 Pulses) - I4 (Alert)
1.550e-09	7.992e-07	Discontinuous Discharge (5 Pulses) - I4 (Alert)
1.550e-09	7.980e-07	Discontinuous Discharge (5 Pulses) - I4 (Alert)
1.756e-09	7.992e-07	Discontinuous Discharge (5 Pulses) - I4 (Alert)
1.564e-09	7.753e-07	Discontinuous Discharge (5 Pulses) - I4 (Alert)
1.431e-09	3.725e-06	Discontinuous Discharge (5 Pulses) - I5 (Alert)
1.682e-09	3.684e-06	Discontinuous Discharge (5 Pulses) - I5 (Alert)
1.431e-09	3.856e-06	Discontinuous Discharge (5 Pulses) - I5 (Alert)
1.431e-09	3.698e-06	Discontinuous Discharge (5 Pulses) - I5 (Alert)
1.431e-09	3.865e-06	Discontinuous Discharge (5 Pulses) - I5 (Alert)
1.431e-09	3.870e-06	Discontinuous Discharge (5 Pulses) - I5 (Alert)
1.487e-09	3.874e-06	Discontinuous Discharge (5 Pulses) - I5 (Alert)
1.431e-09	3.872e-06	Discontinuous Discharge (5 Pulses) - I5 (Alert)
1.431e-09	3.673e-06	Discontinuous Discharge (5 Pulses) - I5 (Alert)
1.931e-09	3.881e-06	Discontinuous Discharge (5 Pulses) - I5 (Alert)
1.192e-09	2.726e-07	Continuous Discharge - I4 (Alert)
1.192e-09	2.807e-07	Continuous Discharge - I4 (Alert)
1.192e-09	2.676e-07	Continuous Discharge - I4 (Alert)
1.810e-09	2.682e-07	Continuous Discharge - I4 (Alert)
1.192e-09	2.676e-07	Continuous Discharge - I4 (Alert)
1.192e-09	2.676e-07	Continuous Discharge - I4 (Alert)
1.192e-09	2.676e-07	Continuous Discharge - I4 (Alert)
1.192e-09	2.676e-07	Continuous Discharge - I4 (Alert)
2.482e-09	2.820e-07	Continuous Discharge - I4 (Alert)
1.192e-09	2.676e-07	Continuous Discharge - I4 (Alert)
1.914e-09	1.408e-06	Discontinuous Discharge (3 Pulses) - I5 (Alert)
1.788e-09	1.565e-06	Continuous Discharge - I5 (Alert)
2.384e-09	1.415e-06	Continuous Discharge - I5 (Alert)
2.384e-09	1.532e-06	Continuous Discharge - I5 (Alert)
2.033e-09	1.556e-06	Continuous Discharge - I5 (Alert)
2.384e-09	1.517e-06	Continuous Discharge - I5 (Alert)
2.384e-09	1.400e-06	Continuous Discharge - I5 (Alert)
1.955e-09	1.474e-06	Continuous Discharge - I5 (Alert)
1.913e-09	1.572e-06	Continuous Discharge - I5 (Alert)
1.788e-09	1.394e-06	Continuous Discharge - I5 (Alert)

Supplementary Fig. 28. Alarm information automatically output by the system when classified into categories 5, 6, 10, 11, 15, and 16.

Supplementary Table 1. Solar-blind ultraviolet photodetectors based on organic materials.

Materials	Wavelength (nm)	P_{in} (μW cm^{-2})	*P*	*R* (A W^{-1})	D^* (Jones)	Reference
Cn-BTBTN	266	70	1.60×10^{7}	8.4	7.70×10^{14}	1
P3HT-PHA	254	5	-	120	4.20×10^{14}	2
C8-BTBT	280	1000	8200	44	-	3
P-MSB	254	0.3	-	27400	7.80×10^{13}	4
diF-TEG ADT	254	40	5.5	1.39×10^{-4}	-	5
Ph I: PMMA	254	500	18000	0.94	-	6
PP@GQDs/$Lu_{0.39}In_{0.61}$	256	8.6	-	0.0751	8.80×10^{11}	7
DPP-DTT	254	1250	-	-	-	8
PyDIs/BHJ	260	50	-	227	2.70×10^{11}	9
BOPNA/BHJ	270	50	1×10^{7}	2400	1.00×10^{13}	10
PVN/pentacene	254	59	-	-	-	11
PEDOT:PSS/$Lu_{0.27}Sn_{0.73}O$/ZnO	289	1.46	-	3.40×10^{-4}	-	12
PEDOT:PSS/Ga_2O_3	254	0.3	10000	0.374	9.20×10^{12}	13
Polyaniline/MgZnO	250	130	-	1.60×10^{-4}	1.50×10^{11}	14
BTBTT8-syn	255	0.06	1.1E7	4.6E7	2.9E18	This work

Supplementary Table 2. Solar-blind ultraviolet photodetectors based on p-n junction of inorganic materials.

Materials	Wavelength (nm)	P_{in} (μW cm^{-2})	P	R (A W^{-1})	D^* (Jones)	Reference
GaON/CuPC	254	100	1×10^{6}	7.90×10^{-4}	1.40×10^{13}	15
AlGaN/GaN	265	5	-	0.16	-	16
Ga_2O_3/NiO	254	270	122	2.50×10^{-4}	5.50×10^{9}	17
Ga_2O_3/Si	254	12	940	370	-	18
Ga_2O_3/PCDTBT	265	11	-	187	1.30×10^{16}	19
Ga_2O_3/GaSe	255	0.1	-	51.9	1.00×10^{14}	20
Ga_2O_3/MeOTAD	254	1	1.50×10^{5}	6.50×10^{-2}	3.95×10^{11}	21
$MgGa_2O_4$/Si	255	12	-	1.17×10^{-2}	7.30×10^{11}	22
AlZnN/graphene	255	23.6	-	2.08×10^{-2}	3.98×10^{12}	23
CuZnS/TiO_2	300	480	-	640	-	24
$CuNiO_2$/SiC	266	0.46	-	0.104	3.40×10^{12}	25
Ga_2O_3/WSe_2	254	13.51	8.99×10^{7}	2280	3.64×10^{17}	26
Ga_2O_3/NiO	260	4.65×10^{3}	4.63×10^{5}	0.415	2.27×10^{11}	27
Ga_2O_3/CuSCN	254	1.5	4.14×10^{4}	0.013	9.43×10^{11}	28
Ga_2O_3/AlGaN	254	2.00×10^{3}	3.00×10^{5}	0.360	2.00×10^{11}	29
$Al_{0.4}Ga_{0.6}N/Al_{0.65}Ga_{0.35}N$	275	13.3	1.00×10^{4}	4.00×10^{-2}	3.10×10^{12}	30
Ga_2O_3/$CuGaO_2$	254	1000	2.30×10^{4}	2.50×10^{-5}	9.00×10^{10}	31
Ga_2O_3/NiO	254	1000	900	0.59	4.30×10^{12}	32
Ga_2O_3/4H-SiC	254	91	1.66×10^{3}	1.04×10^{-2}	8.80×10^{9}	33
Ga_2O_3/GaN	254	150	152	2.84×10^{-2}	6.20×10^{10}	34
Ga_2O_3/$PtSe_2$	254	158	111	2.15×10^{-3}	5.00×10^{10}	35
Ga_2O_3/$CH_3NH_3PbI_3$	254	860	1.46×10^{3}	8.50×10^{-2}	1.30×10^{11}	36
Ga_2O_3/Si	254	39.2	55	1.38×10^{-3}	3.20×10^{10}	37

Supplementary Table 3. Solar-blind ultraviolet photodetectors based on n-n junction of inorganic materials.

Materials	Wavelength (nm)	P_{in} (μW cm^{-2})	P	R (A W^{-1})	D^* (Jones)	Reference
Ga_2O_3/diamond	220	1.20×10^5	2.20×10^4	1.80×10^{-3}	-	38
Ga_2O_3/ZnO	254	10	127	7.60×10^{-4}	-	39
Ga_2O_3/MoS_2	245	20.1	670	2.05×10^{-3}	1.20×10^{11}	40
Ga_2O_3/ZnO	251	1.67×10^3	-	9.70×10^{-3}	6.30×10^{12}	41
Ga_2O_3/Nb:$SrTiO_3$	265	0.1	5.90×10^5	1.00×10^5	2.33×10^{16}	42
Ga_2O_3/diamond	244	150	242	2.00×10^{-4}	6.90×10^9	43
Ga_2O_3/GaN	254	500	6.10×10^4	3.05	1.69×10^{13}	44
Ga_2O_3/AlN	245	0.2	3.50×10^5	0.73	2.90×10^{11}	45
Ga_2O_3/NSTO	254	45	20	43.31	-	46
Ga_2O_3/ZnO	260	10.61	1.00×10^3	9.55×10^{-2}	1.20×10^{12}	47
Ga_2O_3/AlN	254	300	2.10×10^4	9.50×10^{-5}	2.90×10^{10}	48
$Ga_2O_3/HfZrO_2$	240	0.19	-	5.26×10^{-2}	5.70×10^{12}	49

Supplementary Table 4. Solar-blind ultraviolet photodetectors based on Schottky junction of inorganic materials.

Materials	Wavelength (nm)	P_{in} (μW cm^{-2})	P	R (A W^{-1})	D^* (Jones)	Reference
AlGaN MSM	275	1	-	0.14	-	50
Ga_2O_3/graphene	254	57	-	39.3	5.92×10^{13}	51
PMA_2PbCl_4 MSM	265	0.0318	-	9	1.01×10^{11}	52
Ga_2O_3 MSM	255	400	-	0.74	-	53
Ga_2O_3 MSM	254	87	1.70×10^5	230	1.20×10^{15}	54
Ga_2O_3 Mxenes	254	1.5	1.60×10^4	1.22×10^{-2}	6.10×10^{12}	55
Ga_2O_x	254	860	-	1.95×10^4	-	56
Ga_2O_3 MSM	254	69.5	5.58×10^4	54.9	3.71×10^{14}	57
Ga_2O_3 MSM	254	200	1.00×10^5	150	-	58
Ga_2O_3/Zn	254	328.8	3.31×10^5	1.05	4.90×10^{11}	59
$Al_{0.43}Ga_{0.57}N$/Ni	254	1.78×10^4	118	1.78×10^{-2}	8.80×10^{10}	60
$Al_{0.7}Ga_{0.3}N$/graphene	250	47	-	1.13×10^{-2}	5.50×10^{11}	61
Ga_2O_3/graphene	254	1.5	100	4.85×10^{-2}	1.90×10^{10}	62
Ga_2O_3/FTO	250	166	620	9.20×10^{-3}	5.30×10^{11}	63
Ga_2O_3/In	254	200	2.50×10^6	1.55×10^{-2}	2.50×10^{11}	64

Supplementary Table 5. Solar-blind ultraviolet photodetectors based on other junction of inorganic materials.

Materials	Wavelength (nm)	P_{in} (μW cm^{-2})	*P*	*R* (A W^{-1})	D^* (Jones)	Reference
α/γ Ga_2O_3	254	100	127	2.60×10^{-4}	2.80×10^{9}	65
α/crystalline Ga_2O_3	254	1.00×10^{3}	1.00×10^{7}	0.81	5.67×10^{14}	66
LSMO/$BaTiO_3$/ Ga_2O_3	254	0.1	-	1.1	1.20×10^{12}	67
Diamond/graphene	218	150	-	21.8	1.39×10^{12}	68
MgO/MgZnO	262	1.11×10^{3}	1.00×10^{3}	48	-	69
$MoS_2/SnSe_2$	254	3.00×10^{4}	-	0.5	3.30×10^{9}	70
$Ti_3C_2T_x$/GaN	250	57	1.00×10^{5}	0.1	1.50×10^{13}	71

Supplementary Table 6. Solar-blind ultraviolet photodetectors based on single materials.

Materials	Wavelength (nm)	P_{in} (μW cm^{-2})	*P*	*R* (A W^{-1})	D^* (Jones)	Reference
GaSe	265	25	-	663	6.01×10^{11}	72
Ga_2O_3	250	1.90×10^{3}	-	8.9	3.30×10^{13}	73
Ga_2O_3	254	395	-	312.6	1.70×10^{14}	74
AlGaN	275	2.00×10^{4}	-	186	2.54×10^{14}	75
Ga_2O_3	256	1.92×10^{5}	1.00×10^{8}	300	-	76
Ga_2O_3	266	20	1.00×10^{7}	2409	-	77
Ga_2O_3	265	0.68	1.00×10^{3}	233	8.16×10^{12}	78
Ga_2O_3	256	1.31×10^{4}	1.00×10^{5}	12.4	1.90×10^{12}	79
Ga_2O_3	255	0.12	1.00×10^{3}	17	7.00×10^{12}	80
Ga_2O_3	250	98	1.00×10^{5}	852	-	81
Ga_2Ox	254	1.00×10^{3}	1.00×10^{8}	66.7	8.00×10^{15}	82
Ga_2O_3	254	1.30×10^{3}	1.00×10^{3}	2.6	1.60×10^{12}	83
Ga_2O_3	265	4.74	1.00×10^{4}	0.72	4.18×10^{11}	84
AlGaN	271	1.00×10^{4}	-	0.15	-	85
Ga_2O_3	254	100	-	22000	1.10×10^{16}	86
Ga_2O_3	254	34	1.00×10^{5}	18.43	2.48×10^{14}	87
Ga_2O_3	250	22	-	1.32×10^{6}	5.68×10^{14}	88
Ga_2O_3	254	100	147.2	2.25×10^{-2}	-	89
Ga_2O_3	254	45	5.00×10^{7}	5.67×10^{3}	-	90
KNb_3O_8	254	1.20×10^{3}	-	30	5.95×10^{11}	91
$Sr_2Nb_3O_{10}$	270	200	-	1214	1.40×10^{14}	92
$NiPS_3$	254	100	-	0.126	1.22×10^{12}	93
BiOCl	250	17.8	-	35.7	2.20×10^{10}	94
h-BN	212	1.59×10^{3}	1.00×10^{3}	0.0001	2.40E+08	95
Ga_2O_3	253	700	-	45.11	-	96
Ga_2O_3	250	70.5	-	70.26	1.26×10^{14}	97
Ga_2O_3	250	4.40×10^{-2}	6.13	96.13	-	98

Supplementary Table 7. Extracted decay time constants of ground-state bleaching signal of BTBTT8-syn under different excitation wavelengths

Excitation wavelength (nm)	Lifetime
	τ_1 = 140 fs
255	τ_2 = 163 ps
	τ_3 = 9.80 ns
	τ_1 = 1.00 ps
365	τ_2 = 7.60 ps
	τ_3 = 213 ps

Supplementary References

1. Dong, Y. *et al.* Thermally stable organic field-effect transistors based on asymmetric BTBT derivatives for high performance solar-blind photodetectors. *Adv. Sci.* **9**, 2106085 (2022).
2. Qiu, L.-Z. *et al.* Ultrathin polymer nanofibrils for solar-blind deep ultraviolet light photodetectors application. *Nano Lett.* **20**, 644-651 (2019).
3. Wu, G. *et al.* Solution-grown organic single-crystal field-effect transistors with ultrahigh response to visible-blind and deep UV signals. *Adv. Electron. Mater.* **1**, 1500136 (2015).
4. Cao, M. *et al.* Enhanced photoelectrical response of thermodynamically epitaxial organic crystals at the two-dimensional limit. *Nat. Commun.* **10**, 756 (2019).
5. Guo, W. *et al.* Solution-processed low-voltage organic phototransistors based on an anthradithiophene molecular solid. *Org. Electron.* **15**, 3061-3069 (2014).
6. Kang, M. *et al.* Light-sensitive charge storage medium with spironaphthooxazine molecule-polymer blends for dual-functional organic phototransistor memory. *Org. Electron.* **78**, 105554 (2020).
7. Cai, H. *et al.* Graphene quantum dots decorated organic conductive polymer for advanced ternary amorphous solar-blind UV photovoltaic photodetectors. *Chem. Eng. J.* **506**, 159844 (2025).
8. Zhao, X., Wei, S. Y., Wang, X. H. & Qiu, L. Z. A novel multilevel nonvolatile solar-blind deep ultraviolet photoelectric memory based on an organic field effect transistor. *Adv. Opt. Mater.* **9**, 2002256 (2021).
9. Wang, J. *et al.* High performance solar-blind uv detectors based on n-type wide bandgap organic materials. *ACS Mater. Lett.* **6**, 5023-5030 (2024).
10. Wang, J. *et al.* An ultrasensitive narrowband organic phototransistor for solar-blind ultraviolet detection and imaging. *J. Mater. Chem. C* **13**, 892-901 (2025).
11. Zhang, Z. D. *et al.* Selective solar-blind UV monitoring based on organic field-effect transistor nonvolatile memories. *Adv. Electron. Mater.* **3**, 1700052 (2017).
12. Zhang, D. *et al.* High selectivity and wide range uv photodetection in PEDOT:PSS/ $Lu_{0.27}Sn_{0.73}O$/ZnO heterojunction. *IEEE Trans. Electron Devices* **71**, 2491-2496 (2024).
13. Li, S. *et al.* A self-powered solar-blind photodetector with largeVocenhancing performance based on the PEDOT:PSS/Ga_2O_3 organic–inorganic hybrid heterojunction. *J. Mater. Chem. C* **8**, 1292-1300 (2020).
14. Chen, H. *et al.* Ultrasensitive self-powered solar-blind deep-ultraviolet photodetector based on all-solid-state polyaniline/MgZnO bilayer. *Small* **12**, 5809-5816 (2016).
15. Wu, C. *et al.* Enhanced performance of gallium-based wide bandgap oxide semiconductor heterojunction photodetector for solar-blind optical communication via oxygen vacancy electrical activity modulation. *Adv. Opt. Mater.* **12**, 2302294 (2024).
16. Yu, H. *et al.* Dual-functional triangular-shape micro-size light-emitting and detecting diode for on-chip optical communication in the deep ultraviolet band. *Laser Photonics Rev.* **18**, 2300789 (2024).
17. Yu, J. *et al.* Improved photoresponse performance of self-powered β-Ga_2O_3/NiO heterojunction UV photodetector by surface plasmonic effect of Pt nanoparticles. *IEEE Trans. Electron Devices* **67**, 3199-3204 (2020).
18. Guo, X. C. *et al.* β -Ga_2O_3 / p -Si heterojunction solar-blind ultraviolet photodetector with enhanced photoelectric responsivity. *J. Alloys Compd.* **660**, 136-140 (2016).
19. Wang, Y. *et al.* Multifunctional solar-blind ultraviolet photodetectors based on p-PCDTBT/n-Ga_2O_3 heterojunction with high photoresponse. *InfoMat* **6**, e12503 (2023).
20. Wang, Y. *et al.* p-GaSe/n-Ga_2O_3 van der Waals heterostructure photodetector at solar-blind wavelengths with ultrahigh responsivity and detectivity. *ACS Photonics* **8**, 2256-2264 (2021).

21. Yan, Z. *et al.* High sensitivity and fast response self-powered solar-blind ultraviolet photodetector with a β-Ga_2O_3/spiro-MeOTAD p–n heterojunction. *J. Mater. Chem. C* **8**, 4502-4509 (2020).
22. Huang, J. *et al.* High performance self-powered solar-blind photodetector based on amorphous $MgGa_2O_4$/p-Si heterojunction. *Mater. Lett.* **359**, 135898 (2024).
23. Lin, J. *et al.* Amorphous-AlZnN/graphene heterostructure for solar-blind ultraviolet photovoltaic detectors. *Ceram. Int.* **49**, 4177-4183 (2023).
24. Xu, X. *et al.* A real-time wearable UV-radiation monitor based on a high-performance p-CuZnS/n-TiO_2 photodetector. *Adv. Mater.* **30**, 1803165 (2018).
25. Deng, W. *et al.* Ultrasensitive and ultrafast self-powered ultraviolet photodetector array for solar-blind and weak-light imaging. *Adv. Mater.* **1**, E14968 (2025).
26. Zou, Y. *et al.* Alleviating the Responsivity-Speed Dilemma of Photodetectors via Opposite Photogating Engineering with an Auxiliary Light Source beyond the Chip. *International Electron Devices Meeting (IEDM)* **1**, 19.15.11 (2022).
27. Li, K. *et al.* Deep-ultraviolet photodetection using single-crystalline β-Ga_2O_3/NiO heterojunctions. *ACS Appl. Mater. Interfaces* **11**, 35095-35104 (2019).
28. Li, S. *et al.* Ultrasensitive, superhigh signal-to-noise ratio, self-powered solar-blind photodetector based on n-Ga_2O_3/p-CuSCN core–shell microwire heterojunction. *ACS Appl. Mater. Interfaces* **11**, 35105-35114 (2019).
29. He, X. *et al.* Excellent wavelength selectivity of p-AlGaN/n-Ga_2O_3 solar-blind UVC PD. *ACS Appl. Mater. Interfaces* **16**, 64146-64155 (2024).
30. Gu, Y. *et al.* Self-powered AlGaN-based MSM solar-blind ultraviolet photodetectors with high Al-content AlxGa1−xN/AlyGa1−yN asymmetrical heterostructure. *Appl. Phys. Lett.* **123**, 232103 (2023).
31. Wu, C. *et al.* High sensitive and stable self-powered solar-blind photodetector based on solution-processed all inorganic $CuMO_2$/Ga_2O_3 pn heterojunction. *Mater. Today Phys.* **17**, 100335 (2021).
32. Woo, S. *et al.* High-performance self-powered deep ultraviolet photodetector based on NiO/β-Ga_2O_3 heterojunction with high responsivity and selectivity. *Phys. Status Solidi A.* **221**, 2400310 (2024).
33. Yu, J. *et al.* Self-powered photodetectors based on β-Ga_2O_3/4H–SiC heterojunction with ultrahigh current on/off ratio and fast response. *J. Alloys Compd.* **821**, 153532 (2020).
34. Li, P. *et al.* Construction of GaN/Ga_2O_3 p–n junction for an extremely high responsivity self-powered UV photodetector. *J. Mater. Chem. C* **5**, 10562-10570 (2017).
35. Rong, J. *et al.* Mixed-Dimensional 2D $PtSe_2$/3D a-Ga_2O_3 heterojunction for self-driven broadband photodetector with high responsivity in UV region. *Phys. Status Solidi A.* **222**, 2400006 (2024).
36. Dong, L. *et al.* Performance-enhanced solar-blind photodetector based on a $CH_3NH_3PbI_3$/β-Ga_2O_3 hybrid structure. *J. Mater. Chem. C* **7**, 14205-14211 (2019).
37. Han, Y. *et al.* A self-powered solar-blind UV-enhanced Bi_2Se_3/a-Ga_2O_3/p-Si heterojunction photodetector for full spectral photoresponse and imaging. *Nano Res.* **17**, 2960-2970 (2023).
38. Li, K. *et al.* Solar-blind position-sensitive detectors fabricated from β-Ga_2O_3/polycrystalline diamond heterojunctions. *Phys. Status Solidi RRL* **15**, 2100347 (2021).
39. Wu, Z. *et al.* A self-powered deep-ultraviolet photodetector based on an epitaxial Ga_2O_3/Ga:ZnO heterojunction. *J. Mater. Chem. C* **5**, 8688-8693 (2017).
40. Zhuo, R. *et al.* A self-powered solar-blind photodetector based on a MoS_2/β-Ga_2O_3 heterojunction. *J. Mater. Chem. C* **6**, 10982-10986 (2018).
41. Zhao, B. *et al.* An ultrahigh responsivity (9.7 mA W−1) self-powered solar-blind photodetector based on individual ZnO–Ga_2O_3 heterostructures. *Adv. Funct. Mater.* **27**, 1700264 (2017).
42. Zhang, Q. *et al.* Enhanced gain and detectivity of unipolar barrier solar blind avalanche photodetector via lattice

and band engineering. *Nat. Commun.* **14**, 418 (2023).
43. Chen, Y.-C. *et al.* Self-powered diamond/β-Ga_2O_3 photodetectors for solar-blind imaging. *J. Mater. Chem. C* **6**, 5727-5732 (2018).
44. Guo, D. *et al.* Self-powered ultraviolet photodetector with superhigh photoresponsivity (3.05 A/W) based on the GaN/Sn:Ga_2O_3 pn junction. *ACS Nano* **12**, 12827-12835 (2018).
45. Wang, H. *et al.* High-sensitivity and fast-response solar-blind photodetectors via band offset engineering for motion tracking. *Nat. Commun.* **16**, 8175 (2025).
46. Guo, D. *et al.* Zero-power-consumption solar-blind photodetector based on β-Ga_2O_3/NSTO heterojunction. *ACS Appl. Mater. Interfaces* **9**, 1619-1628 (2017).
47. Wang, H. *et al.* Ferroelectricity enhanced self-powered solar-blind UV photodetector based on Ga_2O_3/ZnO:V heterojunction. *Mater. Today Phys.* **30**, 100929 (2023).
48. Hao, Y., Wang, X. & An, Y. A Ga_2O_3/AlN heterojunction for self-powered solar-blind photodetection with high photo-to-dark current ratio and fast response speed. *Phys. Scr.* **96**, 125835 (2021).
49. Yan, S. *et al.* High-performance self-driven solar-blind ultraviolet photodetectors based on $HfZrO_2$/β-Ga_2O_3 heterojunctions. *ACS Appl. Mater. Interfaces* **15**, 22263-22273 (2023).
50. Xie, F. *et al.* Ultra-low dark current AlGaN-based solar-blind metal–semiconductor–metal photodetectors for high-temperature applications. *IEEE Sens. J.* **12**, 2086-2090 (2012).
51. Kong, W. Y. *et al.* Graphene-β-Ga_2O_3 heterojunction for highly sensitive deep UV photodetector application. *Adv. Mater.* **28**, 10725-10731 (2016).
52. Guo, L. *et al.* Ferro-pyro-phototronic effect in monocrystalline 2D ferroelectric perovskite for high-sensitive, self-powered, and stable ultraviolet photodetector. *ACS Nano* **16**, 1280-1290 (2022).
53. Tak, B. R. *et al.* High-temperature photocurrent mechanism of β-Ga_2O_3 based metal-semiconductor-metal solar-blind photodetectors. *J. Appl. Phys.* **125**, 144501 (2019).
54. Qin, Y. *et al.* Metal–semiconductor–metal ε-Ga_2O_3 solar-blind photodetectors with a record-high responsivity rejection ratio and their gain mechanism. *ACS Photonics* **7**, 812-820 (2020).
55. Chen, Y. *et al.* Solar-blind photodetectors based on MXenes–β-Ga_2O_3 Schottky junctions. *J. Phys. D: Appl. Phys.* **53**, 484001 (2020).
56. Hou, X. *et al.* Pyroelectric photoconductive diode for highly sensitive and fast DUV detection. *Adv. Mater.* **36**, 2314249 (2024).
57. Qian, L. *et al.* High-sensitivity β-Ga_2O_3 solar-blind photodetector on high-temperature pretreated c-plane sapphire substrate. *Opt. Mater. Express* **7**, 3643 (2017).
58. Xu, Y. *et al.* Solar blind deep ultraviolet β-Ga_2O_3 photodetectors grown on sapphire by the Mist-CVD method. *Opt. Mater. Express* **8**, 2941 (2018).
59. Li, Y. *et al.* Graphene interdigital electrodes for improving sensitivity in a Ga_2O_3:Zn deep-ultraviolet photoconductive detector. *ACS Appl. Mater. Interfaces* **11**, 1013-1020 (2018).
60. Lin, T. *et al.* AlGaN-based self-powered solar-blind UV photodetectors with Ni/Au electrodes. *Eur. Phys. J. Spec. Top.* **234**, 369-374 (2025).
61. Pandit, B. & Cho, J. Solar-blind ultraviolet photodetectors with thermally reduced graphene oxide formed on high-Al-content AlGaN layers. *AIP Adv.* **11**, 115322 (2021).
62. Chen, Y. *et al.* Ga_2O_3-Based solar-blind position-sensitive detector for noncontact measurement and optoelectronic demodulation. *Nano Lett.* **22**, 4888-4896 (2022).
63. Li, Y. *et al.* High-performance Ga_2O_3/FTO-based self-driven solar-blind UV photodetector with thickness-optimized graphene top electrode. *J. Mater. Res. Technol.* **22**, 2174-2185 (2023).
64. Yan, Z.-Y. *et al.* Ti_3C_2/ϵ-Ga_2O_3 schottky self-powered solar-blind photodetector with robust responsivity. *IEEE*

J. Sel. Top. Quantum Electron. **28**, 1-8 (2022).

65. Wu, C. *et al.* Vertical α/β-Ga_2O_3 phase junction nanorods array with graphene-silver nanowire hybrid conductive electrode for high-performance self-powered solar-blind photodetectors. *Mater. Today Phys.* **12**, 100193 (2020).
66. Wang, Y. *et al.* One-step growth of amorphous/crystalline Ga_2O_3 phase junctions for high-performance solar-blind photodetection. *ACS Appl. Mater. Interfaces* **11**, 45922-45929 (2019).
67. Wu, H. *et al.* Ultrasensitive deep-ultraviolet photodetectors based on band engineering and ferroelectric modulation. *Adv. Mater.* **37**, 2412717 (2025).
68. Lin, C. N. *et al.* Diamond-based all-carbon photodetectors for solar-blind imaging. *Adv. Opt. Mater.* **6**, 1800068 (2018).
69. Chen, H. *et al.* Avalanche solar blind photodetectors with high responsivity based on MgO/MgZnO heterostructures. *Opt. Mater. Express* **8**, 785 (2018).
70. Yu, Y. *et al.* Doping engineering in the MoS_2/$SnSe_2$ heterostructure toward high-rejection-ratio solar-blind UV photodetection. *Adv. Mater.* **34**, 2206486 (2022).
71. Song, W., Chen, J., Li, Z. & Fang, X. Self-powered MXene/GaN van der Waals heterojunction ultraviolet photodiodes with superhigh efficiency and stable current outputs. *Adv. Mater.* **33**, 2101059 (2021).
72. Wu, C. Y. *et al.* Non-ultrawide bandgap semiconductor GaSe nanobelts for sensitive deep ultraviolet light photodetector application. *Small* **18**, 2200594 (2022).
73. Chen, Y. *et al.* 3D Solar-blind Ga_2O_3 photodetector array realized via origami method. *Adv. Funct. Mater.* **29**, 1906040 (2019).
74. Luo, L. *et al.* Ultra-fast gallium oxide solar-blind photodetector with novel thermal pulse treatment. *Adv. Mater.* **37**, 2414130 (2025).
75. He, R. *et al.* Monolithically integrated UVC AlGaN-based multiple quantum wells structure and photonic chips for solar-blind communications. *Nano Energ.* **104**, 107928 (2022).
76. Zhou, Y. *et al.* Solar-blind photodetector arrays fabricated by weaving strategy. *ACS Nano* **18**, 7610-7617 (2024).
77. Lu, Y.-C. *et al.* High-performance solar-blind photodetector arrays constructed from Sn-doped Ga_2O_3 microwires via patterned electrodes. *Nano Res.* **15**, 7631-7638 (2022).
78. Xie, C. *et al.* Catalyst-free vapor–solid deposition growth of β-Ga_2O_3 nanowires for DUV photodetector and image sensor application. *Adv. Opt. Mater.* **7**, 1901257 (2019).
79. Chen, Y.-C. *et al.* Ga_2O_3 photodetector arrays for solar-blind imaging. *J. Mater. Chem. C* **7**, 2557-2562 (2019).
80. Hu, G. C. *et al.* High gain Ga_2O_3 solar-blind photodetectors realized via a carrier multiplication process. *Opt. Express* **23**, 13554 (2015).
81. Zou, R. *et al.* High detectivity solar-blind high-temperature deep-ultraviolet photodetector based on multi-layered (l00) facet-oriented β-Ga_2O_3 nanobelts. *Small* **10**, 1848-1856 (2014).
82. Hou, X. *et al.* High-performance harsh-environment-resistant GaOX solar-blind photodetectors via defect and doping engineering. *Adv. Mater.* **34**, 2106923 (2021).
83. Li, M.-Q. *et al.* Highly preferred orientation of Ga_2O_3 films sputtered on SiC substrates for deep UV photodetector application. *Appl. Surf. Sci.* **471**, 694-702 (2019).
84. Xie, C. *et al.* Patterned growth of β-Ga_2O_3 thin films for solar-blind deep-ultraviolet photodetectors array and optical imaging application. *J. Mater. Sci. Technol.* **72**, 189-196 (2021).
85. Chen, Y. *et al.* The optimized growth of AlN templates for back-illuminated AlGaN-based solar-blind ultraviolet photodetectors by MOCVD. *J. Mater. Chem. C* **6**, 4936-4942 (2018).
86. Xu, Y. *et al.* Ultrahigh-performance solar-blind photodetectors based on high quality heteroepitaxial single crystalline β-Ga_2O_3 film grown by vacuumfree, low-cost mist chemical vapor deposition. *Adv. Mater. Technol.* **6**, 2001296 (2021).

87. Qian, L. *et al.* Comprehensively improved performance of β-Ga_2O_3 solar-blind photodetector enabled by a homojunction with unique passivation mechanisms. *ACS Appl. Mater. Interfaces* **13**, 40837-40846 (2021).
88. Li, X.-X. *et al.* High responsivity and flexible deep-UV phototransistor based on Ta-doped β-Ga_2O_3. *npj Flexible Electron.* **6**, 47 (2022).
89. Zhang, W. *et al.* Self-supported β-Ga_2O_3 nanowires and for stretchable solar-blind UV photodetectors. *Sci. Rep.* **15**, 17416 (2025).
90. Han, Z. *et al.* Boosted UV photodetection performance in chemically etched amorphous Ga_2O_3 thin-film transistors. *Adv. Opt. Mater.* **8**, 1901833 (2020).
91. Ping, Y. *et al.* Polarization sensitive solar-blind ultraviolet photodetectors based on ultrawide bandgap KNb_3O_8 nanobelt with fringe-like atomic lattice. *Adv. Funct. Mater.* **32**, 2111673 (2022).
92. Li, S. *et al.* 2D Perovskite $Sr_2Nb_3O_{10}$ for high-performance UV photodetectors. *Adv. Mater.* **32**, 1905443 (2019).
93. Chu, J. *et al.* High-Performance ultraviolet photodetector based on a few-layered 2D NiPS3 nanosheet. *Adv. Funct. Mater.* **27**, 1701342 (2017).
94. Han, W. *et al.* Atomically thin oxyhalide solar-blind photodetectors. *Small* **16**, 2000228 (2020).
95. Liu, H. *et al.* High-performance deep ultraviolet photodetectors based on few-layer hexagonal boron nitride. *Nanoscale* **10**, 5559-5565 (2018).
96. Lee, S. H. *et al.* High-responsivity deep-ultraviolet-selective photodetectors using ultrathin gallium oxide films. *ACS Photonics* **4**, 2937-2943 (2017).
97. Qian, L. *et al.* Ultrahigh-responsivity, rapid-recovery, solar-blind photodetector based on highly nonstoichiometric amorphous gallium oxide. *ACS Photonics* **4**, 2203-2211 (2017).
98. Arora, K., Goel, N., Kumar, M. & Kumar, M. Ultrahigh performance of self-powered β-Ga_2O_3 thin film solar-blind photodetector grown on cost-effective Si substrate using high-temperature seed layer. *ACS Photonics* **5**, 2391-2401 (2018).
