# Supplementary material for: Ultrahigh-sensitivity adaptive solar-blind organic neuromorphic sensors for robust corona discharge monitoring
Source: Nat Commun. 2026 Sep 4;17:10510. doi: 10.1038/s41467-026-77523-y (PMC13639085; doi:10.1038/s41467-026-77523-y)
Supplement: Supplementary file 2 [file 41467_2026_77523_MOESM2_ESM.pdf]

nature portfolio

Peer Review File

Ultrahigh-Sensitivity Adaptive Solar-Blind Organic Neuromorphic Sensors for Robust Corona Discharge Monitoring

Corresponding Author: Professor Deyang Ji

This file contains all reviewer reports in order by version, followed by all author rebuttals in order by version.

Version 0:

Reviewer comments:

Reviewer #1

(Remarks to the Author)
Liu and co-authors reported an ultrahigh-sensitivity adaptive organic neuromorphic solar-blind ultraviolet sensor for corona discharge monitoring, and this work systematically demonstrated the excellent weak-light detection capability, outstanding optoelectronic performance, and adaptive visual adaptation characteristics of the solution-shearing-fabricated device. This study provides a crucial proof-of-concept for the intelligent safety monitoring of high-voltage power systems. However some issues need to address before publication.
1. The caption of Figure 1 is too wordy and is recommended to be simplified.
2. Some data point labels in Figure 2 are overcrowded and revision is recommended.
3. The unit labels of optical power density in Figure 4 are inconsistent and need to be unified.
4. The discussion section fails to mention the limitations of this study, such as the applicable scenarios of the device, stability under extreme environments, and uniformity of large-area fabrication. It is suggested to briefly supplement the deficiencies of this work and the prospects for future improvement, so as to enhance the academic completeness and rigor of the paper.
5. The extraction of fast response time should be defined.
6. According to the output curve shown in supplementary Fig.6 i-j, the high mobility device demonstrate certain contact issue, please explain it.

Reviewer #2

(Remarks to the Author)
The manuscript reports an organic solar-blind ultraviolet photosensor with integrated neuromorphic functionalities. The manuscript is generally well organized, and the conclusions are clearly presented. The work demonstrates certain novelty in the field of organic photodetectors. However, several important issues regarding the validity of the “solar-blind” claim, the interpretation of the device physics, and the practical significance of the neuromorphic sensing system remain insufficiently addressed. The detailed comments are as follows:
1. The authors claim that the proposed device is a “solar-blind ultraviolet photosensor”. However, according to the absorption spectrum shown in Supplementary Fig. 1, the material exhibits absorption across the entire ultraviolet region, with a relatively strong absorption peak also present around 365 nm. Therefore, the evidence currently provided is insufficient to rigorously support the designation of the device as “solar-blind”. Moreover, the manuscript should provide comprehensive photoresponse spectra to substantiate the claimed spectral selectivity.
2. The manuscript simultaneously presents a relatively fast response speed in Supplementary Fig. 13 and much slower response dynamics in the synaptic behaviors. The authors should clarify the origin of this discrepancy. In addition, periodic I-t photoresponse measurements containing complete rise and decay processes under different light intensities should be provided to further evaluate the dynamic response characteristics.
3. Although the manuscript demonstrates distinct electrical programming/optical erasing characteristics of the devices, the underlying working mechanism remains insufficiently understood. The authors should provide a more in-depth discussion of the charge trapping/detrapping processes and the physical origin of the memory behavior, supported by appropriate characterization techniques.

4. The discharge detection application design appears overly simplified. In particular, Fig. 5 seems to imply that the system is capable of locating the discharge position among different high-voltage components; however, such localization functionality is not actually demonstrated or validated in the manuscript. Moreover, for neuromorphic sensing systems, the use of simpler network architectures is generally more effective in highlighting the preprocessing efficiency of neuromorphic sensors themselves. In the current work, however, the device does not convincingly demonstrate its intrinsic processing efficiency within the discharge detection system. Instead, the task appears to rely heavily on an additional neural-network model.

Version 1:

Reviewer comments:

Reviewer #1

(Remarks to the Author)
The revised manuscript improved a lot with a clear demonstration on the application,detailed parameter comparison,additional selectivity evidence and thorough discussion. It is suitable for publication currently.

Reviewer #2

(Remarks to the Author)
The authors have carefully addressed all the concerns raised during the review process, and the additional experiments and discussions have significantly strengthened the manuscript. This work represents a good advancement in the field of solar-blind ultraviolet organic optoelectronic devices. I suggest that the authors cite the related work, Nature Communications (2022, 13(1): 6590), which is highly relevant to the present study and provides valuable context for the further development of solar-blind ultraviolet neuromorphic sensors.

Open Access This Peer Review File is licensed under a Creative Commons Attribution 4.0 International License, which permits use, sharing, adaptation, distribution and reproduction in any medium or format, as long as you give appropriate credit to the original author(s) and the source, provide a link to the Creative Commons license, and indicate if changes were made.
In cases where reviewers are anonymous, credit should be given to 'Anonymous Referee' and the source.
The images or other third party material in this Peer Review File are included in the article's Creative Commons license, unless indicated otherwise in a credit line to the material. If material is not included in the article's Creative Commons license and your intended use is not permitted by statutory regulation or exceeds the permitted use, you will need to obtain permission directly from the copyright holder.
To view a copy of this license, visit https://creativecommons.org/licenses/by/4.0/

For Referee #1

Dear Referee,

We greatly appreciated your encouraging and valuable suggestion for our manuscript (NCOMMS-26-029517). According to your comments, we revised our manuscript and made corresponding revisions marked red in the manuscript. Please find below our responses (in blue) to each of your specific comments (in black). Thanks again for your insightful comments!

Comment 1: The caption of Figure 1 is too wordy and is recommended to be simplified.

Our reply: Dear reviewer, thank you very much for your valuable suggestions. We have comprehensively streamlined and optimized the caption of Figure 1, removing duplicate and redundant modifying statements. While retaining all core experimental information, we have simplified the expression according to journal standards, making the caption concise, clear, and standardized. The revised captions have been updated in the revised manuscript.

Comment 2: Some data point labels in Figure 2 are overcrowded and revision is recommended.

Our reply: Dear reviewer, thank you for your correction. We have optimized and adjusted the data labels in Figure 2 by fine-tuning the label position, font size, and spacing to solve the problems of label overlap and crowding.

New figure:

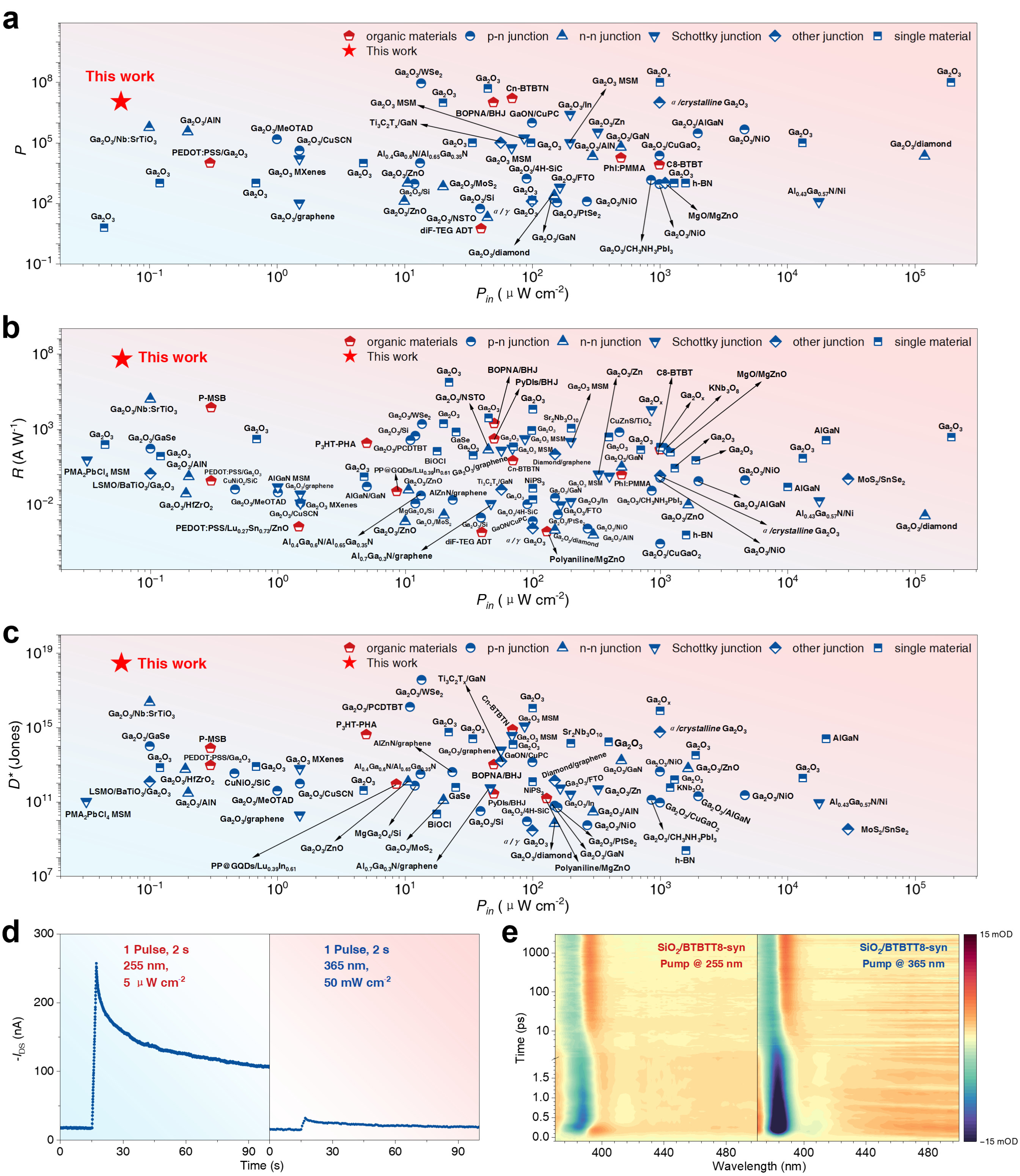

Fig. 2. Optical properties of BTBTT8-syn phototransistor. Comparison of lowest light intensity and **a,** *P*, **b,** *R* and **c,** D^* of the reported solar-blind ultraviolet photodetectors in recent years. **d**, Comparison of I-t measurement curves at 255 nm and 365 nm. **e**, Femtosecond Transient Absorption Spectroscopy (fs-TAS) pseudocolor plots of SiO_2/BTBTT8-syn thin films under excitation at pump wavelengths of 255 nm and 365 nm.

Comment 3: The unit labels of optical power density in Figure 4 are inconsistent and need to be unified.

Our reply: Dear reviewer, thank you for your professional suggestions. We have comprehensively verified and unified the unit labeling of all optical power densities in Figure 4, achieving consistent and standardized unit formats throughout the text. At the same time, we conducted a systematic check on the physical units of all charts throughout the text to avoid similar issues from happening again.

New figure:

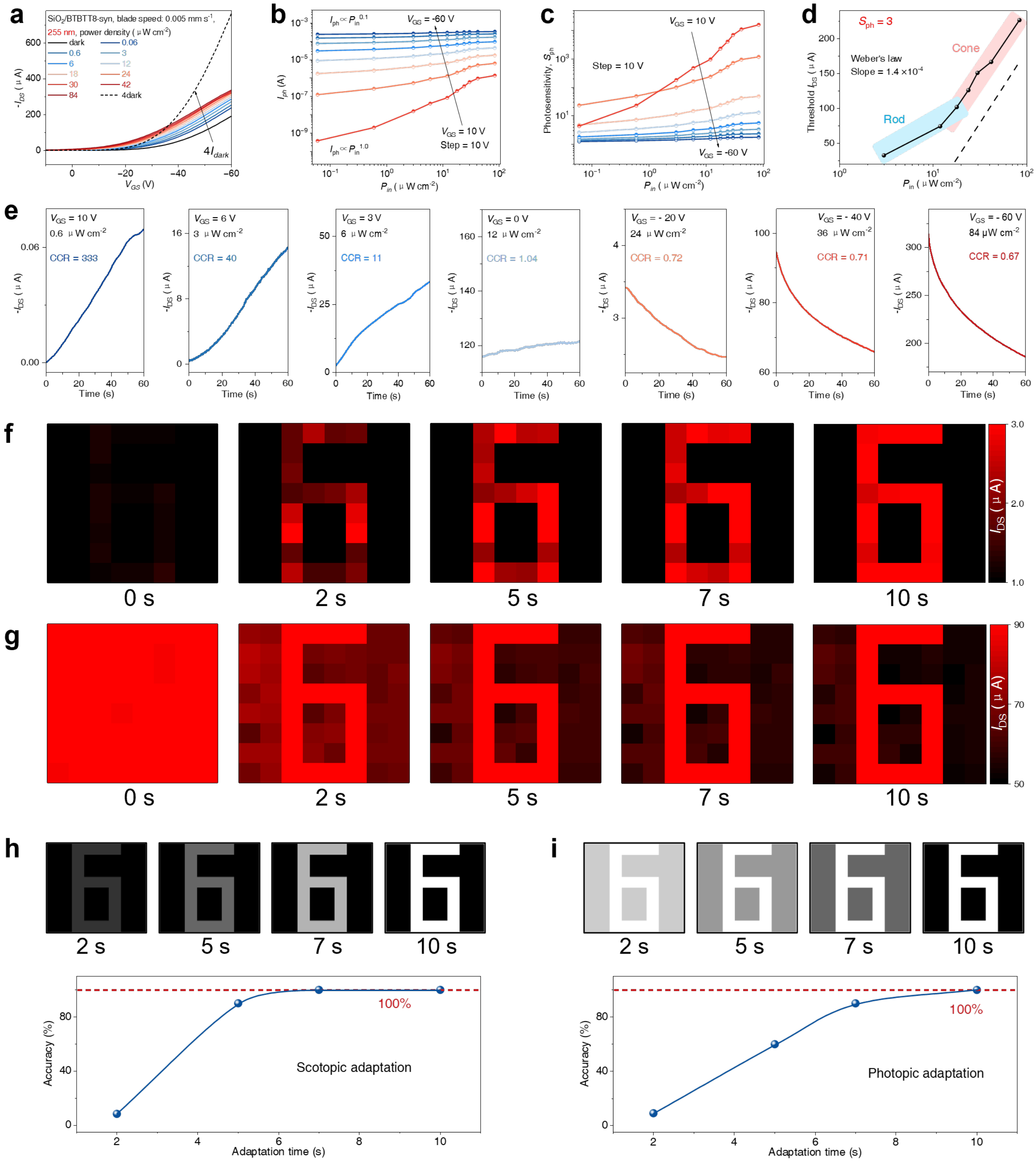

Fig. 4. Scotopic and photopic adaptation of phototransistor array. a, Transfer characteristic curves of the device measured

at V_{DS} = -60 V under different incident light power densities (P_{in}) values (255 nm wavelength). **b,** Photocurrent (I_{ph}) versus P_{in} for different V_{GS} values. **c,** Photosensitivity (S_{ph}) versus P_{in} for different V_{GS} values. **d,** Threshold I_{DS} as a function of P_{in} at S_{ph} = 3 extracted from a). **e,** Time-dependent current (I_{DS}) of the device under different V_{GS} values according to different P_{in} values. Time courses of **f,** scotopic and **g,** photopic adaptation for the pattern of "6". **h,** Top: the simulation schematic of scotopic adaptation by the OCR image "6". Bottom: recognition rate as a function of time for scotopic adaptation. **i,** Top: the simulation schematic of photopic adaptation by the OCR image "6". Bottom: recognition rate as a function of time for photopic adaptation.

Comment 4: The discussion section fails to mention the limitations of this study, such as the applicable scenarios of the device, stability under extreme environments, and uniformity of large-area fabrication. It is suggested to briefly supplement the deficiencies of this work and the prospects for future improvement, so as to enhance the academic completeness and rigor of the paper.

Our reply: Dear reviewer, we appreciate your constructive comments. The proposed device is only functional for corona signal monitoring under standard operating conditions. Its reliability under harsh environments such as high temperature and high humidity remains uncharacterized. In addition, all characterizations in this study were carried out on small-size devices, and the performance homogeneity of large-area thin films and batch-fabricated devices still needs further optimization. Future research will focus on refining manufacturing procedures, tuning interfacial architectures and upgrading packaging strategies to strengthen the environmental tolerance and large-area uniformity of the devices, facilitating their scalable implementation in intelligent power grid monitoring systems.

Comment 5: The extraction of fast response time should be defined.

Our reply: Dear reviewer, thank you for your professional suggestions. We have added the definition of fast response in the main text to make the description of response performance more standardized, clear, and explicit.

Our revision: In our revised manuscript, we added related contents. “Meanwhile, the device generates an

obvious instantaneous surge in photocurrent under irradiation with 25 ms light pulses, with a total recovery time as long as 400 s (Supplementary Fig. 15a), which originates from interfacial trap effect laying a solid physical basis for our subsequent investigation into synaptic mimicking functionalities. To systematically characterize the dynamic photoresponse of the device, periodic I-t measurements were conducted under three different light intensities (Supplementary Fig. 15b-d). In each test, five consecutive 25 ms light pulses were applied with a 20 s dark interval between adjacent pulses, which fully captured the rapid rise of photocurrent upon pulsed illumination as well as the complete slow decay process in the dark."

New figure:

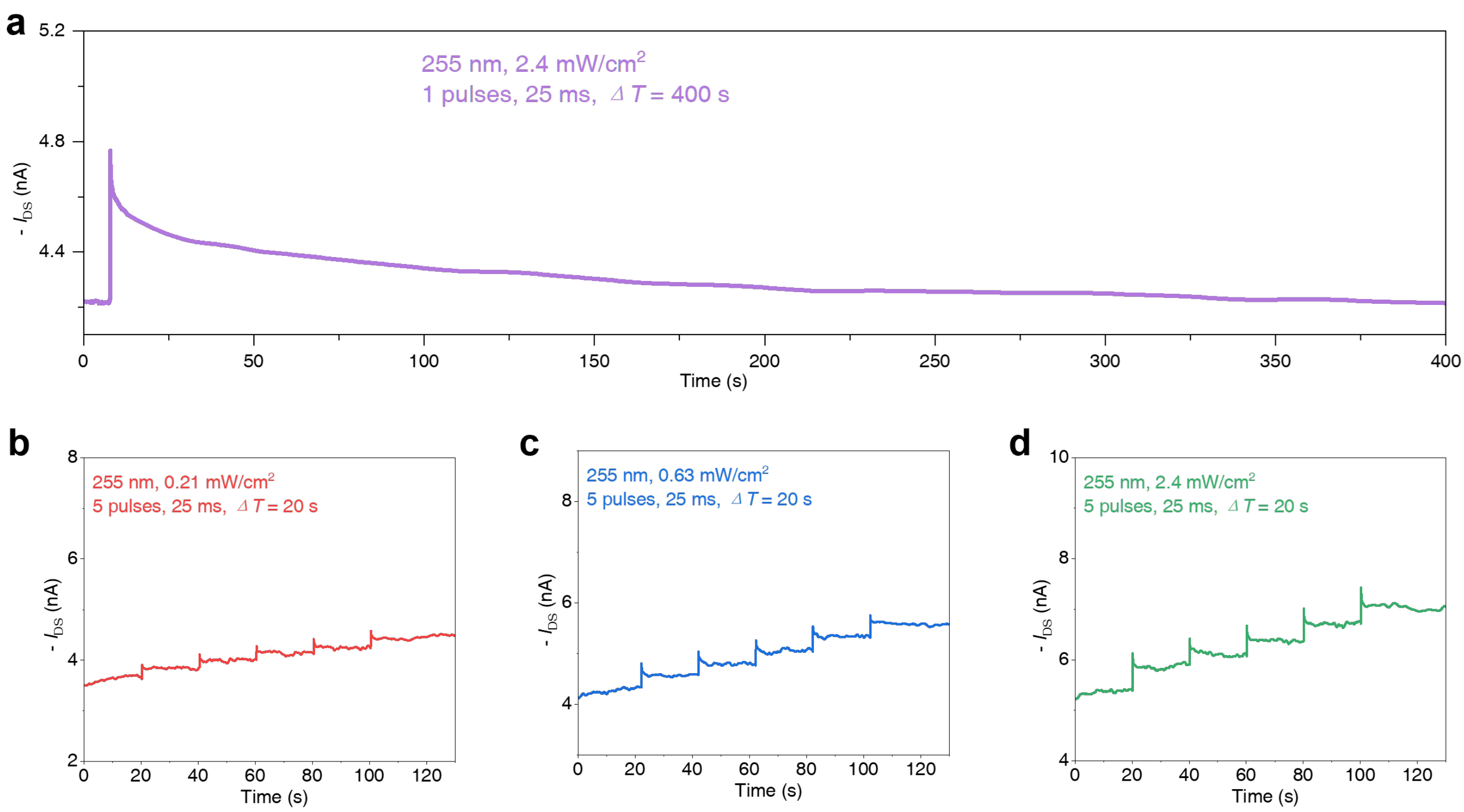

Supplementary Fig. 15. Tests of rapid response single pulse and 5 periodic pulses under different light intensities.

Comment 6: According to the output curve shown in supplementary Fig.6 i-j, the high mobility device demonstrates certain contact issue, please explain it.

Our reply: We thank the reviewer for this insightful comment. The apparent contact artifacts in output curves (Supplementary Fig. 6i-j) of high-mobility transistors stem from the relative weight of contact resistance

versus channel resistance: The total on-resistance equals the sum of source/drain contact resistance and intrinsic channel resistance. High carrier mobility drastically lowers intrinsic channel resistance, so contact resistance accounts for a large fraction of total resistance. A remarkable voltage drop occurs at metal-semiconductor contacts under drain current, reducing the effective bias on the channel. This leads to distorted linear-region slopes, suppressed saturation current and premature saturation in the output curves in Fig. 6i-j. In low-mobility devices, extremely large channel resistance overwhelms contact resistance, and contact-induced distortion can hardly be observed. We have added this analysis in the revised text to explain the contact behavior shown in the supplementary output characteristics.

For Referee #2

Dear Referee,

We greatly appreciated your encouraging and valuable suggestion for our manuscript (NCOMMS-26-029517). According to your comments, we revised our manuscript and made corresponding revisions marked red in the manuscript. Please find below our responses (in blue) to each of your specific comments (in black). Thanks again for your insightful comments!

Comment 1: The authors claim that the proposed device is a “solar-blind ultraviolet photosensor”. However, according to the absorption spectrum shown in Supplementary Fig. 1, the material exhibits absorption across the entire ultraviolet region, with a relatively strong absorption peak also present around 365 nm. Therefore, the evidence currently provided is insufficient to rigorously support the designation of the device as “solar-blind”. Moreover, the manuscript should provide comprehensive photoresponse spectra to substantiate the claimed spectral selectivity.

Our reply: Dear reviewer, we sincerely appreciate your rigorous and insightful suggestions. To systematically elucidate the discrepancies in device performance upon 255 nm and 365 nm excitation, we have supplemented a series of complementary characterizations in the revised manuscript.

Femtosecond Transient Absorption Spectroscopy (fs-TAS) was first adopted to unravel the carrier relaxation dynamics under the two excitation wavelengths. Time-resolved characterization quantitatively yields a carrier lifetime of 9.8 ns under 255 nm excitation, which is drastically longer than the 213 ps lifetime recorded upon 365 nm irradiation. Such a striking difference in carrier lifetime indicates that 255 nm illumination greatly favors exciton dissociation and long-lived separated charge carriers available for photoelectric conversion. By contrast, the ultrafast carrier recombination at 365 nm severely suppresses effective charge separation. Even though the device exhibits considerable optical absorption at 365 nm, its resultant photoelectric conversion efficiency is remarkably low. In sharp comparison, the efficient exciton dissociation, prolonged carrier lifetime

and unimpeded charge transport under 255 nm excitation collectively confer the device with superior photoresponse sensitivity.

We further conducted quantitative full-spectrum photoresponse tests to directly verify the wavelength selectivity of the device. The sensor can stably capture ultraweak 255 nm light signals down to 60 nW/cm^2 . However, milliwatt-scale 365 nm irradiation is mandatory to produce recognizable electrical signals, which explicitly proves that its response to longer-wavelength UV light is drastically suppressed.

Notably, the core innovation of this work lies in ultraweak light detection within the 255 nm solar-blind region, and our device delivers far superior weak-light sensing capability compared with traditional ultraviolet photodetectors. Collectively, the fs-TAS kinetic analysis and spectral photoresponse data solidly confirm the strong spectral preference for the 255 nm waveband, fully justifying its classification as a 255 nm solar-blind ultraviolet photodetector. All the experimental data and mechanistic discussions have been integrated into the revised manuscript.

Our revision: In our revised manuscript, we added related contents. "We next systematically characterize the spectral selectivity of the corresponding photodetector. Although BTBTT8-syn inherently absorbs 365 nm ultraviolet light, the device exhibits nearly negligible photoresponse at this wavelength. A remarkably high-power density threshold is required to generate measurable electrical signals under 365 nm excitation, which yields P, R, and D^* values of 2.1×10^5, 3.2×10^3 A W^{-1} and 4.1×10^{12} Jones, respectively (**Supplementary Fig. 12**). These figures are 2-6 orders of magnitude inferior to the performance metrics obtained under 255 nm solar-blind illumination. Comparative I-t transient measurements (**Fig. 2d**) further corroborate the device's outstanding wavelength discrimination capability. Weak 255 nm light at only 5 μW cm^{-2} triggers stable, well-resolved photocurrent signals; by contrast, intense 365 nm irradiation up to 50 mW cm^{-2} merely produces faint electrical outputs. To uncover the dynamic carrier origin underlying such drastically divergent photoresponse behaviors, excitation-dependent femtosecond transient absorption spectroscopy (fs-TAS) measurements were performed (**Fig. 2e, Supplementary Figs. 13a and 13b**). The ground-state bleaching (GSB) signal at probe

wavelength of 385 nm was extracted to evaluate the lifetime of photogenerated carriers. As shown in **Supplementary Figs. 13c and 13d**, time traces of GSB signal can be well-fitted by sum of three exponential functions, leading to time constants listed in **Supplementary Table 7**. The extracted fast (τ_1, τ_2) and slow (τ_3) components might associate with the free and trapped carriers, respectively. The GSB signal upon 255 nm excitation exhibits longer carrier lifetimes than corresponding time constants extracted from 365 nm illumination (**Supplementary Figs. 13c, 13d and Supplementary Table 7**). This spectroscopic evidence unambiguously verifies the intrinsic preference of BTBTT8-syn for efficient photoresponse under 255 nm solar-blind UV excitation. Taken together, the device achieves precise recognition of 255 nm optical signals with ultrahigh spectral specificity, while effectively suppressing interfering responses originating from 365 nm long-wave ultraviolet light."

New figure:

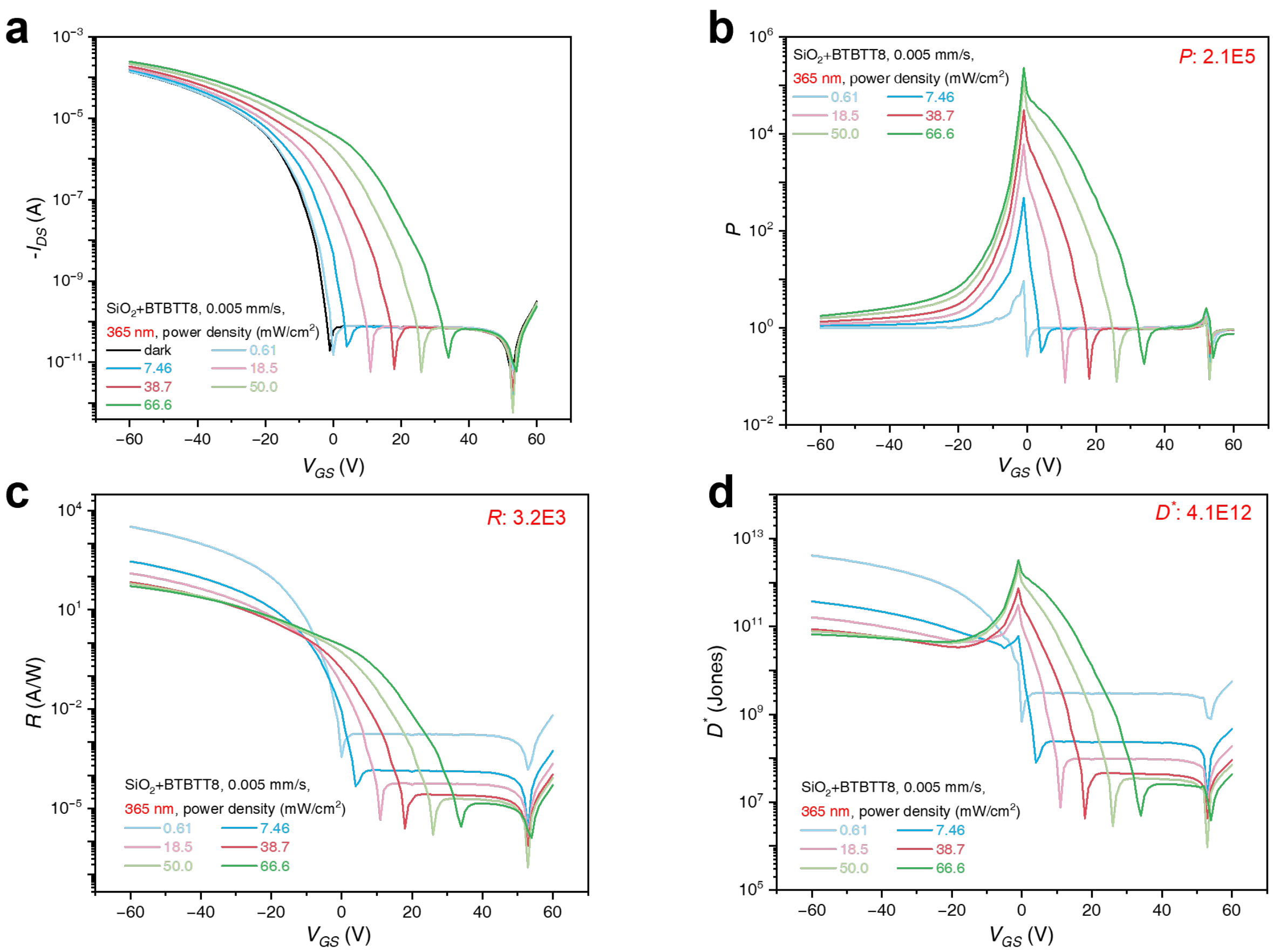

Supplementary Fig. 12. Spectral selectivity of devices. a, The light response curve and corresponding **b,** *P*, **c,** *R*, **d,** D^* curves of the device at 365nm.

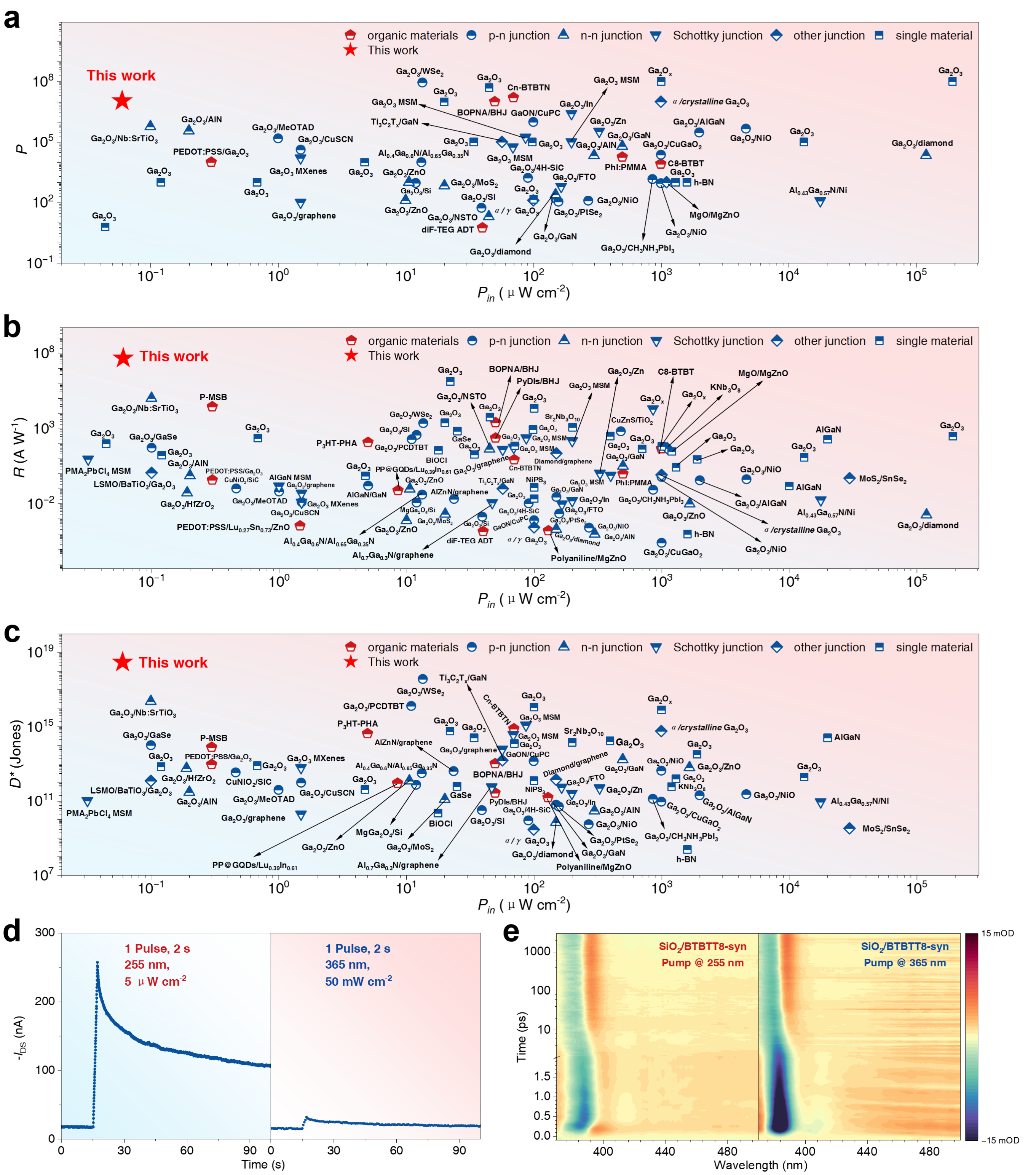

Fig. 2. Optical properties of BTBTT8-syn phototransistor. Comparison of lowest light intensity and **a,** P, **b,** R and **c,** D^* of the reported solar-blind ultraviolet photodetectors in recent years. **d**, Comparison of I-t measurement curves at 255 nm and 365 nm. **e**, Femtosecond Transient Absorption Spectroscopy (fs-TAS) pseudocolor plots of SiO_2/BTBTT8-syn thin films under excitation at pump wavelengths of 255 nm and 365 nm.

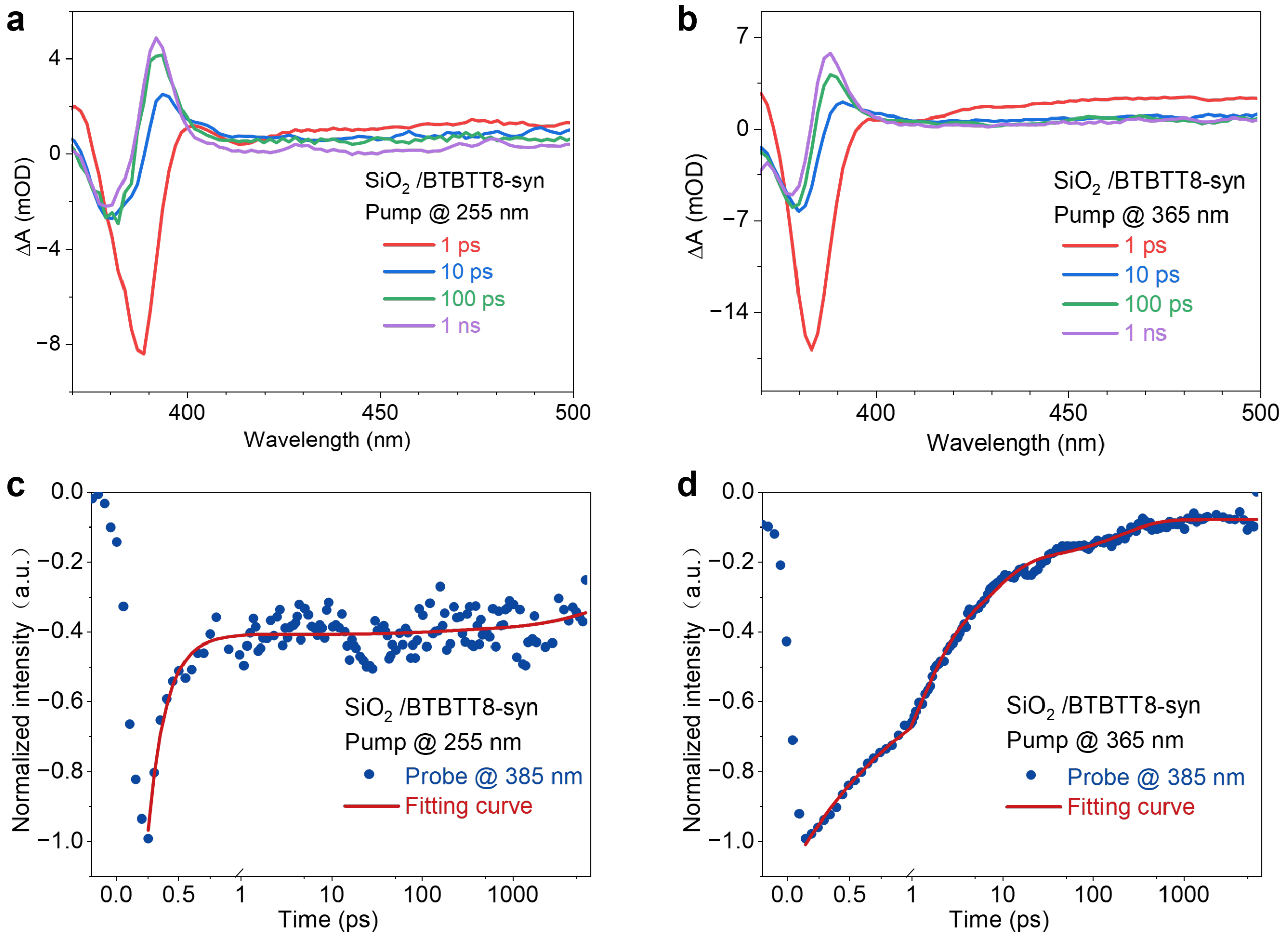

Supplementary Fig. 13. Femtosecond Transient Absorption Spectroscopy (fs-TAS) measurement. fs-TAS spectra at **a**, Pump @ 255nm and **b**, Pump @ 365nm. Normalized GSB curves extracted with a 385 nm probe, with panel **c**, excited at 255 nm and panel **d**, excited at 365 nm.

New table:

Supplementary Table 7. Extracted decay time constants of ground-state bleaching signal of syn BTBTT8 under different excitation wavelengths

Excitation wavelength (nm)	Lifetime
255	τ_1 = 140 fs<br>τ_2 = 163 ps<br>τ_3 = 9.80 ns
365	τ_1 = 1.00 ps<br>τ_2 = 7.60 ps<br>τ_3 = 213 ps

Comment 2: The manuscript simultaneously presents a relatively fast response speed in Supplementary Fig. 13 and much slower response dynamics in the synaptic behaviors. The authors should clarify the origin of this

discrepancy. In addition, periodic I-t photoresponse measurements containing complete rise and decay processes under different light intensities should be provided to further evaluate the dynamic response characteristics.

Our reply: Dear reviewer, we sincerely appreciate your valuable comments and suggestions. Under 255 nm solar-blind UV excitation, efficient exciton dissociation generates long-lived free charge carriers with nanosecond-scale relaxation lifetimes. Combined with the decent charge mobility of BTBTT8-syn thin films, most photogenerated carriers can be rapidly transported and collected by electrodes, thereby enabling prominent, sensitive photoresponse at 255 nm. Nevertheless, abundant defect states at the interface of the thin film and SiO_2 act as charge trapping centers. A portion of photocarriers is captured and stored in these traps during illumination. After switching off the incident light, trapped charges gradually escape via thermal activation at an extremely slow rate, which accounts for the ultralong decay/recovery time of the photocurrent signal. In addition, we further supplemented gradient-intensity periodic I-t tests with sequential 25 ms pulses separated by 20 s.

All figures are uniformly renumbered, with these transient curves categorized as Supplementary Fig. 15 and all textual figure citations updated.

Our revision: In our revised manuscript, we added related contents. “Meanwhile, the device generates an obvious instantaneous surge in photocurrent under irradiation with 25 ms light pulses, with a total recovery time as long as 400 s (**Supplementary Fig. 15a**), which originates from interfacial trap effect laying a solid physical basis for our subsequent investigation into synaptic mimicking functionalities. To systematically characterize the dynamic photoresponse of the device, periodic I-t measurements were conducted under three different light intensities (**Supplementary Fig. 15b-d**). In each test, five consecutive 25 ms light pulses were applied with a 20 s dark interval between adjacent pulses, which fully captured the rapid rise of photocurrent upon pulsed illumination as well as the complete slow decay process in the dark.”

New figure:

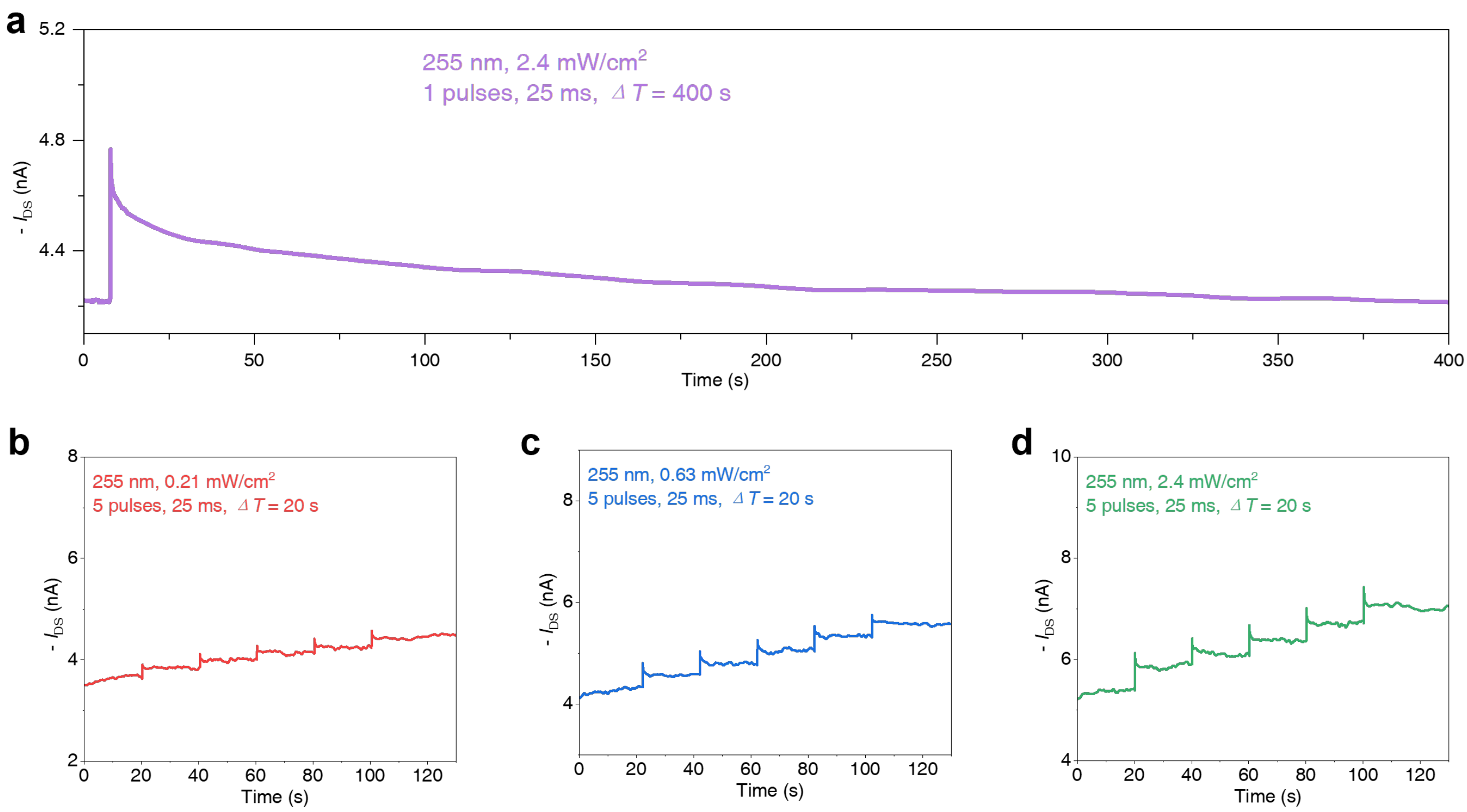

Supplementary Fig. 15. Five periodic pulse and recovery time tests for rapid response under different light intensities.

Comment 3: Although the manuscript demonstrates distinct electrical programming/optical erasing characteristics of the devices, the underlying working mechanism remains insufficiently understood. The authors should provide a more in-depth discussion of the charge trapping/detrapping processes and the physical origin of the memory behavior, supported by appropriate characterization techniques.

Our reply: Dear reviewer, we sincerely appreciate your professional and constructive comment. As recommended, we have substantially expanded and deepened the mechanistic discussion underlying the device memory performance. By integrating established physical models of organic memory transistors with electron paramagnetic resonance (EPR) characterizations (Supplementary Fig. 19), we systematically dissect the full charge trapping and photo-triggered detrapping kinetics. All relevant mechanistic analyses have been fully integrated into the revised manuscript.

Our revision: In our revised manuscript, we added related contents. “The reversible optoelectronic memory

performance of the device stems from intrinsic defect-mediated charge trapping within the SiO_2 dielectric layer. Previous literature has confirmed a variety of native defects in SiO_2, such as oxygen vacancies and interstitial oxygen atoms[37-42]. In this work, electron paramagnetic resonance (EPR) measurements directly verify the existence of oxygen vacancy defects inside the SiO_2 film (**Supplementary Fig. 19**). In addition, quantitative analysis based on the subthreshold swing extracts a high interfacial trap density of 1.2×10^{13} cm^{-2} eV^{-1}, which solidly confirms abundant charge-trapping sites at the dielectric-semiconductor interface. For our blade-coated heterostructure device consisting of SiO_2 and BTBTT8-syn, negative gate bias drives holes in the BTBTT8-syn channel to inject into the defect energy levels of SiO_2 and become permanently confined. The depletion of channel carriers shifts the transfer characteristic curve toward negative voltages, corresponding to the electrical programming process. Upon illumination with 255 nm solar-blind UV light, the BTBTT8-syn active layer absorbs incident photons and generates abundant photocarriers. The resulting photoelectrons inject into the SiO_2 dielectric and neutralize the pre-trapped holes, which enables gradual detrapping and release of stored charges. As a result, the transfer curve recovers to its original state, accomplishing optical erasure. Relying on this distinctive reversible optoelectronic memory mechanism, we further investigate the feasibility of the device for biomimetic synaptic emulation toward biological visual information processing."

…

37. Lan, G. *et al.* Organic photonic synapses for application of bioinspired neuromorphic visual perception and beyond. *Adv. Funct. Mater.* **36**, 10.1002/adfm.202522513 (2025).
38. Zheng, Y. *et al.* Introducing pinMOS memory: A novel, nonvolatile organic memory device. *Adv. Funct. Mater.* **30**, 1907119 (2019).
39. Jiang, T. *et al.* Wafer-Scale organic neuromorphic sensors for programmable displaying. *Adv. Mater.* **38**, 10.1002/adma.202522289 (2026).
40. Li, Q. *et al.* Multifunctional neuromorphic vision enabled by photo-regulated radical. *Adv. Mater.* **38**, 10.1002/adma.73363 (2026).
41. Yi, M. *et al.* Light programmable/erasable organic field-effect transistor ambipolar memory devices based on the pentacene/PVK active layer. *J. Mater. Chem. C* **3**, 5220-5225 (2015).
42. Van De Burgt, Y. *et al.* Organic electronics for neuromorphic computing. *Nat. Electron.* **1**, 386-397 (2018).

New figure:

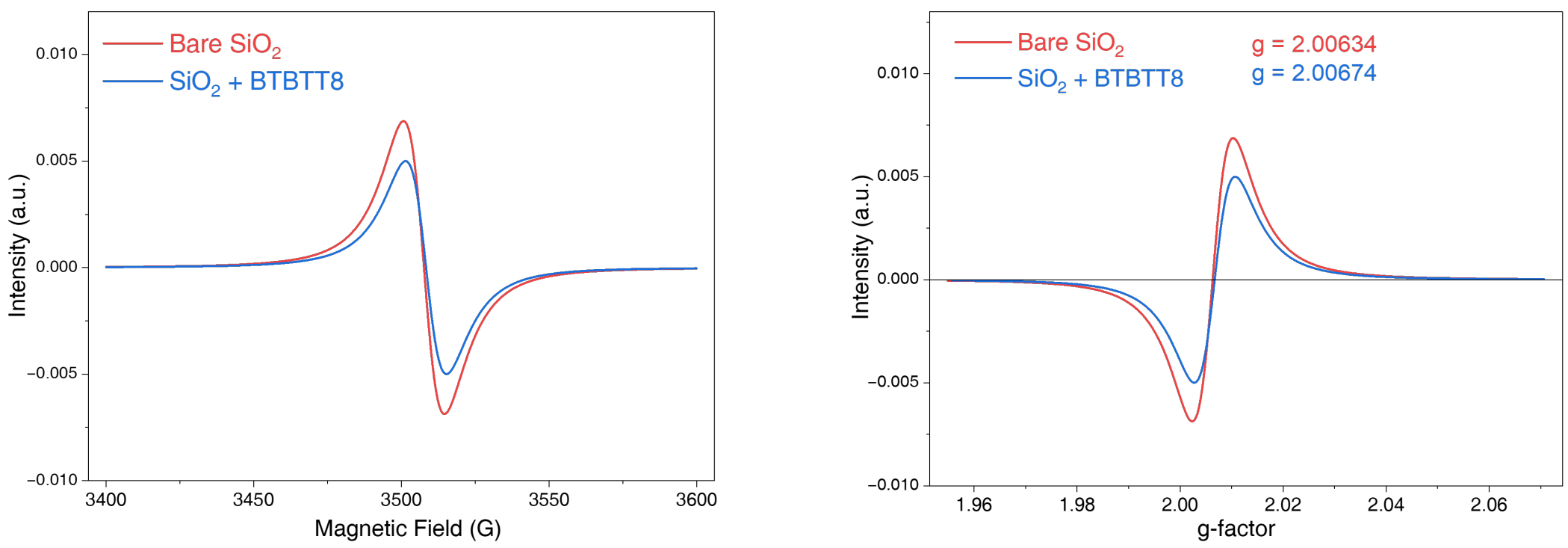

Supplementary Fig. 19. Electron paramagnetic resonance (EPR) oxygen vacancy testing and g-value at different interfaces.

Comment 4: The discharge detection application design appears overly simplified. In particular, Fig. 5 seems to imply that the system is capable of locating the discharge position among different high-voltage components; however, such localization functionality is not actually demonstrated or validated in the manuscript. Moreover, for neuromorphic sensing systems, the use of simpler network architectures is generally more effective in highlighting the preprocessing efficiency of neuromorphic sensors themselves. In the current work, however, the device does not convincingly demonstrate its intrinsic processing efficiency within the discharge detection system. Instead, the task appears to rely heavily on an additional neural-network model.

Our reply: Dear Reviewer, we sincerely appreciate your careful reading and insightful critical comments. We address your two major concerns point by point as follows:

First, regarding the misleading implication of discharge localization in Figure 5. We have revised the confusing schematic and supplemented explicit textual explanations in the revised manuscript. The original diagram merely displayed several typical high-voltage components prone to corona discharge as schematic illustrations, rather than demonstrating a spatial discharge localization function. The core focus of our work lies in ultra-sensitive solar-blind UV signal detection, instead of locating the spatial position of discharge sources. This revision eliminates the misleading information as you pointed out.

Second, in response to your comment that the current system fails to sufficiently highlight the intrinsic preprocessing efficiency of the neuromorphic sensor and seems to overly rely on auxiliary neural network

models. In line with your suggestion that lightweight network architectures are more suitable for reflecting the native signal processing merits of neuromorphic sensors, we adopt a compact simple network as our primary classification model for core verification.

All neural network simulations are built entirely on real electrical response data measured from our device (Figure 5b), rather than purely theoretical input. We first constructed a lightweight three-layer fully connected perceptron without self-attention or residual modules. As presented in Figure 5c – d and Supplementary Fig. 26, this streamlined network still achieves a high average classification accuracy of 95.5% using identical raw sensor signals. This result solidly proves that our neuromorphic sensor itself can extract effective discharge features and suppress ambient noise, delivering powerful intrinsic preprocessing capability and requiring no complicated backend network for qualified recognition performance.

We additionally reserve an attention-augmented complex MLP equipped with self-attention and residual blocks only as an engineering control group. This advanced model only marginally lifts the average accuracy to 96.5% with negligible performance improvement. Its main value lies in enhancing the long-term operational stability of the high-voltage early-warning system, rather than compensating for insufficient signal processing capacity of the sensor.

Our revision: In our revised manuscript, we added related contents. “We first established a complete experimental dataset based on real-time electrical signals collected from the device, covering sixteen distinct types of characteristic corona discharge waveforms. The sensor generates nearly negligible photocurrent under visible illumination at 425 nm, 515 nm and 690 nm, which corroborates its outstanding anti-interference capability and precise 255 nm selective detection performance. The collected discharge signals fall into three representative categories: short-interval pulse discharges containing three consecutive pulses (Categories 2-6), long-interval pulse discharges with five sequential pulses (Categories 7-11), and continuous 500 ms steady discharges (Categories 12-16). Corresponding excitation light intensities for these discharge modes are 0.06, 0.19, 1.4, 2.3 and 4.5 μW cm^{-2}, respectively. The device output photocurrent increases monotonically with

rising incident irradiance (**Fig. 5b**), providing distinguishable feature information for subsequent classification tasks.

To verify the inherent signal preprocessing superiority of the neuromorphic sensor, we developed a compact three-layer fully connected perceptron as the core lightweight classification framework, whose architecture is displayed in **Fig. 5c**. This model adopts a 2-neuron input layer to receive two-dimensional signal features captured directly by the sensor. Free of self-attention and residual structures, it realizes efficient feature transformation via two activated fully connected layers and completes the classification of sixteen discharge signal patterns. We partitioned the dataset by allocating the first 6000 samples of each category for model training and the rest for testing. After sufficient iterative optimization, the single-category classification accuracy stabilizes above 80% (**Fig. 5d and Supplementary Fig. 26a**), and the overall average classification accuracy reaches 95.5% (**Supplementary Fig. 26b**). These results fully demonstrate that the proposed sensor can independently extract faint corona discharge signatures and suppress environmental noise, thereby exhibiting prominent native signal preprocessing efficiency for discharge monitoring. On this basis, we further constructed an attention-augmented multi-layer perceptron (Attention-Augmented MLP) integrated with self-attention and residual modules for practical high-voltage discharge early-warning applications; its detailed network configuration is illustrated in **Fig. 5e**. This complex network only serves as an engineering auxiliary scheme to improve system stability, while the high classification accuracy of the lightweight perceptron confirms that reliable discharge identification fundamentally relies on the superior signal output quality of our neuromorphic sensor itself."

New figure:

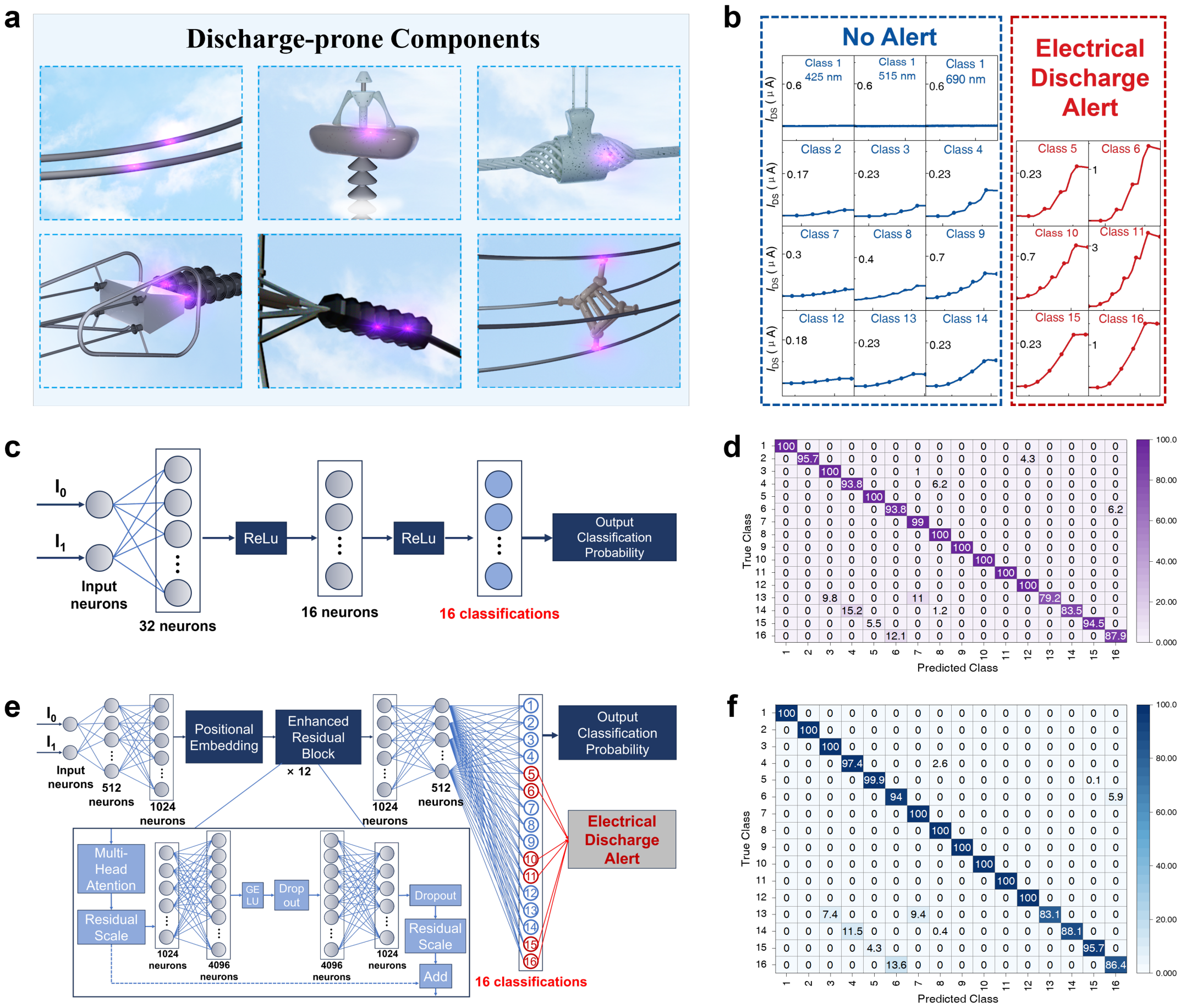

Fig. 5. High-voltage discharge detection system for corona discharge early warning. a, Schematic illustration of discharge detection and main discharge components. **b,** Actual current signals corresponding to classification 16. **c,** Schematic illustration of the simple architecture network model. **d,** The confusion matrix comparing the predicted classification and the actual classification after the simulation calculation of the simple model. **e,** Schematic illustration of the Attention-Augmented MLP model system architecture. **f,** The confusion matrix of the predicted classification and the actual classification obtained through simulation and calculation by the Attention-Augmented MLP model.

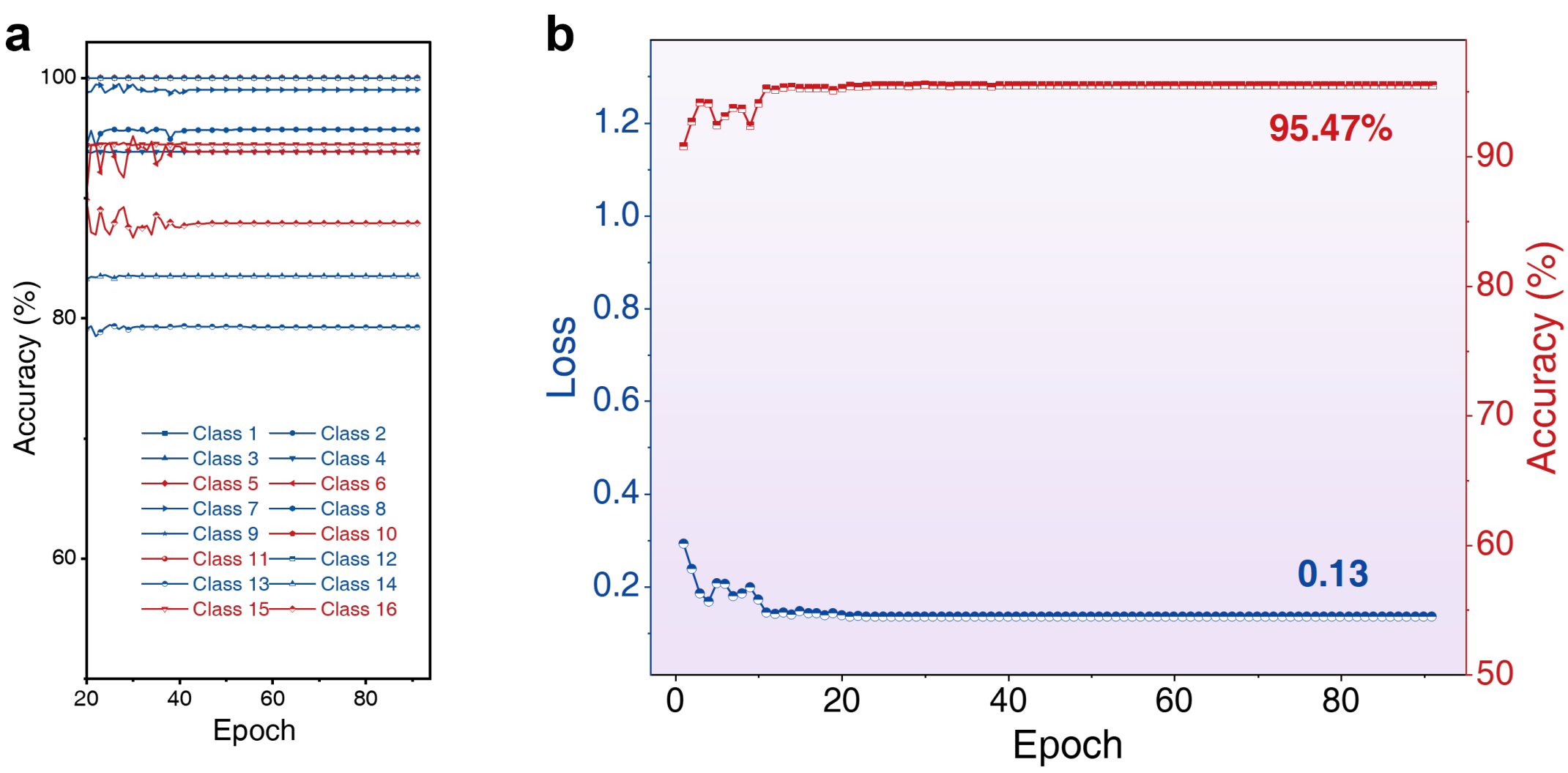

Supplementary Fig. 26. 16-Class classification accuracy and average classification accuracy of the simplified model. a, Classification accuracy of 16 classes after 91 epochs. **b,** Average classification accuracy and loss of the 16 classes after 91 epochs.

For Referee #2

Dear Referee,

We greatly appreciated your encouraging and valuable suggestion for our manuscript (NCOMMS-26-029517A-Z). According to your comments, we revised our manuscript and made corresponding revisions marked red in the manuscript. Please find below our responses (in blue) to each of your specific comments (in black). Thanks again for your insightful comments!

Comment 1: The authors have carefully addressed all the concerns raised during the review process, and the additional experiments and discussions have significantly strengthened the manuscript. This work represents a good advancement in the field of solar-blind ultraviolet organic optoelectronic devices. I suggest that the authors cite the related work, Nature Communications (2022, 13(1): 6590), which is highly relevant to the present study and provides valuable context for the further development of solar-blind ultraviolet neuromorphic sensors.

Our reply: Dear reviewer, thank you for your rigorous and valuable comments. The suggested reference (Nature Communications, 2022, 13(1): 6590) has been added to the manuscript, which provides valuable context for our present work.

Our revision: Solar-blind ultraviolet (SBUV) detection has attracted substantial attention across both military and civilian domains[1], with its application scenarios encompassing missile warning and tracking, corona discharge monitoring,

…

1. Zhang, Z. *et al*. In-sensor reservoir computing system for latent fingerprint recognition with deep ultraviolet photo-synapses and memristor array. *Nat. Commun.* 13, 6590 (2022).
